# Impact of metformin treatment during pregnancy on maternal outcomes: a systematic review/meta-analysis

**DOI:** 10.1038/s41598-021-88650-5

**Published:** 2021-04-29

**Authors:** Jane L. Tarry-Adkins, Susan E. Ozanne, Catherine E. Aiken

**Affiliations:** 1 Metabolic Research Laboratories and MRC Metabolic Diseases Unit, Wellcome Trust-MRC Institute of Metabolic Science, University of Cambridge, Cambridge, UK; 2 Department of Obstetrics and Gynaecology, The Rosie Hospital and NIHR Cambridge Biomedical Research Centre, University of Cambridge, Cambridge, UK

**Keywords:** Diseases, Endocrinology

## Abstract

We systematically assessed the impact of metformin treatment on maternal pregnancy outcomes. PubMed, Ovid Embase, Medline, Web of Science, ClinicalTrials.gov and Cochrane databases were systematically searched (inception-1st February 2021). Randomised controlled trials reporting pregnancy outcomes in women randomised to metformin versus any other treatment for any indication were included. Outcomes included gestational weight gain (GWG), pre-eclampsia, gestational hypertension, preterm birth, gestational age at delivery, caesarean section, gestational diabetes, glycaemic control, and gastrointestinal side-effects. Two independent reviewers conducted screening, with a third available to evaluate disagreements. Risk-of-bias and GRADE assessments were conducted using Cochrane Risk-of-Bias and GRADE-pro software. Thirty-five studies (n = 8033 pregnancies) met eligibility criteria. GWG was lower in pregnancies randomised to metformin versus other treatments (1.57 kg ± 0.60 kg; I_2_ = 86%, *p* < 0.0001), as was likelihood of pre-eclampsia (OR 0.69, 95% CI 0.50–0.95; I_2_ = 55%, *p* = 0.02). The risk of gastrointestinal side-effects was greater in metformin-exposed versus other treatment groups (OR 2.43, 95% CI 1.53–3.84; I_2_ = 76%, *p* = 0.0002). The risk of other maternal outcomes assessed was not significantly different between metformin-exposed versus other treatment groups. Metformin for any indication during pregnancy is associated with lower GWG and a modest reduced risk of pre-eclampsia, but increased gastrointestinal side-effects compared to other treatments.

## Introduction

Metformin, an oral insulin-sensitizing and glucose-lowering drug, is widely prescribed during pregnancy. Guidelines from several global contexts, including the UK^1,2^, New Zealand^3^ and Canada^4^ endorse metformin for the treatment of gestational diabetes mellitus (GDM). In the US, guidelines from the ADA and SMFM differ in their recommendations regarding the use of metformin as a first-line agent in pregnancy primarily because metformin crosses the placenta to the fetus^5^. In low resource obstetric settings where some of the greatest increases in GDM are currently observed^6^, metformin is commonly used in pregnancy it is relatively inexpensive and easily stored. Metformin is also used in pregnancy for other conditions, including pre-existing diabetes^7^ and polycystic ovarian syndrome (PCOS)^8^, and has been trialled in the context of maternal obesity^9^. However, despite the widespread prescription of metformin during pregnancy, data regarding maternal pregnancy outcomes are relatively sparse. In light of the recent ADA guidelines and meta-analyses specifically comparing GDM treatments^10–13^, some clinicians are beginning to adopt a more cautious approach^14^.

In the context of GDM, our previous study shows that metformin provides adequate glycaemic control in approximately two-thirds of patients who require pharmacological therapy, with the remaining 14–46% requiring additional treatment, usually insulin^12^. Advantages of metformin include oral administration, cost-effectiveness, and suitability for use in low-resource settings^15^. However, previous meta-analyses have raised concerns about the impacts of metformin on fetal and post-natal growth in the context of GDM^12,13^. However maternal pregnancy outcomes are less well-studied, in women treated for conditions other than GDM^16,17^. Randomised trials have reported potential maternal benefits associated with metformin treatment, including reduced gestational weight gain (GWG)^18,19^. Furthermore, mixed evidence suggests that pre-eclampsia rates may be reduced in women randomised to metformin treatment^20,21^.

Given the increasingly high number of women currently being prescribed metformin during pregnancy, we evaluated the impact of metformin treatment in pregnancy on the mother, by synthesizing all available randomised trial data pertaining to common maternal outcomes (including gestational weight gain, pre-eclampsia, gestational hypertension, pre-term birth, gestational age at delivery, caeserean-section, glycaemic control, adverse events and GDM). These were investigated across the range of indications for which metformin is currently prescribed or trialled in pregnancy.

## Materials and methods

This systematic review and meta-analysis was conducted in accordance with the Preferred Reporting Items for Systematic Reviews and Meta-Analyses (PRISMA) guidelines^22^. The systematic review protocol was registered prospectively in PROSPERO CRD42020167692 on 14th February 2021 (Supplementary Text S1) prior to data collection. Ethical approval was not required for this meta-analysis.

### Literature searches, search strategies and eligibility criteria

Systematic literature searches using pre-specified terms (Supplementary Text S2) were performed on PubMed, Ovid EMBASE, Ovid Medline, Cochrane library, Clinicaltrials.gov, and Web of Science from database inception dates to 14th February 2021. No language or location restrictions were applied. Studies that randomised pregnant women to metformin (not in combination with any other drug) versus any other drug treatment, placebo, or no treatment were included. Studies were included if they randomised pregnant women for any indication (including GDM, pre-existing diabetes, PCOS, or maternal obesity). All treatment indications were screened for and diagnosed according to local criteria in each study, and we did not apply exclusions with respect to this. Studies were excluded if they included participants with multiple pregnancies or if they randomised fewer than 50 women in total. Data reported in abstracts at society meetings or conferences would have been included if the abstract contained sufficient information for assessment, but none fulfilled the criteria. Where insufficient information for assessment was available, authors were contacted for further information. One study provided insufficient information for assessment, however the authors did not respond to contact and therefore this study could not be included.

### Study selection and data extraction

Two reviewers (JLA and CEA) independently assessed each study using pre-determined inclusion/exclusion criteria (Supplementary Table S2). A third reviewer (SEO) was available to resolve cases where eligibility was unclear. An initial screen of titles and abstracts was performed, followed by a detailed full paper screen (Supplementary Fig. S1).

Data extraction from eligible studies was conducted independently using a standardised proforma by two authors (JLA and CEA). Maternal outcome measures were: gestational weight gain (GWG, throughout pregnancy; kg), pre-eclampsia, gestational hypertension (PIH), preterm birth (divided into spontaneous and iatrogenic), gestational age at delivery (weeks), caesarean section rates (divided into elective and emergency), glycaemic control (fasting blood glucose, FBG; mg/dL and random blood glucose, RBG; mg/dL), new GDM incidence, maternal hypoglycaemia, and any reported side-effects. All outcome measures were defined as per the original study criteria, and we did not apply any exclusion with respect to these.

### Quality assessment (risk of bias)

Each study was independently assessed by two authors (JLA and CEA) for quality and validity using the Cochrane Collaboration tool for assessing risk of bias. Seven risk of bias domains were systematically assessed for each study and each domain was given a rating of low risk, unknown risk or high risk of bias (Supplementary Table S3). All risk of bias analysis was conducted at the study level.

The principle summary measures were unadjusted odds ratios (OR) (for dichotomous data) or differences in means (for continuous data). Meta-analysis was performed using Review Manager (RevMan) Version 5.3, Copenhagen: The Nordic Cochrane Centre, the Cochrane Collaboration, 2014) and the ‘*metafor*’ package in R version 3.5.1 (R Foundation for Statistical Computing, Vienna, Austria. Network meta-analysis was not implemented as the criteria for transitivity were not met by the set of included studies. Meta-regression was implemented to assess potential sources of inter-study heterogeneity (Supplementary Table S1). Funnel plots were constructed to assess publication bias. Meta-analyses with 5 or more studies included were also subjected to Egger’s test. Heterogeneity between studies was assessed using the I-squared statistic. Any outcomes demonstrating significant inter-study heterogeneity (heterogeneity *p* value < 0.05) were analysed using a random-effects model. Sensitivity analyses were performed using ‘leave-one-out’ sensitivity testing for individual studies. All outcomes were subjected to Grading of Recommendations, Assessment, Development and Evaluation (GRADE) analysis (GRADEpro Guideline Development Tool, McMaster University, USA). Where the indication for randomisation was diabetes in pregnancy, the included studies all compared metformin to either insulin or glyburide, hence further sub-group analyses were performed by comparator group. Where the indication for randomisation was PCOS or obesity, all included studies compared metformin to placebo, therefore no further sub-group analyses were performed. A further sensitivity analysis excluding studies in which analysis was not performed on an intention-to-treat basis was also conducted. Where *p* values are reported, an alpha level < 0.05 was considered statistically significant.

## Results

### Study selection and study characteristics

The PRISMA flow chart (Supplementary Fig. S1) demonstrates the screening procedure involved to attain 35 studies (8033 participants) for this meta-analysis. The majority of these studies (27 studies; 5319 participants) investigated metformin treatment for diabetes in pregnancy (25 of these were women with GDM only and 2 studies included women with any type of diabetes in pregnancy). A total of 8 studies (2714 participants) investigated maternal obesity (4 studies; n = 1485) or PCOS (3 studies; n = 930) or pre-gestational insulin resistance (1 study; n = 299). No studies investigated randomisation of metformin compared to diet/lifestyle intervention alone, however these were commonly implemented alongside other treatments. Eligible studies were identified comparing metformin to insulin, glyburide, and placebo. For all indications and comparisons, the studies varied with respect to quality and design (Supplementary Table S1). The included studies demonstrated considerable heterogeneity with respect to the dosage of pharmacological agents (Supplementary Table S4). Heterogeneity also existed in the diagnostic criteria used for GDM and PCOS (Supplementary Table S5). The included studies came from a variety of geographical locations: Australasia (Australia and New Zealand), Europe (the UK, Norway and Finland), North Africa/Middle East (Egypt, Iran and Israel) and the North America/Latin America (Canada, Mexico, Brazil and Chile).

### Risk of bias and sensitivity analyses

The risk of bias was moderate-to-low in the majority of included studies, however six studies were considered to have a high risk of bias (Supplementary Table S3). We performed sensitivity analyses, excluding the studies assessed as having a high risk of bias (Supplementary Fig. S2), which showed that removal of studies with a high risk of bias did not materially alter the outcome of the meta-analyses for any of the outcomes, therefore all studies were included. Most studies reported non-significant differences in maternal baseline characteristics between groups (including maternal age, BMI and glycaemic control) (Supplementary Table S4).

We assessed the likelihood of single studies significantly influencing the overall results using leave-one-out (LOO) analysis. For the primary comparison of metformin versus any other treatment across all indications, meta-analyses were robust to the omission of single studies (Supplementary Fig. S3), with the exception of caesarean section, RBS, and maternal hypoglycaemia, decreasing our confidence in the robustness of these findings. Funnel plots for all outcomes were assessed visually (Supplementary Fig. S4); there were no obvious asymmetries in the plots for any study outcomes. Eggers testing demonstrated a low likelihood of publication bias with respect to the primary comparisons (Supplementary Table S6).

### GRADE analysis (certainty of evidence)

The majority of outcomes were classified as having a moderate certainty of evidence (Supplementary Fig. S5; primary outcomes and Supplementary Fig. S6; secondary outcomes), with one outcome having a high certainty of evidence (gestational hypertension). The moderate certainty of evidence was ascertained from the majority of outcomes (5) having high heterogeneity (inconsistency) of studies. All studies had no detected publication bias (as ascertained by the Eggers testing and funnel plot analyses). All studies reported direct evidence.

### Synthesis of results

#### Gestational weight gain

**Figure 1:**
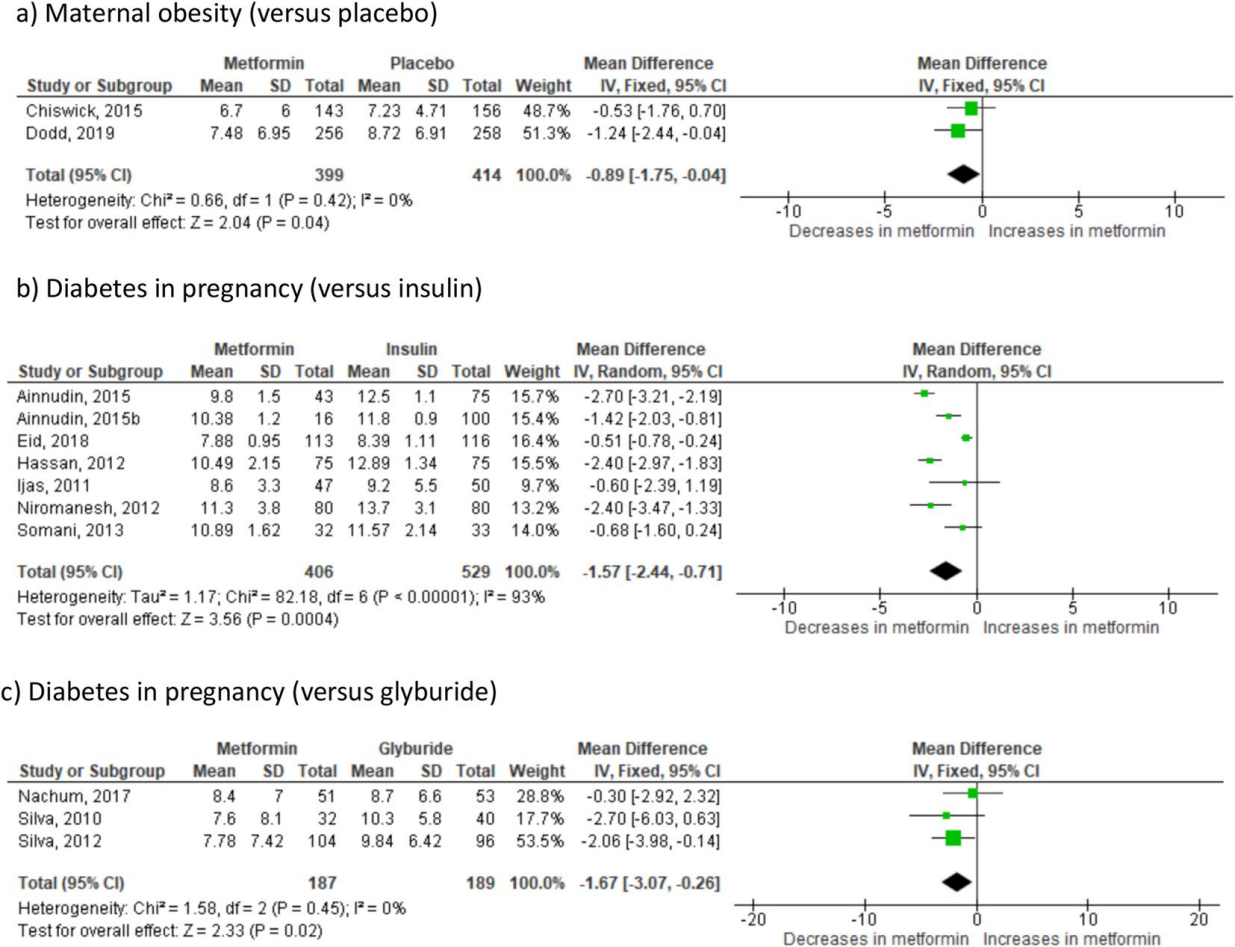
Effect of metformin randomisation upon gestational weight gain (throughout pregnancy); (all indications), based on 14 studies including 3004 pregnancies. Mean difference IV, random-effects model, 95% CI.

Where the indication for randomisation was maternal obesity, there was on average 0.89 kg less GWG (*p* = 0.04) in metformin-treated women compared to those randomised to placebo (2 studies^23,24^ n = 813) (Fig. 1a). In the group where the indication for randomisation was PCOS, there was on average 2.4 kg less GWG (95% CI 3.38–1.42 kg; I_2_ = N/A, *p* < 0.0001) in metformin-treated women compared to those randomised to placebo (1 study^25^ n = 398). In the group of women with diabetes in pregnancy, randomisation to metformin also resulted in significantly less GWG. Effect sizes were similar across all diabetes in pregnancy groups: − 1.57 kg for insulin (*p* = 0.0004) (7 studies^26–32^ n = 935) (Fig. 1b), − 1.67 kg for glyburide (*p* = 0.02) (3 studies^33–35^ n = 376) (Fig. 1c) and − 1.50 kg for placebo (95% CI − 2.39 to − 0.61, I_2_ = N/A, *p* = 0.001) (1 study^36^ n = 482). This reduction in GWG was seen consistently across in all included groups (Supplementary Fig. S7a). The 95% prediction interval for GWG across all groups was − 3.17 to 0.05 kg.

#### Pre-eclampsia

**Figure 2:**
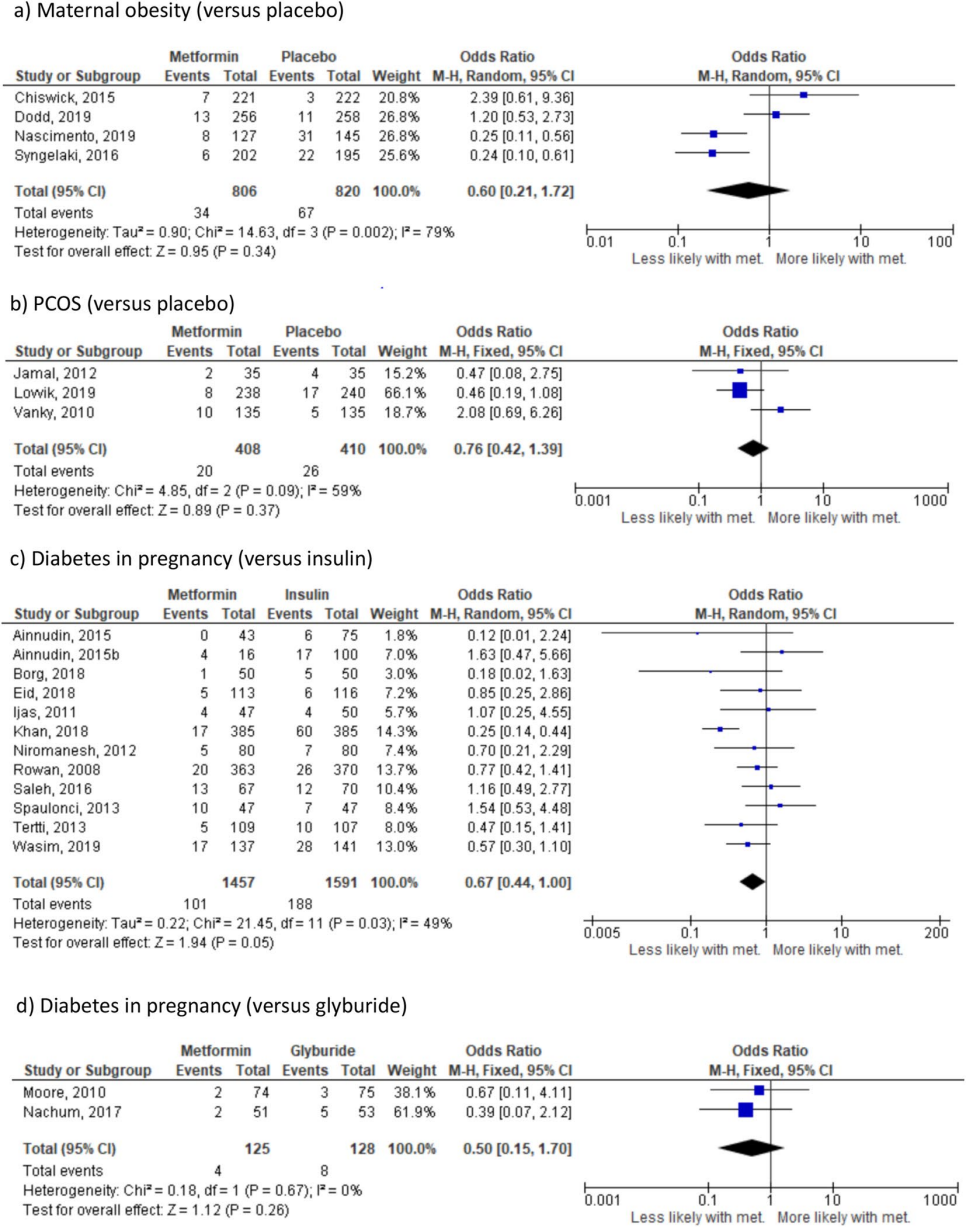
Effect of metformin randomisation upon pre-eclampsia (all indications), based on 21 studies including 5979 pregnancies. Odds ratio, random-effects model, 95% CI.

There were no significant differences in the risk of pre-eclampsia where the indication for randomisation was maternal obesity (4 studies^23,24,37,38^, n = 1620) (Fig. 2a), PCOS (3 studies^25,39,40^ n = 818) (Fig. 2b) or diabetes in pregnancy. Effect sizes were similar across all diabetes in pregnancy groups: insulin (12 studies^26–28,30,31,41–47^ n = 3048) (Fig. 2c), glyburide (2 studies^33,48^ n = 253) (Fig. 2d) and placebo (OR 1.29, 95% CI 0.77–2.16, I_2_ = N/A, *p* = 0.34) (1 study^36^ n = 482). However in all groups, there was a clear trend towards a reduction in the likelihood of pre-eclampsia in women randomised to metformin compared to any other treatment. When all indications were combined, there was a significant reduction in the likelihood of pre-eclampsia in women randomised to metformin (OR 0.69, 95% CI 0.50–0.95; I_2_ = 55%, *p* = 0.02) (Supplementary Fig. S7b); based on 23 studies including 6301 pregnancies. The 95% prediction interval was OR 0.24–1.99.

#### Gestational hypertension

**Figure 3:**
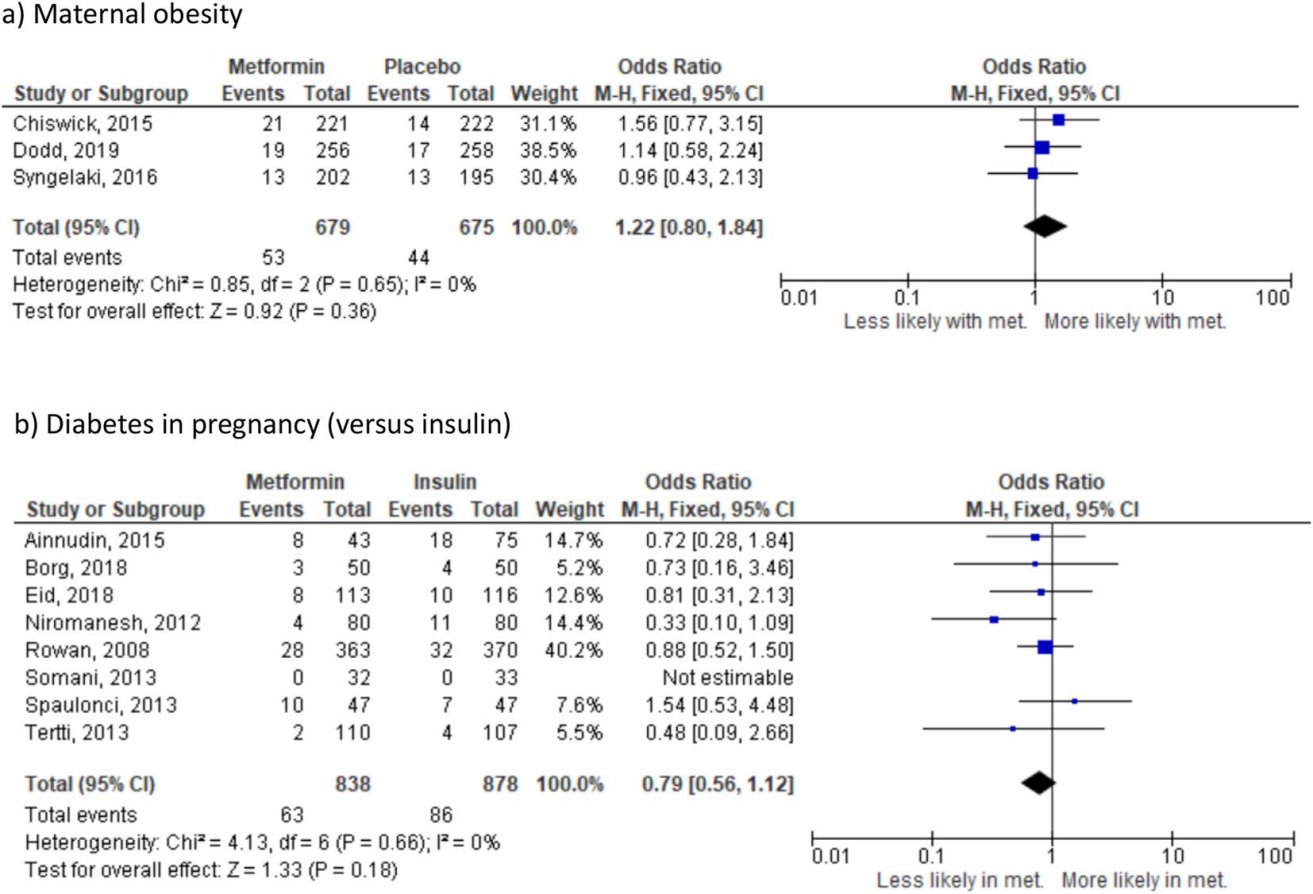
Effect of metformin randomisation upon gestational hypertension (all indications), based on 14 studies including 4189 pregnancies. Odds ratio, fixed-effects model, 95% CI.

There were no significant differences in the risk of gestational hypertension where the indication for randomisation was maternal obesity (3 studies^23,24,37^ n = 1354) (Fig. 3a), PCOS (OR 1.26, 95% CI 0.59–2.68; I_2_ = N/A, *p* = 0.55) (1 study^25^ n = 478) or diabetes in pregnancy. Effect sizes were similar across all diabetes in pregnancy groups: insulin (8 studies^26,28,31,32,41,43,45,46^, n = 1716) (Fig. 3c), glyburide (OR 1.16, 95% CI 0.42–3.17, I_2_ = N/A, *p* = 0.34) (1 study^49^, n = 159) or placebo (OR 0.87, 95% CI 0.40–1.86; I_2_ = N/A, *p* = 0.71) (1 study^36^, n = 482). In contrast to the finding with respect to pre-eclampsia, there was no difference in the likelihood of PIH between women randomised to metformin versus any other treatment when all studies were combined (OR 0.95, 95% CI 0.77–1.18; I_2_ = 0%, *p* = 0.66) (Supplementary Fig. S7c) based on 16 studies including 4189 pregnancies. The 95% prediction interval was OR 0.78–1.19.

In view of the heterogeneity of definitions that are used globally for pre-eclampsia and gestational hypertension, we also conducted an overall meta-analysis of all reported hypertensive disease of pregnancy outcomes. Women who were randomised to metformin v. any other treatment had a significantly decreased risk of any hypertensive disease of pregnancy (OR 0.76, 95% CI 0.60–0.95; I_2_ = 47%, *p* = 0.01) (Supplementary Fig. S7d); based on 23 studies including 11,145 pregnancies.

#### Preterm birth (any-cause, spontaneous and iatrogenic)

**Figure 4:**
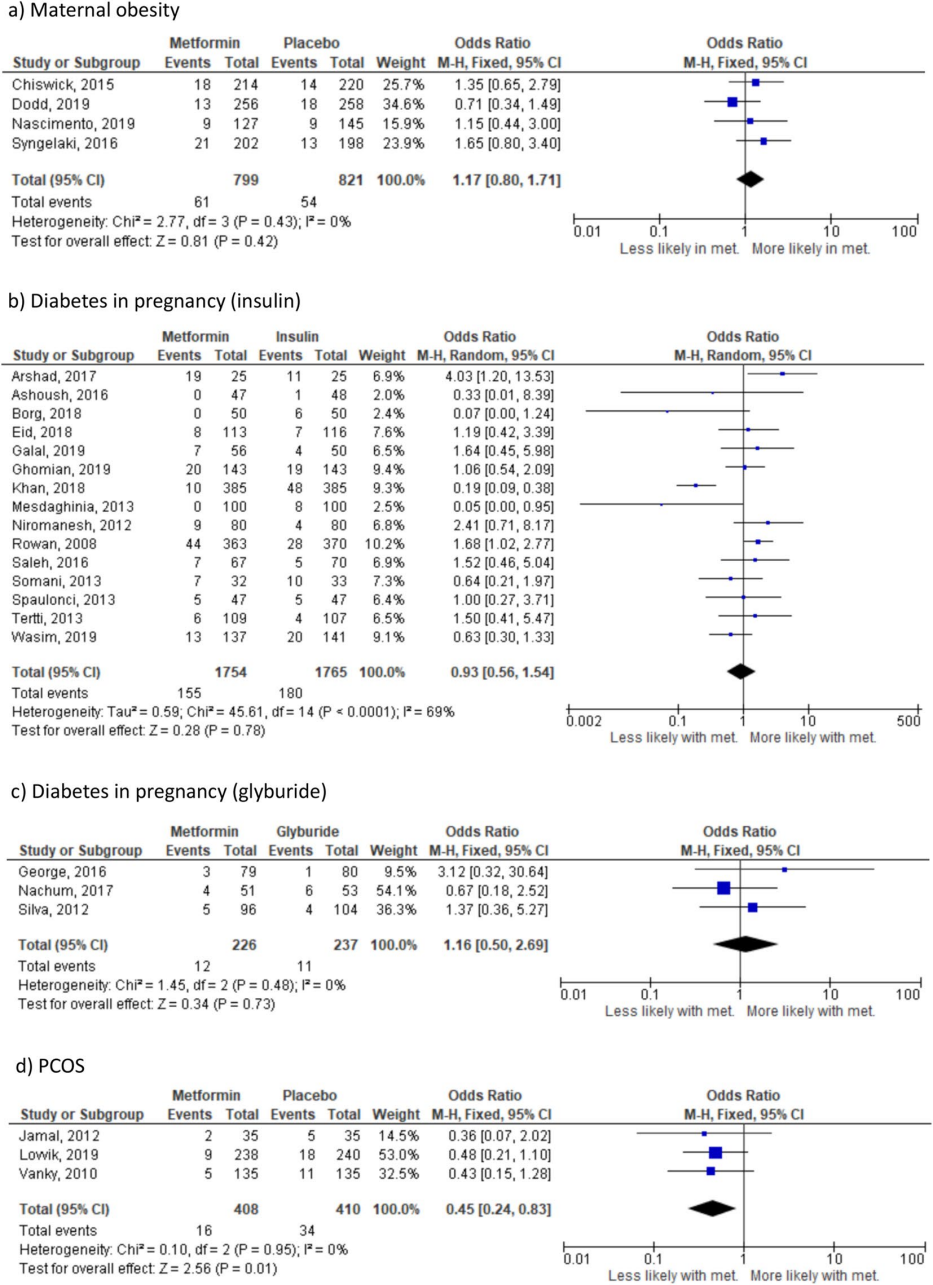
Effect of metformin randomisation upon pre-term birth (all indications). (**a**) All-cause (based on 27 studies including 6959 pregnancies), (**b**) spontaneous (based on 4 studies including 2308 pregnancies) and (**c**) iaotrogenic (based on 5 studies including 2050 pregnancies). Odds ratio, (95% CI). Fixed-effects model for (**a**) and (**b**), random-effects model for (**c**).

There were no significant differences in the risk of any-cause preterm birth in the sub-group analyses where the indication for randomisation was maternal obesity (4 studies^23,24,37,38^ n = 1620) (Fig. 4a) or diabetes in pregnancy. Effect sizes were similar across all diabetes in pregnancy groups: insulin (15 studies^28,31,32,41–47,50–54^, n = 3519) (Fig. 4b), glyburide (3 studies^33,35,49^, n = 463) (Fig. 4c) and placebo (OR 1.35, 95% CI 0.87–2.09; I_2_ = 0%, *p* = 0.18) (1 study^36^, n = 461). However, in the sub-group where the indication for randomisation was PCOS (3 studies^25,39,40^ n = 827) (Fig. 4d), randomisation to metformin was associated with reduced likelihood of preterm birth (*p* = 0.01). Insufficient studies were available to meaningfully perform planned analyses with respect to spontaneous versus iatrogenic preterm birth. There was no difference in the overall likelihood of preterm birth between women randomised to metformin versus other interventions (Supplementary Fig. S7d) based on 27 studies including 7043 pregnancies. The 95% prediction interval was OR 0.31–2.64.

#### Gestational age at delivery

**Figure 5:**
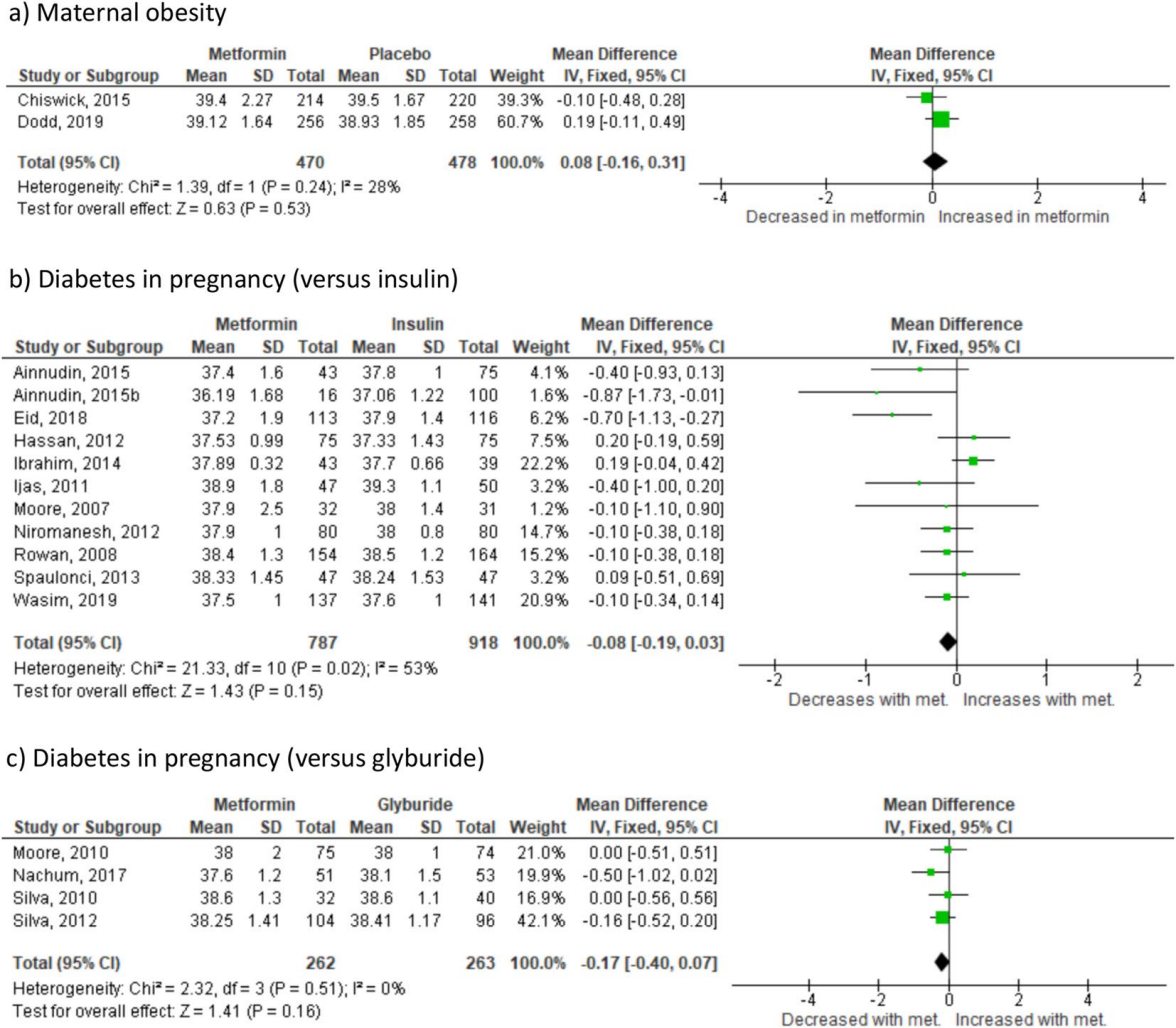
Effect of metformin randomisation upon gestational age at delivery (all indications), (based on 18 studies including 3818 pregnancies). Odds ratio (95% CI), random-effects model.

There was no significant difference in gestational age at delivery where the indication for randomisation was maternal obesity (2 studies^23,24^ n = 948) (Fig. 5a) or diabetes in pregnancy. Effect sizes were similar between all diabetes in pregnancy groups: insulin (12 studies^26–31,43,45–47^ n = 2345) (Fig. 5b), glyburide (4 studies^33–35,48^ n = 525) (Fig. 5c). No studies reported gestational age at delivery in the sub-group of women with PCOS. Randomisation to metformin versus any other treatment did not significantly influence gestational age at delivery, based on 17 studies including 3803 pregnancies (Supplementary Fig. S7e). The 95% prediction interval was − 0.48 to 0.32 weeks.

#### Caesarean section (all cause, emergency and elective)

**Figure 6:**
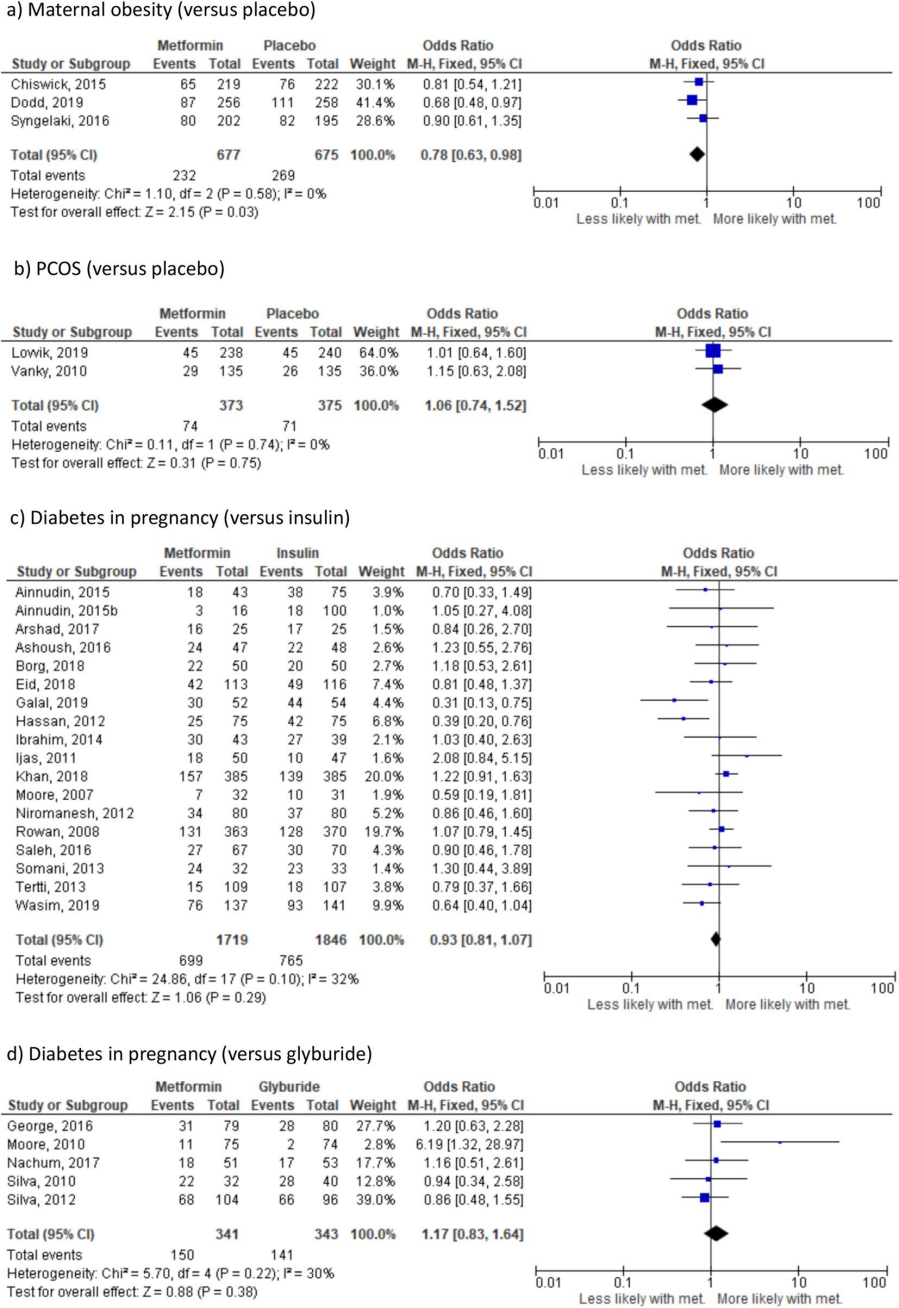
Effect of metformin randomisation upon caesarean section (all indications). (**a**) All cause, (based on 29 studies including 6122 pregnancies), (**b**) emergency (based on 7 studies including 1772 pregnancies) and (**c**) elective (based on 5 studies including 1552 pregnancies). Odds ratio (95% CI). Random-effects model for (**a**) and (**b**). Fixed-effects model for (**c**).

In the sub-group where the indication for randomisation was maternal obesity (3 studies^23,24,37^ n = 1352), randomisation to metformin (versus placebo) was associated with reduced likelihood (*p* = 0.03) of caesarean section (Fig. 6a). Where the indication for randomisation was PCOS (2 studies^25,40^ n = 1352) (Fig. 6b) or diabetes in pregnancy randomisation to metformin versus other treatments did not alter likelihood of caesarean section. Effect sizes were similar between all diabetes in pregnancy groups: insulin (17 studies^26–32,41,42,44,46,47,50–52,55,56^ n = 2872) (Fig. 6c), glyburide (5 studies^33–35,48,49^, n = 684) (Fig. 6d) and placebo (OR 0.68, 95% CI 0.47–1.00; I_2_ = N/A, *p* = 0.05) (1 study^36^, n = 470). When analysis was performed combining all indications for metformin treatment, there was a lower likelihood of delivery by caesarean section in women randomised to metformin versus other treatments (OR 0.90, 95% CI 0.82–1.00; I_2_ = 23%, *p* = 0.04) (Supplementary Fig. S7f) based on 31 studies including 7053 pregnancies. When sub-group analysis was performed separating emergency and elective caesarean section, there was no significant effect of randomisation to metformin versus other treatments on likelihood of either type of caesarean section (Supplementary Fig. S7g and S7h). The 95% prediction interval was OR 0.66–0.78.

#### Side-effects

Randomisation to metformin versus placebo for any indication during pregnancy was associated with increased likelihood of nausea, vomiting and diarrhoea, but not abdominal pain or non-gastrointestinal side-effects (Supplementary Table S7). Nausea, vomiting, and diarrhoea were all significantly increased when the indication for metformin randomisation was maternal obesity (Table 1). However, when the indication for randomisation was PCOS, fewer studies were available for analysis and only the likelihood of diarrhoea was significantly increased with metformin versus placebo (Table 1). Trials involving women with diabetes in pregnancy either did not report gastrointestinal side effects in the insulin arm, or reported zero values. Between 2 and 46% of women randomised to metformin for treatment of diabetes in pregnancy reported gastrointestinal side-effects (weighted average incidence 12.5%; Supplementary Table S8) and 0–6% of women stopped medication due to these side effects (weighted average incidence 14.3%), (Supplementary Table S8).

**Table 1:** Effect of metformin intervention upon common side effects in women with maternal obesity/PCOS.

		Effect size estimate (95% CI) or OR (95% CI)	*p* value	Studies	N	Het I_2_	Het. *p* value
Overall GI side effects	Placebo	2.43 (1.53–3.84)	0.0002	4	1441	76%	0.006
Nausea	Maternal obesity	1.43 (1.11–1.84)	0.006	3	1201	36%	0.21
	PCOS	1.60 (0.75–3.45)	0.23	1	240	N/A	N/A
Vomiting	Maternal obesity	1.41 (1.08–1.83)	0.01	3	1201	35%	0.22
	PCOS	1.85 (0.45–7.58)	0.39	1	240	N/A	N/A
Diarrhoea	Maternal obesity	2.34 (1.39–3.94)	0.001	3	1201	67%	0.05
	PCOS	6.47 (2.17–19.29)	0.0008	1	240	N/A	N/A
Abdominal pain	Maternal obesity	0.98 (0.66–1.44)	0.91	3	1201	0%	0.99
	PCOS	0.50 (0.19–1.32)	0.16	1	240	62%	0.11
Bloating	Maternal obesity	NO STUDIES	–	–	–	–	–
	PCOS	1.32 (0.73–2.38)	0.36	1	240	N/A	N/A
Constipation	Maternal obesity	1.11 (0.76–1.63)	0.59	3	797	15%	0.28
	PCOS	NO STUDIES	–	–	–	–	
Headache	Maternal obesity	1.17 (0.82–1.69)	0.39	2	797	69%	0.07
	PCOS	NO STUDIES	–	–	–	–	–
GDM	Maternal obesity	1.16 (0.68–1.96)	0.59	2	1208	68%	0.08
	PCOS	1.00 (0.71–1.42)	0.99	3	746	0%	0.54

#### GDM in participants randomised for indications other than diabetes

Randomisation to metformin versus any other treatment (placebo) did not alter the likelihood of subsequent GDM diagnosis (OR 1.07, 95% CI 0.87–1.33; I_2_ = 0%, (*p* = 0.52) (Supplementary Fig. S8), based on 7 studies including 2063 pregnancies. Whether the indication for randomisation was maternal obesity (3 studies^23,24,37^ n = 1206) or PCOS (3 studies^25,39,40^ n = 746) (Supplementary Fig. S8), randomisation to metformin did not alter likelihood of GDM.

#### Glycaemic control in diabetes in pregnancy

There was no significant difference in FBS (19 studies, n = 3673) (Supplementary Fig. S9a) or RBS (18 studies, n = 3610) (Supplementary Fig. S10a) measurements in women with diabetes in pregnancy randomised to metformin versus other treatments. Effect sizes were similar and non-significant in all diabetes in pregnancy sub-groups: FBS: insulin (14 studies^26–28,31,32,41–46,50,51,56^ n = 2945), glyburide (4 studies^33–35,48^, n = 525) (Supplementary Fig. S9b and c), and placebo (1 study^36^, n = 140) (OR − 2.40, 95% CI − 6.98 to 2.18; I_2_ = N/A, *p* = 0.30). RBS: insulin (12 studies^26–28,31,41–45,47,51,56^, n = 2895), glyburide (4 studies^33–35,48^ n = 525) (Supplementary Fig. S10b and c), and placebo (1 study^36^ n = 125), (OR-1.20, 95% CI − 6.82 to 4.42; I_2_ = N/A, *p* = 0.68). Maternal hypoglycaemia was significantly (*p* = 0.005) less likely in women randomised to metformin versus other treatments (Supplementary Fig. S11a), based on 6 studies including 1149 pregnancies^32,36,47,48,51,55^, however this effect is driven entirely by studies where insulin was the comparator group (Supplementary Fig. S11b).

## Discussion

Our results demonstrate that exposure to metformin versus other treatments during pregnancy reduced GWG, an effect consistently observed across all indications and sensitivity analyses. The likelihood of developing pre-eclampsia was reduced in women randomised to metformin in all treatment indication sub-groups. This did not reach statistical significance likely because of insufficient data when each indication for treatment was considered separately. However both the direction and the magnitude of the observed change was similar across all sub-groups of indications for metformin treatment. No difference in the incidence of gestational hypertension, gestational age at delivery or glycaemic control (irrespective of indication) was observed in any treatment indication sub-group. Randomisation to metformin in women with obesity (compared to placebo) was associated with significant reduction in the likelihood of cesarean section. Significantly reduced likelihood of preterm birth was only observed in women with PCOS randomised to metformin compared with placebo. Women randomised to metformin versus insulin for treatment of diabetes in pregnancy had a significantly lower likelihood of experiencing hypoglycaemia. However the likelihood of gastrointestinal symptoms (nausea, vomiting and diarrhoea) was significantly increased in women randomised to metformin versus other treatments. The incidence of GDM was not significantly different in metformin-treated compared to other treatment groups, hence metformin is unlikely to be of benefit as a preventative measure against GDM (where GDM was not the indication for treatment).

A major strength of this study is the breadth of outcomes affecting women during pregnancy that have been included. Our focus on maternal outcomes complements our previous work performed on fetal and childhood outcomes^12,13^. We also performed extensive sub-group and sensitivity evaluation of our conclusions.

The drawing of definitive conclusions from our meta-analysis is limited by both the quantity and quality of the studies available. In particular, there was a paucity of trials randomising women with PCOS (3 studies, n = 930 women) or maternal obesity (4 studies, n = 1485 women), limiting our confidence in conclusions relating specifically to treatment for these indications and therefore we urge a conservative view with regard to interpretation of this sub-group analyses. No randomised trials were found that compared metformin specifically with dietary/lifestyle intervention, although several studies included these interventions for both trial arms. Our search results highlight the need for more high-quality studies investigating metformin use during pregnancy. A further reason for caution in interpretation is the high heterogeneity in dose, GDM diagnosis criteria and starting point during gestation of metformin treatment between the various included studies.

Overall, metformin use during pregnancy is associated with a greater risk of experiencing gastrointestinal side-effects than placebo or other treatments. Gastrointestinal symptoms are reported in 20–30% of patients treated with metformin outside of pregnancy^57,58^. A variety of mechanisms are proposed including bile-salt malabsorption, gut serotonin secretion, and alterations to glucose and incretin metabolism^58^. These symptoms may be more common in women^59^ and more difficult to tolerate during pregnancy due to concomitant pregnancy-related nausea, vomiting, and food aversions^59,60^.

Clear evidence of benefit from randomisation to metformin observed in all sub-groups is limited to a reduction in GWG, which may be related to direct actions of metformin which can inhibit food intake, via increased concentration of growth/differentiation-factor-15 (GDF15)^61^. Excessive GWG is associated with perinatal complications including increased risk of fetal growth anomalies, risk of GDM, cesarean delivery and pre-eclampsia^36,62^. Moreover, increased GWG is associated with long-term (later in life) health risks to the mother including post-partum weight retention, obesity^63,64^ and increased risk of developing type 2 diabetes^65^ and cardiovascular disease^66^. Limiting GWG may also improve outcomes for future pregnancies^67^. The average weight gain observed in pregnancies affected by GDM is approximately 9 kg^62^ therefore a reduction of 1.55 kg (17%, as observed here) constitutes a potentially clinically significant reduction in total GWG. The observed reduction in GWG for women randomised to metformin in studies of maternal obesity was smaller (0.89 kg vs. 1.55 kg overall), which likely reflects the increased likelihood of lower GWG in obese women^68^.

The likelihood of pre-eclampsia was non-significantly reduced in women with diabetes in pregnancy randomised to metformin versus insulin. This is in keeping with other recent studies^11^, although previous findings were limited only to analysis of women with GDM. Other studies have reported a higher incidence of pre-eclampsia in women with GDM compared to those with normal glucose tolerance^69,70^; it is thus possible that any impact of metformin in reducing the likelihood of pre-eclampsia may be more readily detectable in populations with higher baseline risk. Mechanistically, it is plausible that metformin could prevent pre-eclampsia via reduction of anti-angiogenic factors, improvements of endothelial function via actions on the mitochondria and/or via actions through the mammalian target of rapamycin (mTOR) pathway by modification of cellular homeostasis and energy deposition^70^. These potential mechanisms are supported by the fact that metformin does not appear to influence the risk of gestational hypertension, which is associated only with hypertension rather than multisystem end-organ involvement^71,72^ [16230510] often attributed to wide-spread maternal endothelial and metabolic dysfunction^73^ [33189710]. Previous meta-analyses have explored the impact of metformin on pre-eclampsia risk, with mixed results^20,21,71^. Our work builds on these, with the inclusion of several more recently published studies^10,11^. At least one previous meta-analysis that included both GDM and non-GDM pregnancies and analysed both randomised and observational data^72^ found no significant effect of metformin on pre-eclampsia risk. We note the wide 95% prediction interval associated with our meta-analysis, reflecting the relatively high heterogeneity between included studies and the possibility that future studies on this topic could find significantly different results.

Our finding that metformin reduces the rate of cesarean section in obese women but not in other sub-groups may relate to our previous finding of lower birth weight associated with randomisation to metformin^15^, as there is increased likelihood of vaginal delivery with smaller fetuses. Maternal obesity is associated with increased birth weight^67^ hence the impact of metformin in reducing fetal size and thus decreasing the risk of cesarean section may be amplified in this sub-group.

In weighing the risks and benefits of metformin treatment in pregnancy to the materno-fetal dyad, our meta-analysis highlights largely neutral or positive maternal outcomes, with the notable exception of increased likelihood of gastrointestinal side effects. There was no effect of metformin in reducing the risk of developing GDM, and metformin may not be adequate pharmacological treatment for GDM in up to 46% of women^12^. From the fetal point of view however, it has previously been demonstrated that randomisation to metformin treatment for GDM is associated with increased risk of low birth-weight followed by accelerated growth in childhood^12,13^, independent of maternal glycaemic control^13^. It is particularly important to carefully consider the impacts of metformin treatment on both mother and baby as there are other suitable alternative treatments for GDM and no necessity for drug treatment for PCOS or maternal obesity in pregnancy. Moreover, there are other methods of controlling GWG, for example diet and lifestyle modification. Individual pregnant women may weigh the importance of limiting gestational weight gain or of avoiding gastrointestinal symptoms differently, and these findings may thus influence decision-making around metformin treatment in pregnancy.

## Supplementary Information

Supplementary Information.

Supplementary Table.

## Data Availability

The data for this meta-analysis are freely available. The PROSPERO protocol can be found at https://www.crd.york.ac.uk/prospero CRD ID: CRD42020167692.
