# Supplementary material for: Impact of metformin treatment during pregnancy on maternal outcomes: a systematic review/meta-analysis
Source: Sci Rep. 2021 Apr 29;11:9240. doi: 10.1038/s41598-021-88650-5 (PMC8085032; doi:10.1038/s41598-021-88650-5)
Supplement: Supplementary file 1 — Supplementary Information. [file 41598_2021_88650_MOESM1_ESM.pdf]

Supplementary Fig. S1: PRISMA flow diagram

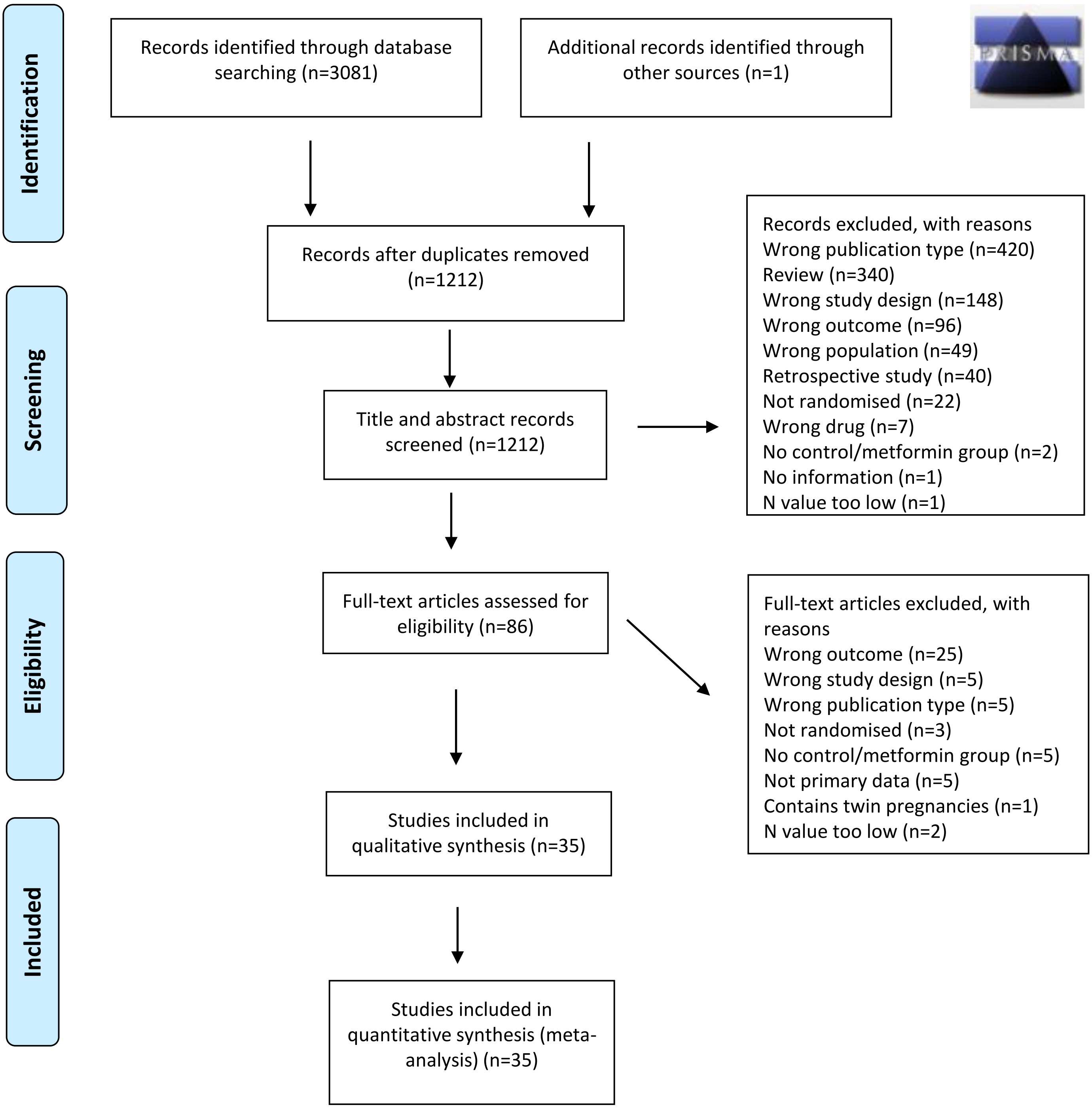

Supplementary Fig. S2: Sensitivity Analysis: Leave low-quality studies out

a) Gestational weight gain (throughout pregnancy): Metformin vs. all interventions

Study or Subgroup	Mean Difference	SE	Weight	Mean Difference IV, Fixed, 95% CI
All studies	-1.55	0.326531	46.8%	-1.55 [-2.19, -0.91]
minus Low quality studies	-1.59	0.306122	53.2%	-1.59 [-2.19, -0.99]
**Total (95% CI)**			**100.0%**	**-1.57 [-2.01, -1.13]**

Heterogeneity: Chi² = 0.01, df = 1 (P = 0.93); I² = 0%
Test for overall effect: Z = 7.04 (P < 0.00001)

Mean Difference IV, Fixed, 95% CI

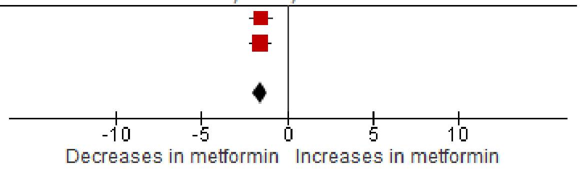

b) Gestational hypertension: Metformin vs. all interventions

Study or Subgroup	log[Odds Ratio]	SE	Weight	Odds Ratio IV, Fixed, 95% CI
All studies	0	0.127551	55.6%	1.00 [0.78, 1.28]
minus Low quality studies	0.01703334	0.142857	44.4%	1.02 [0.77, 1.35]
**Total (95% CI)**			**100.0%**	**1.01 [0.84, 1.21]**

Heterogeneity: Chi² = 0.01, df = 1 (P = 0.93); I² = 0%
Test for overall effect: Z = 0.08 (P = 0.94)

Odds Ratio IV, Fixed, 95% CI

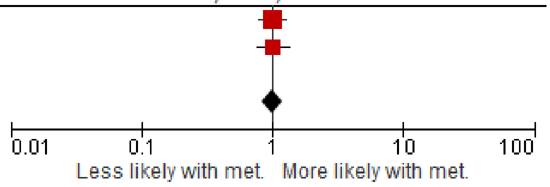

c) Preeclampsia: Metformin vs. all interventions

Study or Subgroup	log[Odds Ratio]	SE	Weight	Odds Ratio IV, Fixed, 95% CI
All studies	-0.16749109	0.117347	51.1%	0.85 [0.67, 1.06]
minus Low quality studies	-0.18045606	0.119898	48.9%	0.83 [0.66, 1.06]
**Total (95% CI)**			**100.0%**	**0.84 [0.71, 0.99]**

Heterogeneity: Chi² = 0.01, df = 1 (P = 0.94); I² = 0%
Test for overall effect: Z = 2.07 (P = 0.04)

Odds Ratio IV, Fixed, 95% CI

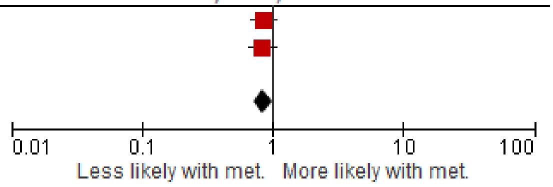

d) Gestational age at delivery: Metformin vs. all interventions

Study or Subgroup	Mean Difference	SE	Weight	Mean Difference IV, Fixed, 95% CI
All studies	-0.07	0.07398	36.5%	-0.07 [-0.21, 0.07]
minus Low quality studies	0.02	0.056122	63.5%	0.02 [-0.09, 0.13]
**Total (95% CI)**			**100.0%**	**-0.01 [-0.10, 0.07]**

Heterogeneity: Chi² = 0.94, df = 1 (P = 0.33); I² = 0%
Test for overall effect: Z = 0.29 (P = 0.77)

Mean Difference IV, Fixed, 95% CI

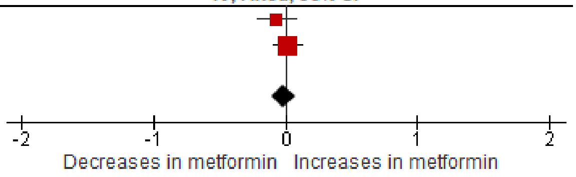

e) Preterm: Metformin vs. all interventions

Study or Subgroup	log[Odds Ratio]	SE	Weight	Odds Ratio IV, Fixed, 95% CI
All studies	-0.06048075	0.15051	52.5%	0.94 [0.70, 1.26]
minus Low quality studies	-0.0043648	0.158163	47.5%	1.00 [0.73, 1.36]
**Total (95% CI)**			**100.0%**	**0.97 [0.78, 1.20]**

Heterogeneity: Chi² = 0.07, df = 1 (P = 0.80); I² = 0%
Test for overall effect: Z = 0.31 (P = 0.76)

Odds Ratio IV, Fixed, 95% CI

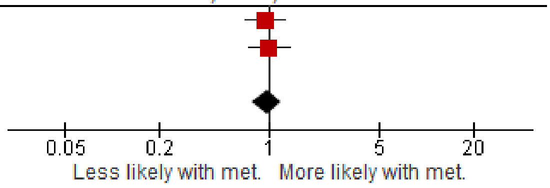

Supplementary Fig. S2: Sensitivity Analysis: Leave low-quality studies out (continued)

f) C-section rates: Metformin vs. all interventions

Study or Subgroup	log[Odds Ratio]	SE	Weight	Odds Ratio IV, Fixed, 95% CI
All studies	-0.06048	0.076531	54.8%	0.94 [0.81, 1.09]
minus Low quality studies	-0.0655	0.084184	45.2%	0.94 [0.79, 1.10]
**Total (95% CI)**			**100.0%**	**0.94 [0.84, 1.05]**

Heterogeneity: $Chi^2 = 0.00$, df = 1 (P = 0.96); $I^2 = 0\%$
Test for overall effect: Z = 1.11 (P = 0.27)

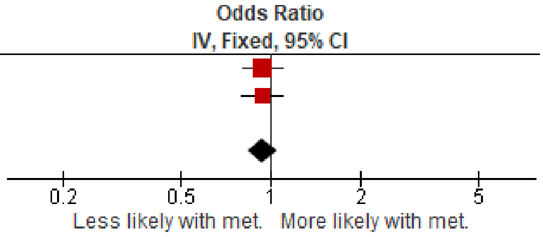

g) Development of GDM: Metformin vs. all interventions

No studies reporting this outcome were low quality

Sensitivity analysis of outcome measures when low-quality studies were removed. C-section=ceserean-section; GDM=gestational diabetes mellitus; met=metformin. Odds Ratio or mean difference (where appropriate) ± 95% CI. Fixed or random-effect models where appropriate.

Supplementary Fig. S3: Sensitivity analysis: Leave-one-out

a) Gestational weight gain: Metformin vs. all interventions

Study or Subgroup	Mean Difference	SE	Weight	Mean Difference IV, Fixed, 95% CI
All studies	-1.55	0.326531	7.6%	-1.55 [-2.19, -0.91]
minus Ainnudin, 2015	-1.42	0.306122	8.6%	-1.42 [-2.02, -0.82]
minus Ainnudin, 2015b	-1.56	0.367347	6.0%	-1.56 [-2.28, -0.84]
minus Chiswick, 2015	-1.63	0.344388	6.8%	-1.63 [-2.30, -0.96]
minus Dodd, 2019	-1.57	0.34949	6.6%	-1.57 [-2.25, -0.89]
minus Eid, 2018	-1.72	0.262755	11.7%	-1.72 [-2.23, -1.21]
minus Hassan, 2012	-1.45	0.380102	5.6%	-1.45 [-2.19, -0.71]
minus Ijas, 2011	-1.61	0.341837	6.9%	-1.61 [-2.28, -0.94]
minus Lowik, 2019	-1.47	0.380102	5.6%	-1.47 [-2.21, -0.73]
minus Nachum, 2017	-1.6	0.336735	7.1%	-1.60 [-2.26, -0.94]
minus Niromanesh, 2012	-1.47	0.341837	6.9%	-1.47 [-2.14, -0.80]
minus Silva, 2010	-1.51	0.334184	7.2%	-1.51 [-2.16, -0.86]
minus Silva, 2012	-1.52	0.339826	7.0%	-1.52 [-2.19, -0.85]
minus Somani, 2013	-1.63	0.352041	6.5%	-1.63 [-2.32, -0.94]
**Total (95% CI)**			**100.0%**	**-1.56 [-1.73, -1.38]**

Heterogeneity: Chi² = 0.94, df = 13 (P = 1.00); I² = 0%
Test for overall effect: Z = 17.36 (P < 0.00001)

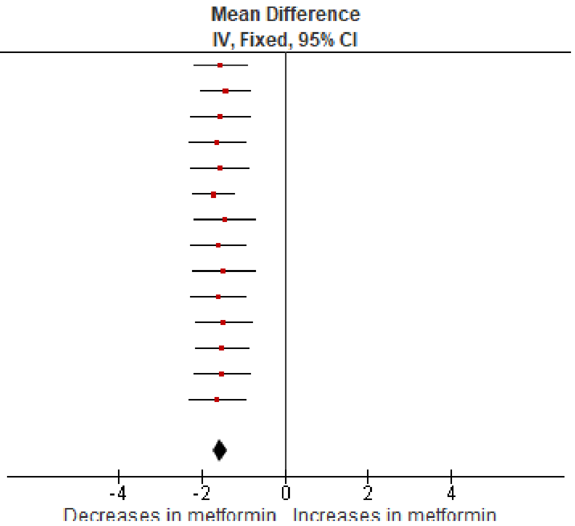

b) Pre-eclampsia: Metformin vs. all interventions

Study or Subgroup	log[Odds Ratio]	SE	Weight	Odds Ratio IV, Fixed, 95% CI
All studies	-0.18708664	0.112245	4.8%	0.83 [0.67, 1.03]
minus Ainnudin, 2015	-0.18045606	0.117347	4.4%	0.83 [0.66, 1.05]
minus Ainnudin, 2015b	-0.20760831	0.107143	5.2%	0.81 [0.66, 1.00]
minus Borg, 2018	-0.1739252	0.114796	4.5%	0.84 [0.67, 1.05]
minus Chiswick, 2015	-0.20760831	0.107143	5.2%	0.81 [0.66, 1.00]
minus Dodd, 2019	-0.20760831	0.112245	4.8%	0.81 [0.65, 1.01]
minus Eid, 2018	-0.19382003	0.117347	4.4%	0.82 [0.65, 1.04]
minus Ijas, 2011	-0.19382003	0.114796	4.5%	0.82 [0.66, 1.03]
minus Khan, 2018	-0.15490196	0.112245	4.8%	0.86 [0.69, 1.07]
minus Lowik, 2019	-0.1739252	0.122449	4.0%	0.84 [0.66, 1.07]
minus Moore, 2010	-0.18708664	0.117347	4.4%	0.83 [0.66, 1.04]
minus Nachum, 2017	-0.18045606	0.117347	4.4%	0.83 [0.66, 1.05]
minus Nascimento, 2019	-0.16115091	0.117347	4.4%	0.85 [0.68, 1.07]
minus Niromanesh, 2012	-0.18708664	0.117347	4.4%	0.83 [0.66, 1.04]
minus Rowan, 2008	-0.19382003	0.119898	4.2%	0.82 [0.65, 1.04]
minus Saleh, 2016	-0.20065945	0.112245	4.8%	0.82 [0.66, 1.02]
minus Spaulonci, 2013	-0.20760831	0.109694	5.0%	0.81 [0.66, 1.01]
minus Syngelaki, 2016	-0.16115091	0.119898	4.2%	0.85 [0.67, 1.08]
minus Tertti, 2013	-0.18045606	0.119898	4.2%	0.83 [0.66, 1.06]
minus Valdes, 2018	-0.18045606	0.117347	4.4%	0.83 [0.66, 1.05]
minus Vanky, 2010	-0.21467016	0.104592	5.5%	0.81 [0.66, 0.99]
minus Wasim, 2019	-0.18045606	0.122449	4.0%	0.83 [0.66, 1.06]
**Total (95% CI)**			**100.0%**	**0.83 [0.79, 0.87]**

Heterogeneity: Chi² = 0.45, df = 21 (P = 1.00); I² = 0%
Test for overall effect: Z = 7.68 (P < 0.00001)

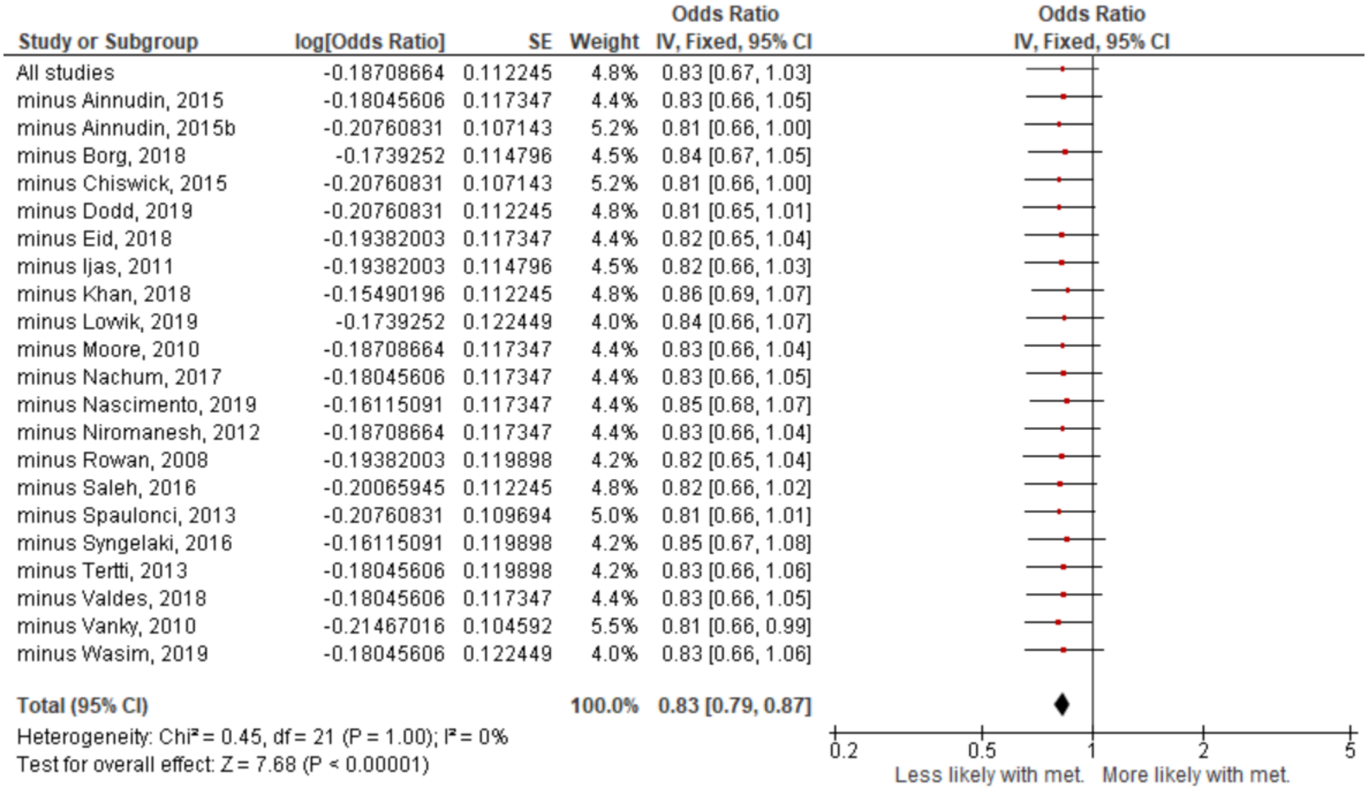

Supplementary S3 Fig: Sensitivity analysis: Leave-one-out (continued)

c) Gestational hypertension: Metformin vs. all interventions

Study or Subgroup	log[Odds Ratio]	SE	Weight	Odds Ratio IV, Fixed, 95% CI
All studies	-0.00436481	0.122449	6.3%	1.00 [0.78, 1.27]
minus Ainnudin, 2015	-0.01322827	0.114796	7.2%	0.99 [0.79, 1.24]
minus Borg, 2018	-0.00877392	0.117347	6.9%	0.99 [0.79, 1.25]
minus Chiswick, 2015	-0.03621217	0.114796	7.2%	0.96 [0.77, 1.21]
minus Dodd, 2019	-0.0222764	0.119898	6.6%	0.98 [0.77, 1.24]
minus Eid, 2018	-0.00877392	0.119898	6.6%	0.99 [0.78, 1.25]
minus Feig, 2020	-0.00877392	0.122449	6.3%	0.99 [0.78, 1.26]
minus George, 2016	-0.01772877	0.117347	6.9%	0.98 [0.78, 1.24]
minus Lowik, 2019	-0.0222764	0.119898	6.6%	0.98 [0.77, 1.24]
minus Niromanesh, 2012	0.00860017	0.122449	6.3%	1.01 [0.79, 1.28]
minus Rowan, 2008	-0.00436481	0.130102	5.6%	1.00 [0.77, 1.28]
minus Somani, 2013	-0.01322827	0.114796	7.2%	0.99 [0.79, 1.24]
minus Spaulonci, 2013	-0.0222764	0.114796	7.2%	0.98 [0.78, 1.22]
minus Syngelaki, 2016	-0.01322827	0.122449	6.3%	0.99 [0.78, 1.25]
minus Tertti, 2013	-0.00436481	0.117347	6.9%	1.00 [0.79, 1.25]
**Total (95% CI)**			**100.0%**	**0.99 [0.93, 1.05]**

Heterogeneity: Chi² = 0.11, df = 14 (P = 1.00); I² = 0%
Test for overall effect: Z = 0.43 (P = 0.67)

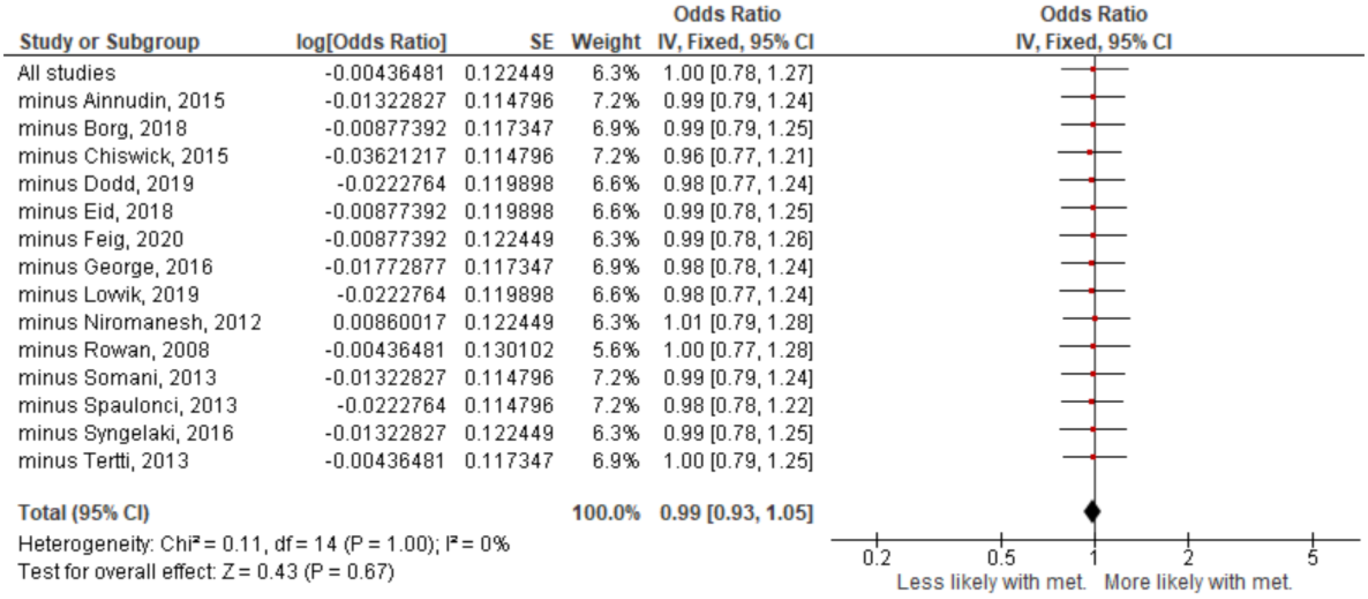

d) Preterm (all causes): Metformin vs. all interventions

Study or Subgroup	log[Odds Ratio]	SE	Weight	Odds Ratio IV, Fixed, 95% CI
All studies	-0.06048075	0.15051	4.2%	0.94 [0.70, 1.26]
minus Arshad, 2017	-0.08618615	0.140306	4.9%	0.92 [0.70, 1.21]
minus Ashoush, 2016	-0.05551733	0.155612	4.0%	0.95 [0.70, 1.28]
minus Borg, 2018	-0.05060999	0.155612	4.0%	0.95 [0.70, 1.29]
minus Chiswick, 2015	-0.07572071	0.155612	4.0%	0.93 [0.68, 1.26]
minus Dodd, 2019	-0.05551733	0.188776	2.7%	0.95 [0.65, 1.37]
minus Eid, 2018	-0.07058107	0.155612	4.0%	0.93 [0.69, 1.26]
minus Galal, 2019	-0.07058107	0.153061	4.1%	0.93 [0.69, 1.26]
minus George, 2016	-0.07058107	0.15051	4.2%	0.93 [0.69, 1.25]
minus Ghomian, 2019	-0.07058107	0.158163	3.8%	0.93 [0.68, 1.27]
minus Jamal, 2012	-0.05060999	0.158163	3.8%	0.95 [0.70, 1.30]
minus Khan, 2018	-0.00877392	0.142857	4.7%	0.99 [0.75, 1.31]
minus Lowik, 2019	-0.04575749	0.163265	3.6%	0.96 [0.69, 1.32]
minus Mesdaghinia, 2013	-0.04575749	0.155612	4.0%	0.96 [0.70, 1.30]
minus Nachum, 2017	-0.04575749	0.158163	3.8%	0.96 [0.70, 1.30]
minus Niromanesh, 2012	-0.08092191	0.147959	4.4%	0.92 [0.69, 1.23]
minus Rowan, 2008	-0.08092191	0.147959	4.4%	0.92 [0.69, 1.23]
minus Silva, 2012	-0.07058107	0.153061	4.1%	0.93 [0.69, 1.26]
minus Somani, 2013	-0.07058107	0.155612	4.0%	0.93 [0.69, 1.26]
minus Spaulonci, 2013	-0.05551733	0.160714	3.7%	0.95 [0.69, 1.30]
minus Syngelaki, 2016	-0.06550155	0.155612	4.0%	0.94 [0.69, 1.27]
minus Tertti, 2013	-0.08092191	0.15051	4.2%	0.92 [0.69, 1.24]
minus Valdes, 2018	-0.07058107	0.153061	4.1%	0.93 [0.69, 1.26]
minus Vanky, 2010	-0.05060999	0.158163	3.8%	0.95 [0.70, 1.30]
minus Wasim, 2019	-0.05060999	0.160714	3.7%	0.95 [0.69, 1.30]
**Total (95% CI)**			**100.0%**	**0.94 [0.88, 1.00]**

Heterogeneity: Chi² = 0.30, df = 24 (P = 1.00); I² = 0%
Test for overall effect: Z = 2.00 (P = 0.05)

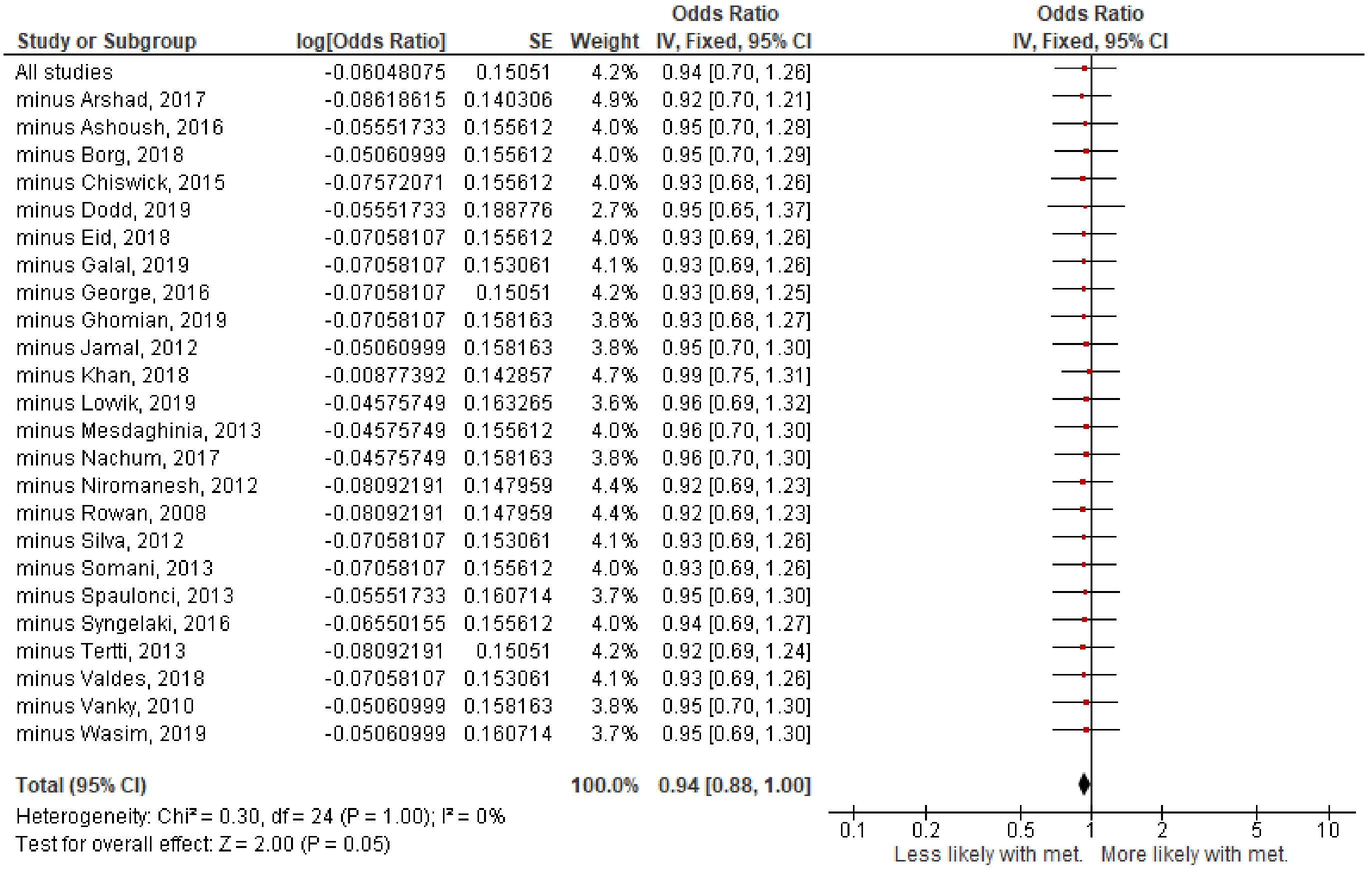

Supplementary S3 Fig: Sensitivity analysis: Leave-one-out (continued)

e) Gestational age at delivery: Metformin vs. all interventions

Study or Subgroup	Mean Difference	SE	Weight	Mean Difference IV, Fixed, 95% CI
All studies	-0.07	0.07398	6.0%	-0.07 [-0.21, 0.07]
minus Ainnudin, 2015	-0.06	0.07398	6.0%	-0.06 [-0.20, 0.08]
minus Ainnudin, 2015b	-0.05	0.071429	6.4%	-0.05 [-0.19, 0.09]
minus Chiswick, 2015	-0.07	0.079082	5.3%	-0.07 [-0.22, 0.08]
minus Dodd, 2019	-0.1	0.076531	5.6%	-0.10 [-0.25, 0.05]
minus Eid, 2018	-0.02	0.061224	8.8%	-0.02 [-0.14, 0.10]
minus Hassan, 2012	-0.09	0.076531	5.6%	-0.09 [-0.24, 0.06]
minus Ibrahim, 2014	-0.1	0.076531	5.6%	-0.10 [-0.25, 0.05]
minus Ijas, 2011	-0.1	0.071429	6.4%	-0.10 [-0.24, 0.04]
minus Moore, 2007	-0.07	0.076531	5.6%	-0.07 [-0.22, 0.08]
minus Moore, 2010	-0.08	0.076531	5.6%	-0.08 [-0.23, 0.07]
minus Nachum, 2017	-0.05	0.071429	6.4%	-0.05 [-0.19, 0.09]
minus Niromanesh, 2012	-0.07	0.079082	5.3%	-0.07 [-0.22, 0.08]
minus Silva, 2010	-0.08	0.076531	5.6%	-0.08 [-0.23, 0.07]
minus Silva, 2012	-0.07	0.079082	5.3%	-0.07 [-0.22, 0.08]
minus Spaulonci, 2013	-0.08	0.076531	5.6%	-0.08 [-0.23, 0.07]
minus Wasim, 2020	-0.07	0.081633	4.9%	-0.07 [-0.23, 0.09]
**Total (95% CI)**			**100.0%**	**-0.07 [-0.11, -0.04]**

Heterogeneity: Chi² = 1.44, df = 16 (P = 1.00); I² = 0%
Test for overall effect: Z = 3.89 (P < 0.0001)

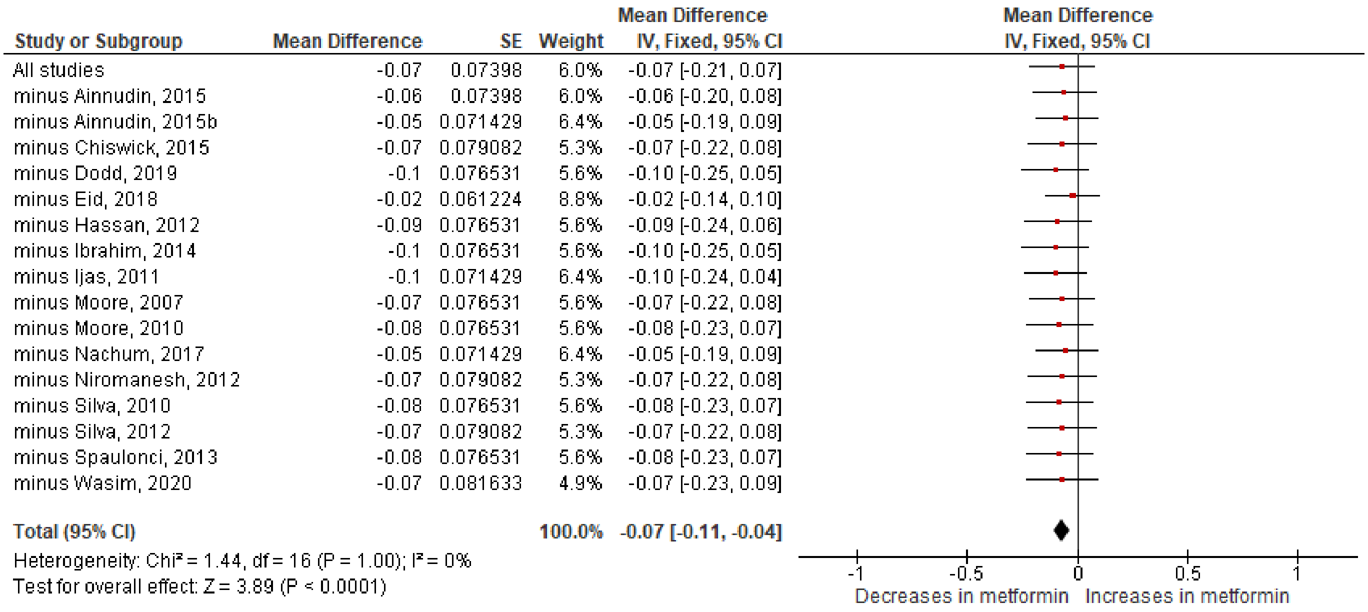

f) C-section rates: Metformin vs. all interventions

Study or Subgroup	log[Odds Ratio]	SE	Weight	Odds Ratio IV, Fixed, 95% CI
All studies	-0.06048075	0.079082	3.3%	0.94 [0.81, 1.10]
minus Ainnudin, 2015	-0.05551733	0.079082	3.3%	0.95 [0.81, 1.10]
minus Ainnudin, 2015b	-0.06048075	0.079082	3.3%	0.94 [0.81, 1.10]
minus Arshad, 2017	-0.05551733	0.076531	3.6%	0.95 [0.81, 1.10]
minus Ashoush, 2016	-0.06048075	0.076531	3.6%	0.94 [0.81, 1.09]
minus Borg, 2018	-0.06048075	0.076531	3.6%	0.94 [0.81, 1.09]
minus Chiswick, 2015	-0.05551733	0.081633	3.1%	0.95 [0.81, 1.11]
minus Dodd, 2019	-0.03621217	0.066327	4.8%	0.96 [0.85, 1.10]
minus Eid, 2018	-0.05551733	0.084184	3.0%	0.95 [0.80, 1.12]
minus Galal, 2019	-0.04575749	0.076531	3.6%	0.96 [0.82, 1.11]
minus George, 2016	-0.06550155	0.079082	3.3%	0.94 [0.80, 1.09]
minus Hassan, 2012	-0.04575749	0.076531	3.6%	0.96 [0.82, 1.11]
minus Ibrahim, 2014	-0.06048075	0.079082	3.3%	0.94 [0.81, 1.10]
minus Ijas, 2011	-0.07058107	0.07398	3.8%	0.93 [0.81, 1.08]
minus Khan, 2018	-0.07058107	0.07398	3.8%	0.93 [0.81, 1.08]
minus Lowik, 2019	-0.06048075	0.081633	3.1%	0.94 [0.80, 1.10]
minus Moore, 2007	-0.05551733	0.084184	3.0%	0.95 [0.80, 1.12]
minus Moore, 2010	-0.07058107	0.068878	4.4%	0.93 [0.81, 1.07]
minus Nachum, 2017	-0.06048075	0.076531	3.6%	0.94 [0.81, 1.09]
minus Niromanesh, 2012	-0.05551733	0.081633	3.1%	0.95 [0.81, 1.11]
minus Saleh, 2016	-0.05551733	0.081633	3.1%	0.95 [0.81, 1.11]
minus Silva, 2010	-0.06048075	0.079082	3.3%	0.94 [0.81, 1.10]
minus Silva, 2012	-0.05551733	0.081633	3.1%	0.95 [0.81, 1.11]
minus Somani, 2013	-0.06048075	0.076531	3.6%	0.94 [0.81, 1.09]
minus Syngelaki, 2016	-0.06048075	0.081633	3.1%	0.94 [0.80, 1.10]
minus Tertti, 2013	-0.05551733	0.079082	3.3%	0.95 [0.81, 1.10]
minus Valdes, 2018	-0.08092191	0.076531	3.6%	0.92 [0.79, 1.07]
minus Vanky, 2010	-0.06550155	0.079082	3.3%	0.94 [0.80, 1.09]
minus Wasim, 2019	-0.05060999	0.081633	3.1%	0.95 [0.81, 1.12]
**Total (95% CI)**			**100.0%**	**0.94 [0.92, 0.97]**

Heterogeneity: Chi² = 0.38, df = 28 (P = 1.00); I² = 0%
Test for overall effect: Z = 4.07 (P < 0.0001)

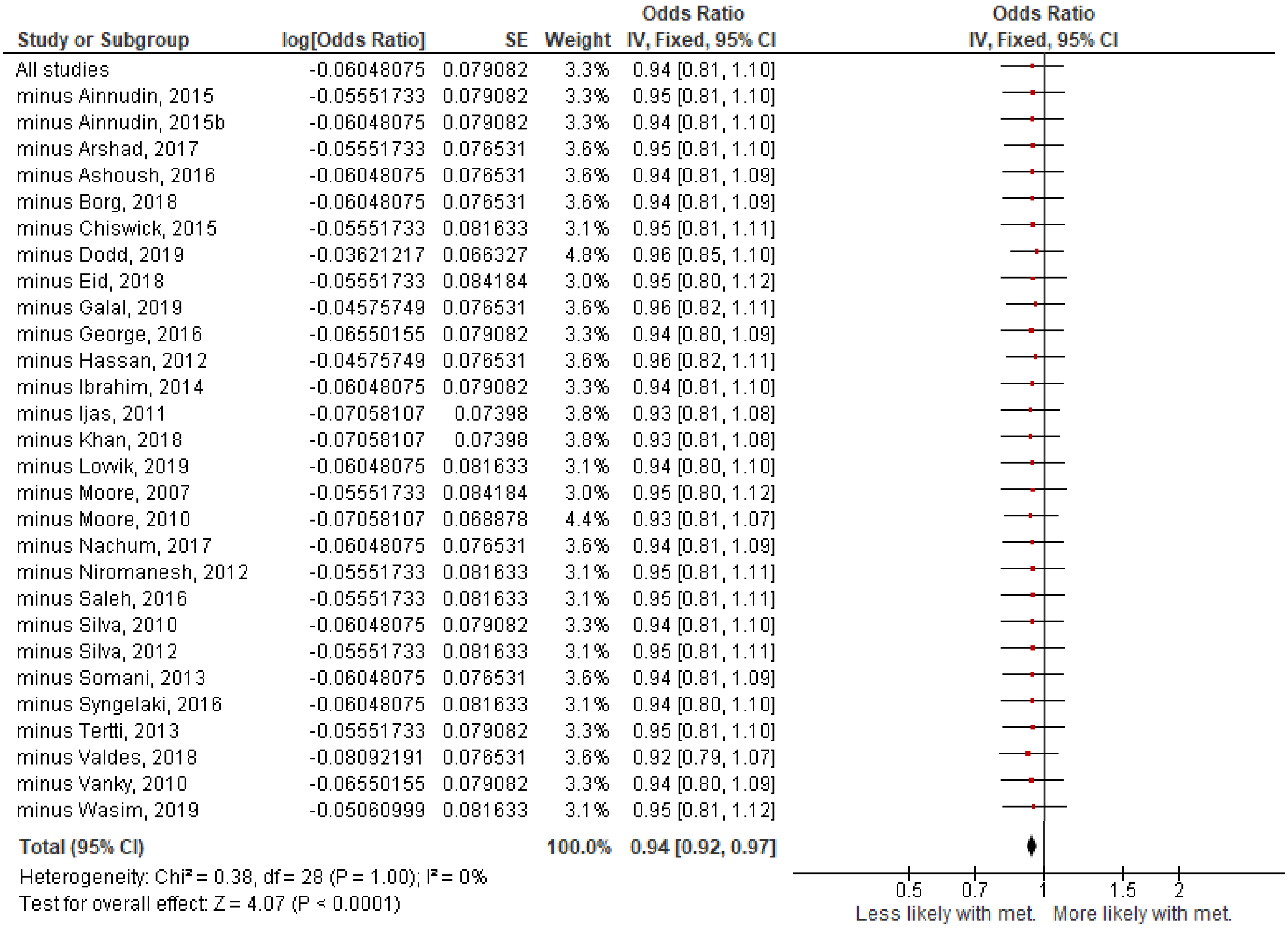

Supplementary S3 Fig: Sensitivity analysis: Leave-one-out (continued)

g) Development of GDM: Metformin vs. all interventions

Study or Subgroup	log[Odds Ratio]	SE	Weight	Odds Ratio IV, Fixed, 95% CI
All studies	0.02938378	0.117347	14.3%	1.03 [0.82, 1.30]
minus Chiswick, 2015	0.05690485	0.135204	10.8%	1.06 [0.81, 1.38]
minus Dodd, 2019	0.00432137	0.132653	11.2%	1.00 [0.77, 1.30]
minus Jamal, 2012	0.0374265	0.122449	13.2%	1.04 [0.82, 1.32]
minus Lowik, 2019	0.02938378	0.137755	10.4%	1.03 [0.79, 1.35]
minus Syngelaki, 2016	0.02938378	0.125	12.6%	1.03 [0.81, 1.32]
minus Valdes, 2018	0.01283722	0.117347	14.3%	1.01 [0.80, 1.27]
minus Vanky, 2010	0.03342376	0.122449	13.2%	1.03 [0.81, 1.31]
**Total (95% CI)**			**100.0%**	**1.03 [0.94, 1.12]**

Heterogeneity: Chi² = 0.10, df = 7 (P = 1.00); I² = 0%
Test for overall effect: Z = 0.65 (P = 0.52)

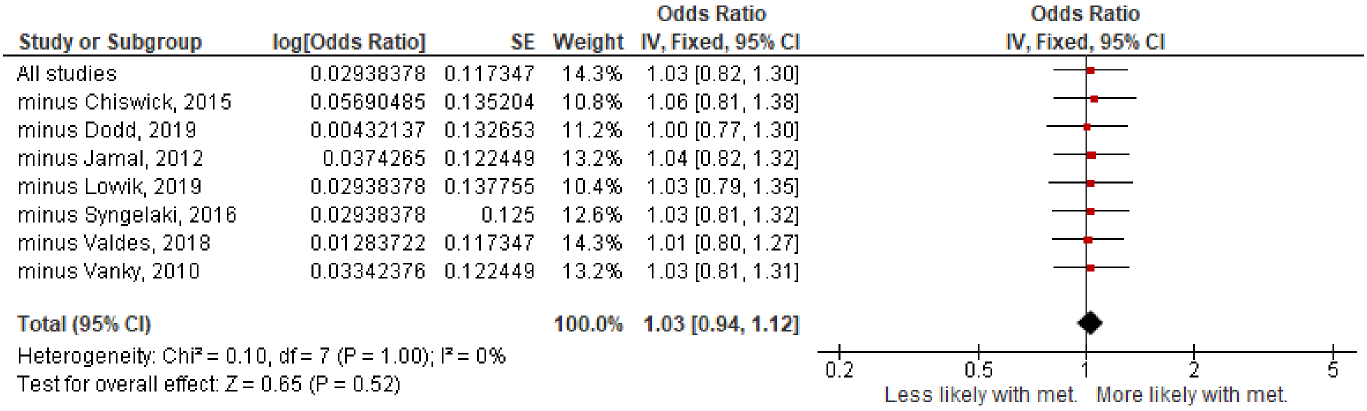

Sensitivity analysis of outcome measures one study was removed.
C-section=ceserean-section; GDM=gestational diabetes mellitus; met=metformin.
Odds Ratio or mean difference (where appropriate) ± 95% CI. Fixed or random-effect models where appropriate.

Supplementary Fig. S4: Funnel Plots

a) Gestational weight gain (throughout pregnancy)

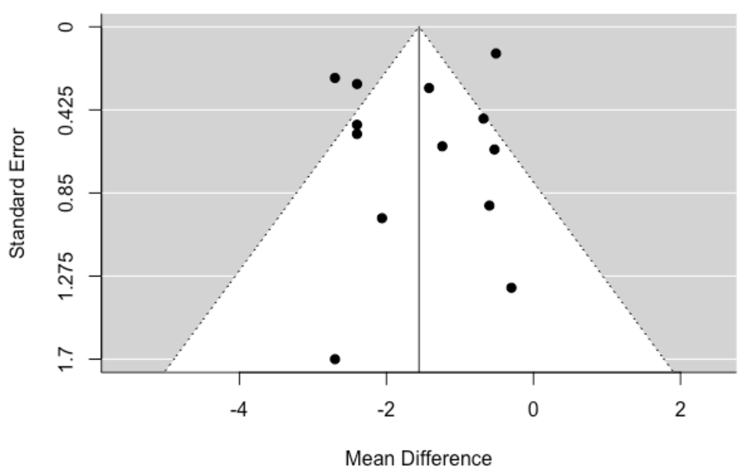

b) Gestational hypertension

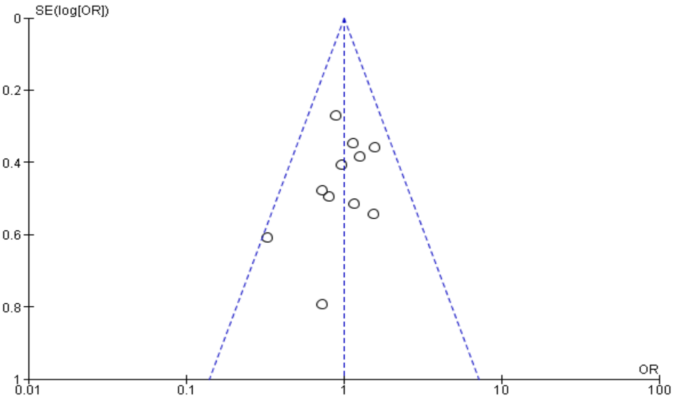

c) Pre-eclampsia

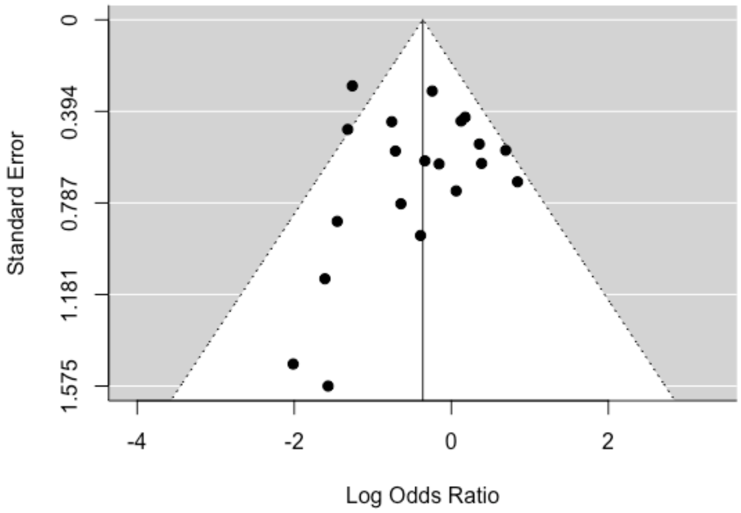

d) Gestational age at delivery

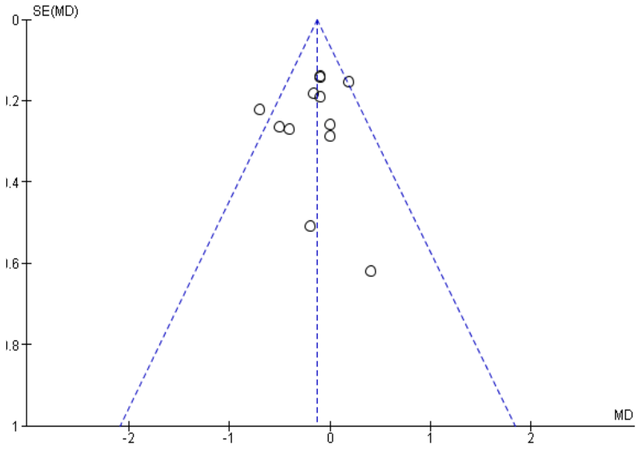

e) Preterm (all causes)

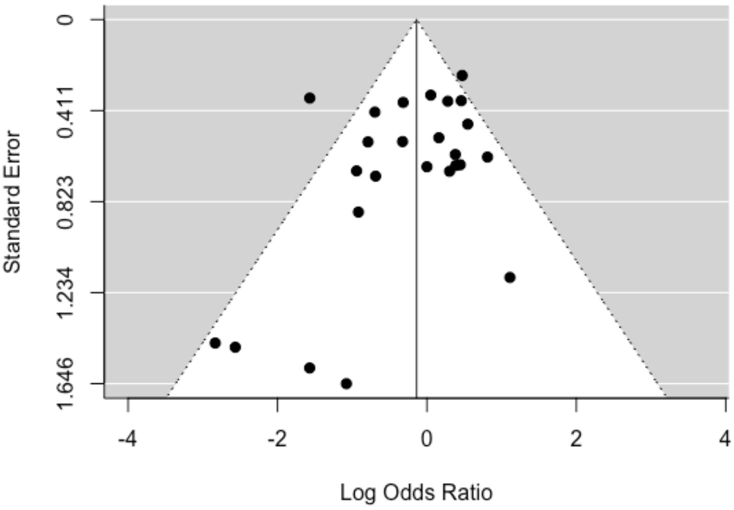

f) Preterm (spontaneous)

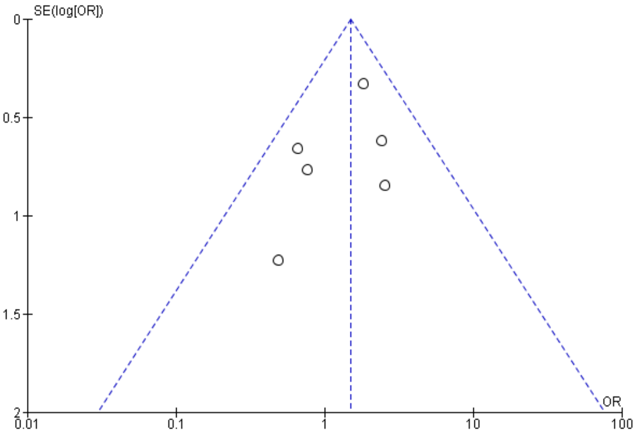

Supplementary Fig. S4: Funnel Plots (continued)

g) Ceserean section rate

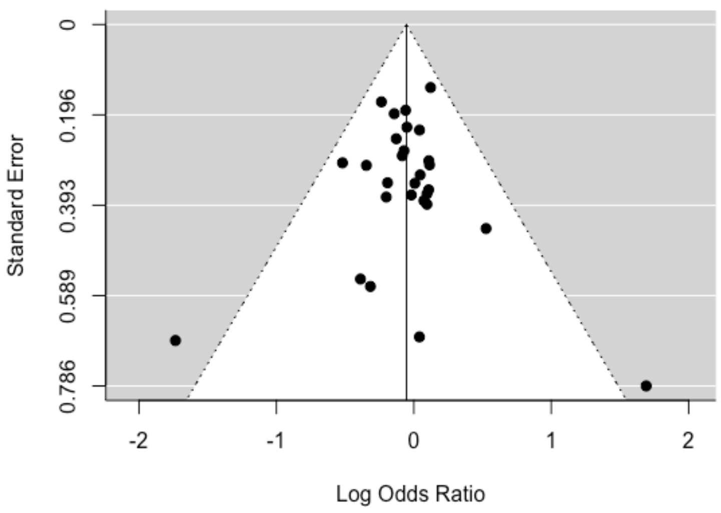

h) Incidence of GDM

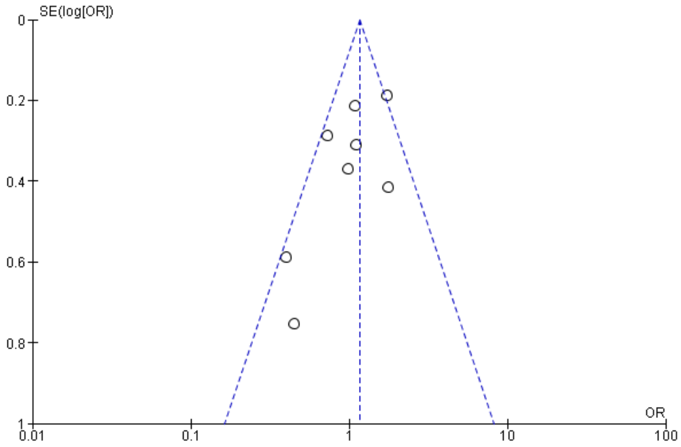

Funnel Plot analysis
C-section=ceserean-section; GDM=gestational diabetes mellitus; met=metformin.
Odds Ratio or mean difference (where appropriate) ± 95% CI. Fixed or random-effect models where appropriate.

Supplementary Fig S5: GRADE analysis for all primary outcomes

Outcomes	Plain language statements	Absolute Effect: With any comparator	Absolute Effect: With Metformin	Absolute Effect: Difference		Certainty of the evidence (GRADE)
Gestational hypertension (all studies)<br>Follow-up: 0	*(1) Risk of Bias: Studies in this outcome were deemed to have a low risk of bias, with only 1 out of 14 studies not-intention-to-treat. (Removal of this study did not alter this outcome). (2) Imprecision: 4 studies out of 14 in this outcome had large 95% confidence intervals, therefore reducing the precision of this outcome. (3) Inconsistency: Studies in this outcome were deemed to have a very high level of heterogeneity as evidenced by the I2 value of 87%, with accompanying p value of <0.00001. (4) Indirectness: Participants and interventions in all studies in this outcome did not differ from those of interest. This outcome was also specified in the PROSPERO protocol. (5) Publication bias: All studies for this outcome were judged to have a low publication bias, due no obvious asymmetries in Funnels plots for any study outcomes and Eggers Testing.*	74 per 1000	71 per 1000	Difference: 3 fewer per 1000 patients<br>(95% CI: 16 fewer to 12 more per 1000 patients)<br>Based on data from 5157 patients in 16 studies		⊕⊕⊕◯ MODERATE<br>Due to serious imprecision.
Gestational age at delivery (all studies)<br>Follow-up: 0	*(1) Risk of Bias: Studies in this outcome were deemed to have a low risk of bias, with 2 out of 17 studies not-intention-to-treat. Removal of these studies did not alter the outcome. (2) Imprecision: This outcome was judged to have a low level of imprecision due to the high number of studies (17) and participants (3081). The majority (2) had modest 95% confidence intervals. (3) Inconsistency: Studies in this outcome were deemed to have a modestly high level of heterogeneity as evidenced by the I2 value of 47%, with accompanying p value of 0.02. (4) Indirectness: Participants and interventions in all studies in this outcome did not differ from those of interest. This outcome was also specified in the PROSPERO protocol. (5) Publication bias: All studies for this outcome were judged to have a low publication bias, due no obvious asymmetries in Funnels plots for any study outcomes and Eggers Testing.*	38.2 Days	38.13 Days	Average difference (MD): 0.07 Days lower<br>(95% CI: 0.21 Days lower to 0.06 Days higher<br>Based on data from 3081 patients in 17 studies	0.07 from -0.21 to 0.06	⊕⊕⊕◯ MODERATE<br>Due to serious inconsistency.
GWG (through pregnancy) (all studies)<br>Follow-up: 0	*(1) Risk of Bias: Studies in this outcome were deemed to have a low risk of bias with 2 studies out of 13 not intention-to-treat. Removal of these studies did not alter the outcome. (2) Imprecision: This outcome was judged to have a low level of imprecision due to the moderately high number of studies (13) and participants (2522). The majority (11) had modest 95% confidence intervals. (3) Inconsistency: Studies in this outcome were deemed to have a very high level of heterogeneity as evidenced by the I2 value of 87%, with accompanying p value of <0.00001. (4) Indirectness: Participants and interventions in all studies in this outcome did not differ from those of interest. This outcome was also specified in the PROSPERO protocol. (5) Publication bias: All studies for this outcome were judged to have a low publication bias, due no obvious asymmetries in Funnels plots for any study outcomes and Eggers Testing. • Large magnitude of effect: This outcome considered to have a large magnitude of effect as evidenced by an overall effect of p<0.00001. (-1.59kg 95%CI -2.19 to -0.99).*	10.5 kg	8.95 kg	Average difference (MD): 1.55 kg lower<br>(95% CI: 2.19 to 0.91 kg lower<br>Based on data from 2522 patients in 13 studies	1.55 from -2.19 to -0.91	⊕⊕⊕⊕ HIGH<br>Due to serious inconsistency.<br>Upgraded due to large magnitude of effect.
Preterm: All causes (all studies)	*(1) Risk of Bias: Studies in this outcome were deemed to have a low risk of bias with 2 out of 26 studies not intention-to-treat. Removal of these studies did not alter this outcome. (2) Imprecision: This outcome was judged to have a low level of imprecision due to the high number of studies (26) and participants (6771). The majority (24) had modest 95% confidence intervals. (3) Inconsistency: Studies in this outcome were deemed to have a very high level of heterogeneity as evidenced by the I2 value of 59%, with accompanying p value of <0.0001. (4) Indirectness: Participants and interventions in all studies in this outcome did not differ from those of interest. This outcome was also specified in the PROSPERO protocol. (5) Publication bias: All studies for this outcome were judged to have a low publication bias, due no obvious asymmetries in Funnels plots for any study outcomes and Eggers Testing.*	96 per 1000	88 per 1000	Difference: 8 fewer per 1000 patients<br>(95% CI: 29 fewer to 19 more per 1000 patients)<br>Based on data from 6959 patients in 27 studies	8 fewer per 1000 patients	⊕⊕⊕◯ MODERATE<br>Due to serious inconsistency.
Pre-eclampsia (all studies)<br>Follow-up: 0	*(1) Risk of Bias: Studies in this outcome were deemed to have a low risk of bias with 3 studies out of 23 not intention-to-treat. Removal of these studies did not alter this outcome. (2) Imprecision: This outcome was judged to have a low level of imprecision due to the high number of studies (23) and participants (6301). The majority (21) had small 95% confidence intervals. (3) Inconsistency: Studies in this outcome were deemed to have a very high level of heterogeneity as evidenced by the I2 value of 55%, with accompanying p value of 0.0009. (4) Indirectness: Participants and interventions in all studies in this outcome did not differ from those of interest. This outcome was also specified in the PROSPERO protocol. (5) Publication bias: All studies for this outcome were judged to have a low publication bias, due no obvious asymmetries in Funnels plots for any study outcomes and Eggers Testing.*	99 per 1000	70 per 1000	Difference: 29 fewer per 1000 patients<br>(95% CI: 47 to 4 fewer per 1000 patients)<br>Based on data from 6301 patients in 23 studies	29 fewer per 1000 patients	⊕⊕⊕◯ MODERATE<br>Due to serious inconsistency.

Supplementary Fig S6: GRADE analysis for secondary outcomes

Outcomes	Plain language statements	Absolute Effect With any comparator	Absolute Effect With Metformin	Absolute Effect	Relative effect (95% CI)	Certainty of the evidence GRADE
C-section (all reasons)	(1) Risk of Bias: Studies in this outcome were deemed to have a low risk of bias, with 2 studies out of 31 studies being non intention-to-treat. (Removal of these studies did not alter this outcome). (2) Imprecision: This outcome was judged to have a low level of imprecision due to the high number of studies (31) and participants (7035). The majority (30) had small 95% confidence intervals. (3) Inconsistency: Studies in this outcome were deemed to have a low level of heterogeneity as evidenced by the I2 value of 23%, with accompanying p value of 0.12. (4) Indirectness: Participants and interventions in all studies in this outcome did not differ from those of interest. This outcome was also specified in the PROSPERO protocol. (5) Publication bias: All studies for this outcome were judged to have a low publication bias, due no obvious asymmetries in Funnels plots for any study outcomes and Eggers Testing.	406 per 1000	381 per 1000	Difference: 25 fewer per 1000 patients (95% CI: 47 to 0 fewer per 1000 patients) Based on data from 7053 patients in 31 studies	OR 0.9 (0.82 to 1)	⊕⊕⊕⊕ HIGH
Development of GDM Follow-up: 0	(1) Risk of Bias: Studies in this outcome were deemed to have a low risk of bias, with no studies out of 7 studies being non intention-to-treat. (2) Imprecision: This outcome was judged to have a high level of imprecision due to the moderately low number of studies (7) and participants (2093), 4 of which had large confidence intervals. (3) Inconsistency: Studies in this outcome were deemed to have a low level of heterogeneity as evidenced by the I2 value of 0%, with accompanying p value of 0.70. (4) Indirectness: Participants and interventions in all studies in this outcome did not differ from those of interest. This outcome was also specified in the PROSPERO protocol. (5) Publication bias: All studies for this outcome were judged to have a low publication bias, due no obvious asymmetries in Funnels plots for any study outcomes and Eggers Testing.	208 per 1000	219 per 1000	Difference: 11 more per 1000 patients (95% CI: 22 fewer to 51 more per 1000 patients) Based on data from 2063 patients in 7 studies	OR 1.07 (0.87 to 1.33)	⊕⊕⊕◯ MODERATE Due to serious imprecision.
Glycaemic control: FBS	(1) Risk of Bias: Studies in this outcome were deemed to have a low risk of bias, with 3 studies out of 18 studies being non intention-to-treat. (Removal of these studies did not alter this outcome). (2) Imprecision: This outcome was judged to have a low level of imprecision due to the high number of studies (18) and participants (3794), only 1 of which had large confidence intervals. (3) Inconsistency: Studies in this outcome were deemed to have a very high level of heterogeneity as evidenced by the I2 value of 93%, with accompanying p value of <0.00001. (4) Indirectness: Participants and interventions in all studies in this outcome did not differ from those of interest. This outcome was also specified in the PROSPERO protocol. (5) Publication bias: All studies for this outcome were judged to have a low publication bias, due no obvious asymmetries in Funnels plots for any study outcomes and Eggers Testing.	90.23 mg/dL	89.78 mg/dL	Average difference (MD): 0.45 mg/dL lower (95% CI: 2.26 mg/dL lower to 1.36 mg/dL higher Based on data from 3673 patients in 19 studies	-	⊕⊕⊕◯ MODERATE Due to serious inconsistency.
Glycaemic control: RBS Follow-up: 0	(1) Risk of Bias: Studies in this outcome were deemed to have a low risk of bias, with 3 studies out of 17 studies being non intention-to-treat. (Removal of these studies did not alter this outcome). (2) Imprecision: This outcome was judged to have a high level of imprecision due to the moderately low number of studies (17) and participants (3710), 1 of which had large confidence intervals. (3) Inconsistency: Studies in this outcome were deemed to have a high level of heterogeneity as evidenced by the I2 value of 84%, with accompanying p value of <0.00001. (4) Indirectness: Participants and interventions in all studies in this outcome did not differ from those of interest. This outcome was also specified in the PROSPERO protocol. (5) Publication bias: All studies for this outcome were judged to have a low publication bias, due no obvious asymmetries in Funnels plots for any study outcomes and Eggers Testing.	115.47 mg/dL	114.29 mg/dL	Average difference (MD): 1.18 mg/dL lower (95% CI: 2.64 mg/dL lower to 0.28 mg/dL higher Based on data from 3610 patients in 18 studies	-	⊕⊕⊕◯ MODERATE Due to serious inconsistency.
Maternal hypoglycaemia Follow-up: 0	(1) Risk of Bias: Studies in this outcome were deemed to have a low risk of bias, with 3 studies out of 17 studies being non intention-to-treat. (Removal of these studies did not alter this outcome). (2) Imprecision: This outcome was judged to have a high level of imprecision due to the low number of studies (5) and participants (679), 4 of which had large confidence intervals. (3) Inconsistency: Studies in this outcome were deemed to have a low level of heterogeneity as evidenced by the I2 value of 0%, with accompanying p value of 0.65. (4) Indirectness: Participants and interventions in all studies in this outcome did not differ from those of interest. This outcome was also specified in the PROSPERO protocol. (5) Publication bias: All studies for this outcome were judged to have a low publication bias, due no obvious asymmetries in Funnels plots for any study outcomes and Eggers Testing.	78 per 1000	38 per 1000	Difference: 40 fewer per 1000 patients (95% CI: 55 to 15 fewer per 1000 patients) Based on data from 1149 patients in 6 studies	OR 0.47 (0.28 to 0.8)	⊕⊕⊕◯ MODERATE Due to serious imprecision.

Supplementary Fig S7: All combined groups

a) GWG

Study or Subgroup	Metformin Mean	Metformin SD	Metformin Total	Placebo/insulin/glyburide Mean	Placebo/insulin/glyburide SD	Placebo/insulin/glyburide Total	Weight	Mean Difference IV, Random, 95% CI
Ainnudin, 2015	9.8	1.5	43	12.5	1.1	75	9.4%	-2.70 [-3.21, -2.19]
Ainnudin, 2015b	10.38	1.2	16	11.8	0.9	100	9.1%	-1.42 [-2.03, -0.81]
Chiswick, 2015	6.7	6	143	7.23	4.71	156	7.1%	-0.53 [-1.76, 0.70]
Dodd, 2019	7.48	6.95	256	8.72	6.91	258	7.2%	-1.24 [-2.44, -0.04]
Eid, 2018	7.88	0.95	113	8.39	1.11	116	9.9%	-0.51 [-0.78, -0.24]
Feig, 2020	7.5	5.3	240	9	4.7	242	8.2%	-1.50 [-2.39, -0.61]
Hassan, 2012	10.49	2.15	75	12.89	1.34	75	9.2%	-2.40 [-2.97, -1.83]
Ijas, 2011	8.6	3.3	47	9.2	5.5	50	5.3%	-0.60 [-2.39, 1.19]
Lowik, 2019	9.1	5.1	198	11.5	4.9	200	7.9%	-2.40 [-3.38, -1.42]
Nachum, 2017	8.4	7	51	8.7	6.6	53	3.4%	-0.30 [-2.92, 2.32]
Niromanesh, 2012	11.3	3.8	80	13.7	3.1	80	7.6%	-2.40 [-3.47, -1.33]
Silva, 2010	7.6	8.1	32	10.3	5.3	40	2.5%	-2.70 [-5.95, 0.55]
Silva, 2012	7.78	7.42	104	9.84	6.42	96	4.9%	-2.06 [-3.98, -0.14]
Somani, 2013	10.89	1.62	32	11.57	2.14	33	8.1%	-0.68 [-1.60, 0.24]
**Total (95% CI)**			**1430**			**1574**	**100.0%**	**-1.55 [-2.14, -0.95]**

Heterogeneity: Tau² = 0.91; Chi² = 91.07, df = 13 (P < 0.00001); I² = 86%
Test for overall effect: Z = 5.09 (P < 0.00001)

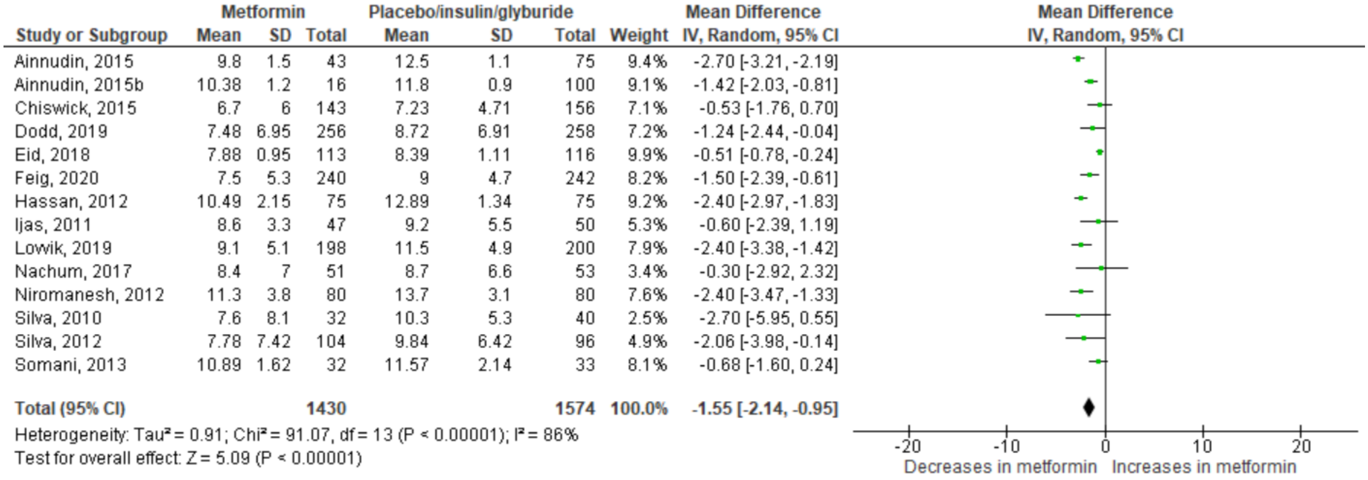

b) Pre-eclampsia

Study or Subgroup	Metformin Events	Metformin Total	Insulin/glyburide/placebo Events	Insulin/glyburide/placebo Total	Weight	Odds Ratio M-H, Random, 95% CI
Ainnudin, 2015	0	43	6	75	1.1%	0.12 [0.01, 2.24]
Ainnudin, 2015b	4	16	17	100	3.9%	1.63 [0.47, 5.66]
Borg, 2018	1	50	5	50	1.8%	0.18 [0.02, 1.63]
Chiswick, 2015	7	221	3	222	3.5%	2.39 [0.61, 9.36]
Dodd, 2019	13	256	11	258	5.8%	1.20 [0.53, 2.73]
Eid, 2018	5	113	6	116	4.0%	0.85 [0.25, 2.86]
Feig, 2020	37	242	30	240	7.5%	1.26 [0.75, 2.12]
Ijas, 2011	4	47	4	50	3.2%	1.07 [0.25, 4.55]
Jamal, 2012	2	35	4	35	2.5%	0.47 [0.08, 2.75]
Khan, 2018	17	385	60	385	7.3%	0.25 [0.14, 0.44]
Lowik, 2019	8	238	17	240	5.6%	0.46 [0.19, 1.08]
Moore, 2010	2	74	3	75	2.4%	0.67 [0.11, 4.11]
Nachum, 2017	2	51	5	53	2.6%	0.39 [0.07, 2.12]
Nascimento, 2019	8	127	31	145	5.8%	0.25 [0.11, 0.56]
Niromanesh, 2012	5	80	7	80	4.1%	0.70 [0.21, 2.29]
Rowan, 2008	20	363	26	370	7.0%	0.77 [0.42, 1.41]
Saleh, 2016	13	67	12	70	5.6%	1.16 [0.49, 2.77]
Spaulonci, 2013	5	14	3	12	2.6%	1.67 [0.30, 9.16]
Syngelaki, 2016	6	202	22	195	5.3%	0.24 [0.10, 0.61]
Tertti, 2013	5	110	10	107	4.4%	0.46 [0.15, 1.40]
Valdes, 2018	4	68	3	73	3.0%	1.46 [0.31, 6.77]
Vanky, 2010	10	135	5	135	4.5%	2.08 [0.69, 6.26]
Wasim, 2019	17	137	28	141	6.7%	0.57 [0.30, 1.10]
**Total (95% CI)**		**3074**		**3227**	**100.0%**	**0.69 [0.50, 0.95]**
Total events	195		318			

Heterogeneity: Tau² = 0.29; Chi² = 48.61, df = 22 (P = 0.0009); I² = 55%
Test for overall effect: Z = 2.26 (P = 0.02)

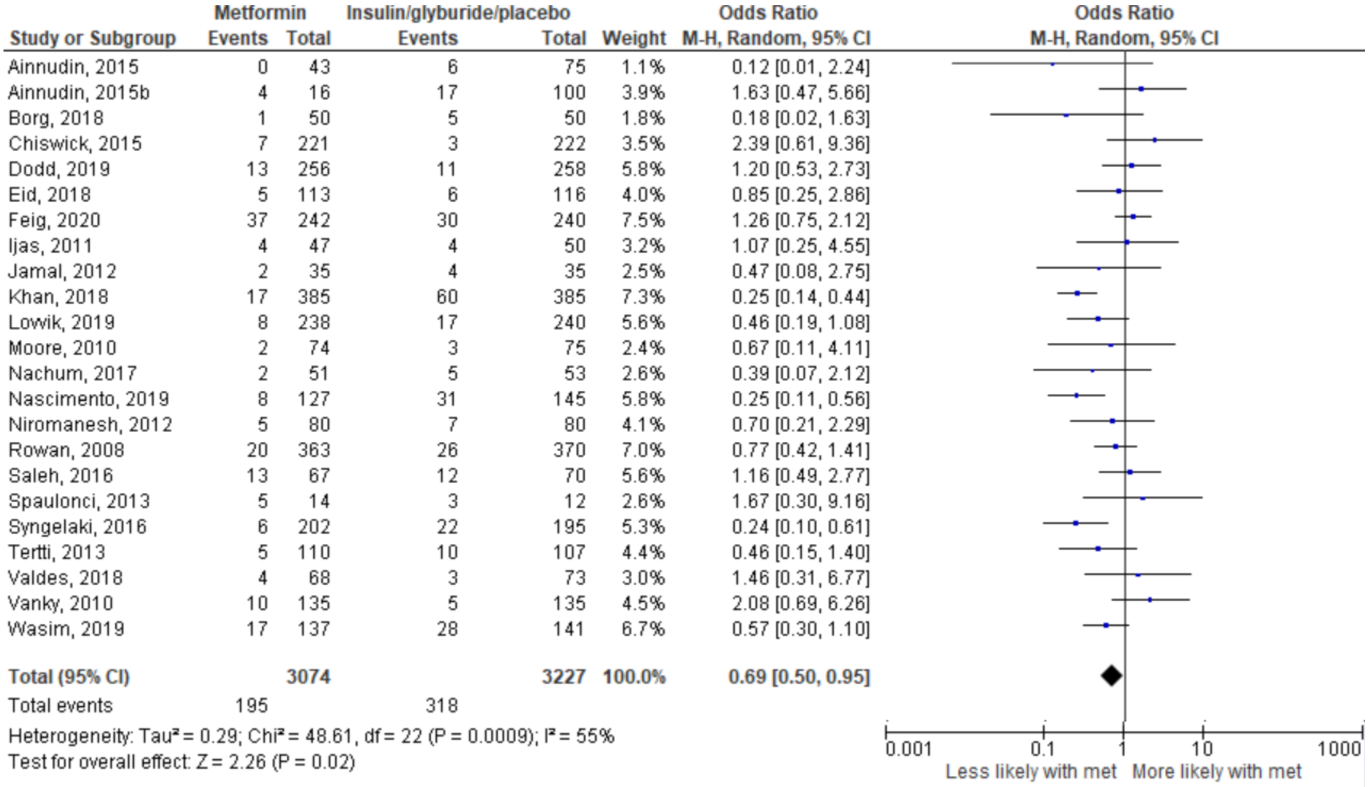

c) Gestational hypertension

Study or Subgroup	Metformin Events	Metformin Total	Placebo/insulin Events	Placebo/insulin Total	Weight	Odds Ratio M-H, Fixed, 95% CI
Ainnudin, 2015	8	43	18	75	6.2%	0.72 [0.28, 1.84]
Borg, 2018	3	50	4	50	2.2%	0.73 [0.16, 3.46]
Chiswick, 2015	21	221	14	222	7.3%	1.56 [0.77, 3.15]
Dodd, 2019	19	256	16	258	8.5%	1.21 [0.61, 2.41]
Eid, 2018	8	113	10	116	5.3%	0.81 [0.31, 2.13]
Feig, 2020	13	240	15	242	8.2%	0.87 [0.40, 1.86]
George, 2016	9	79	7	80	3.6%	1.34 [0.47, 3.80]
Khan, 2018	28	385	31	385	16.6%	0.90 [0.53, 1.52]
Lowik, 2019	16	238	13	240	7.0%	1.26 [0.59, 2.68]
Niromanesh, 2012	4	80	11	80	6.0%	0.33 [0.10, 1.09]
Rowan, 2008	14	363	23	370	12.7%	0.61 [0.31, 1.20]
Silva, 2012	10	104	7	96	3.8%	1.35 [0.49, 3.71]
Somani, 2013	0	32	0	33		Not estimable
Spaulonci, 2013	10	46	7	46	3.2%	1.55 [0.53, 4.50]
Syngelaki, 2016	13	202	13	195	7.2%	0.96 [0.43, 2.13]
Tertti, 2013	2	110	4	107	2.3%	0.48 [0.09, 2.66]
**Total (95% CI)**		**2562**		**2595**	**100.0%**	**0.95 [0.77, 1.18]**
Total events	178		193			

Heterogeneity: Chi² = 10.59, df = 14 (P = 0.72); I² = 0%
Test for overall effect: Z = 0.44 (P = 0.66)

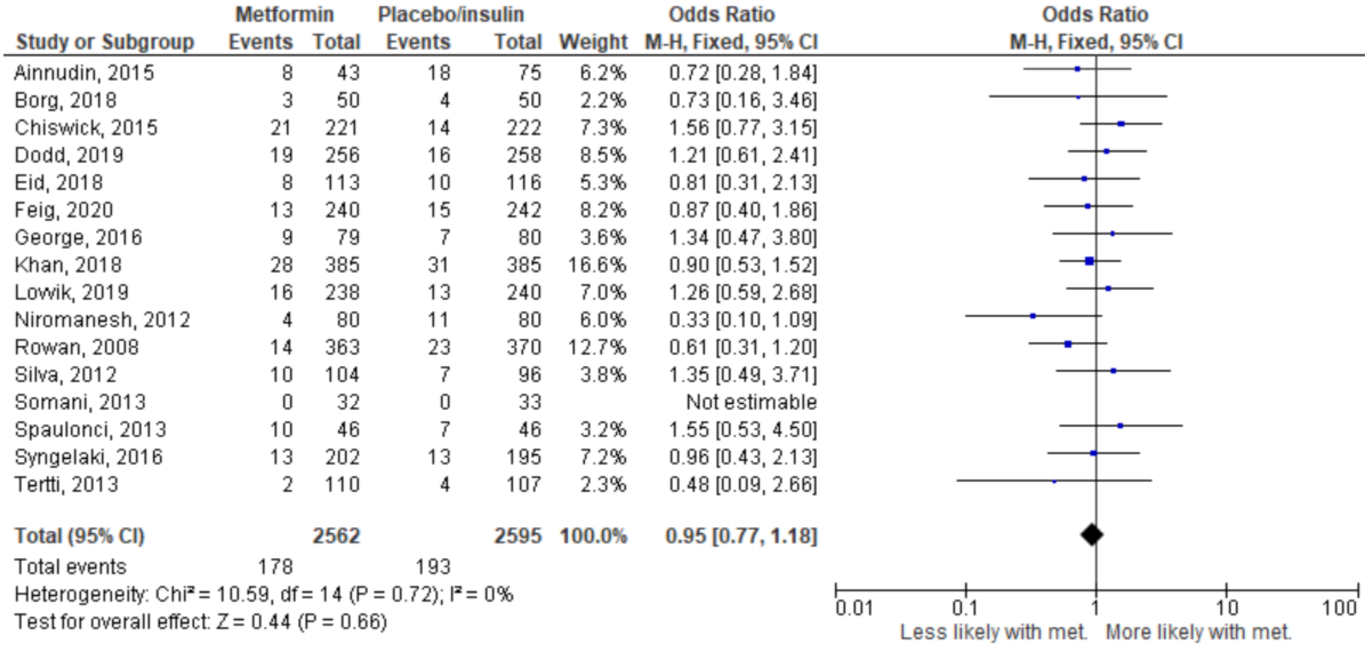

d) Pre-eclampsia and gestational hypertension (combined)

	Metformin		Insulin/glyburide/placebo			Odds Ratio
Study or Subgroup	Events	Total	Events	Total	Weight	M-H, Random, 95% CI
Ainnudin, 2015	8	86	24	150	4.2%	0.54 [0.23, 1.26]
Ainnudin, 2015b	4	16	17	100	2.5%	1.63 [0.47, 5.66]
Borg, 2018	4	100	9	100	2.6%	0.42 [0.13, 1.42]
Chiswick, 2015	28	444	17	442	5.9%	1.68 [0.91, 3.12]
Dodd, 2019	32	512	28	516	6.8%	1.16 [0.69, 1.96]
Eid, 2018	13	226	16	232	4.8%	0.82 [0.39, 1.75]
Feig, 2020	50	480	45	484	7.8%	1.13 [0.74, 1.73]
Ijas, 2011	4	47	4	50	2.0%	1.07 [0.25, 4.55]
Jamal, 2012	2	35	4	35	1.4%	0.47 [0.08, 2.75]
Khan, 2018	45	770	91	770	8.3%	0.46 [0.32, 0.67]
Lowik, 2019	24	482	30	483	6.5%	0.79 [0.46, 1.37]
Moore, 2010	2	74	3	75	1.3%	0.67 [0.11, 4.11]
Nachum, 2017	2	53	5	51	1.5%	0.36 [0.07, 1.95]
Nascimento, 2019	8	127	31	145	4.4%	0.25 [0.11, 0.56]
Niromanesh, 2012	9	160	18	160	4.3%	0.47 [0.20, 1.08]
Rowan, 2008	34	726	49	740	7.5%	0.69 [0.44, 1.09]
Saleh, 2016	13	67	12	70	4.1%	1.16 [0.49, 2.77]
Spaulonci, 2013	10	109	7	107	3.4%	1.44 [0.53, 3.94]
Syngelaki, 2016	19	404	34	396	6.3%	0.53 [0.29, 0.94]
Tertti, 2013	7	218	14	214	3.8%	0.47 [0.19, 1.20]
Valdes, 2018	4	68	3	73	1.8%	1.46 [0.31, 6.77]
Vanky, 2010	10	135	5	135	3.0%	2.08 [0.69, 6.26]
Wasim, 2019	17	137	28	141	5.6%	0.57 [0.30, 1.10]
**Total (95% CI)**		**5476**		**5669**	**100.0%**	**0.76 [0.60, 0.95]**
Total events	349		494			

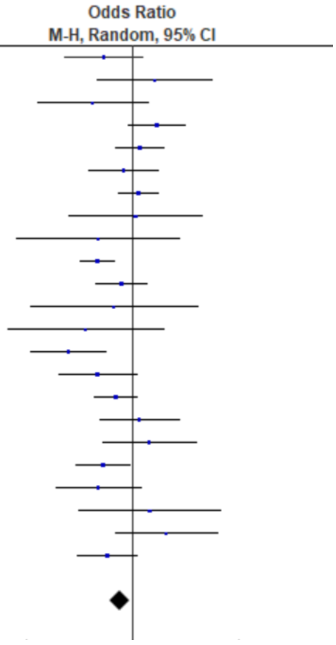

e) All cause preterm delivery

Study or Subgroup	Metformin Events	Metformin Total	Placebo/insulin/glyburide Events	Placebo/insulin/glyburide Total	Weight	Odds Ratio M-H, Random, 95% CI
Arshad, 2017	19	25	11	25	3.4%	4.03 [1.20, 13.53]
Ashoush, 2016	0	47	1	48	0.8%	0.33 [0.01, 8.39]
Borg, 2018	0	50	6	50	0.9%	0.07 [0.00, 1.24]
Chiswick, 2015	18	214	14	220	5.3%	1.35 [0.65, 2.79]
Dodd, 2019	13	256	18	258	5.2%	0.71 [0.34, 1.49]
Eid, 2018	8	113	7	116	3.9%	1.19 [0.42, 3.39]
Feig, 2020	60	240	47	242	6.6%	1.38 [0.90, 2.13]
Galal, 2019	7	56	4	50	3.1%	1.64 [0.45, 5.98]
George, 2016	3	79	1	80	1.4%	3.12 [0.32, 30.64]
Ghomian, 2019	20	143	19	143	5.5%	1.06 [0.54, 2.09]
Jamal, 2012	2	35	5	35	2.2%	0.36 [0.07, 2.02]
Khan, 2018	10	385	48	385	5.4%	0.19 [0.09, 0.38]
Lowik, 2019	9	238	18	240	4.8%	0.48 [0.21, 1.10]
Mesdaghinia, 2013	0	100	8	100	0.9%	0.05 [0.00, 0.95]
Nachum, 2017	6	51	4	53	3.0%	1.63 [0.43, 6.17]
Nascimento, 2019	9	127	9	145	4.3%	1.15 [0.44, 3.00]
Niromanesh, 2012	9	80	4	80	3.4%	2.41 [0.71, 8.17]
Rowan, 2008	44	363	28	370	6.3%	1.68 [1.02, 2.77]
Saleh, 2016	7	67	5	70	3.4%	1.52 [0.46, 5.04]
Silva, 2012	5	96	4	104	3.0%	1.37 [0.36, 5.27]
Somani, 2013	7	32	10	33	3.7%	0.64 [0.21, 1.97]
Spaulonci, 2013	5	47	5	47	3.1%	1.00 [0.27, 3.71]
Syngelaki, 2016	13	202	21	198	5.3%	0.58 [0.28, 1.19]
Tertti, 2013	6	109	4	107	3.1%	1.50 [0.41, 5.47]
Valdes, 2018	3	68	8	73	2.9%	0.38 [0.10, 1.48]
Vanky, 2010	5	135	11	135	3.8%	0.43 [0.15, 1.28]
Wasim, 2019	13	137	20	141	5.2%	0.63 [0.30, 1.33]
**Total (95% CI)**		**3495**		**3548**	**100.0%**	**0.90 [0.67, 1.21]**
Total events	301		340			

Heterogeneity: $Tau^2 = 0.30$; $Chi^2 = 61.15$, df = 26 (P = 0.0001); $I^2 = 57\%$
Test for overall effect: Z = 0.72 (P = 0.47)

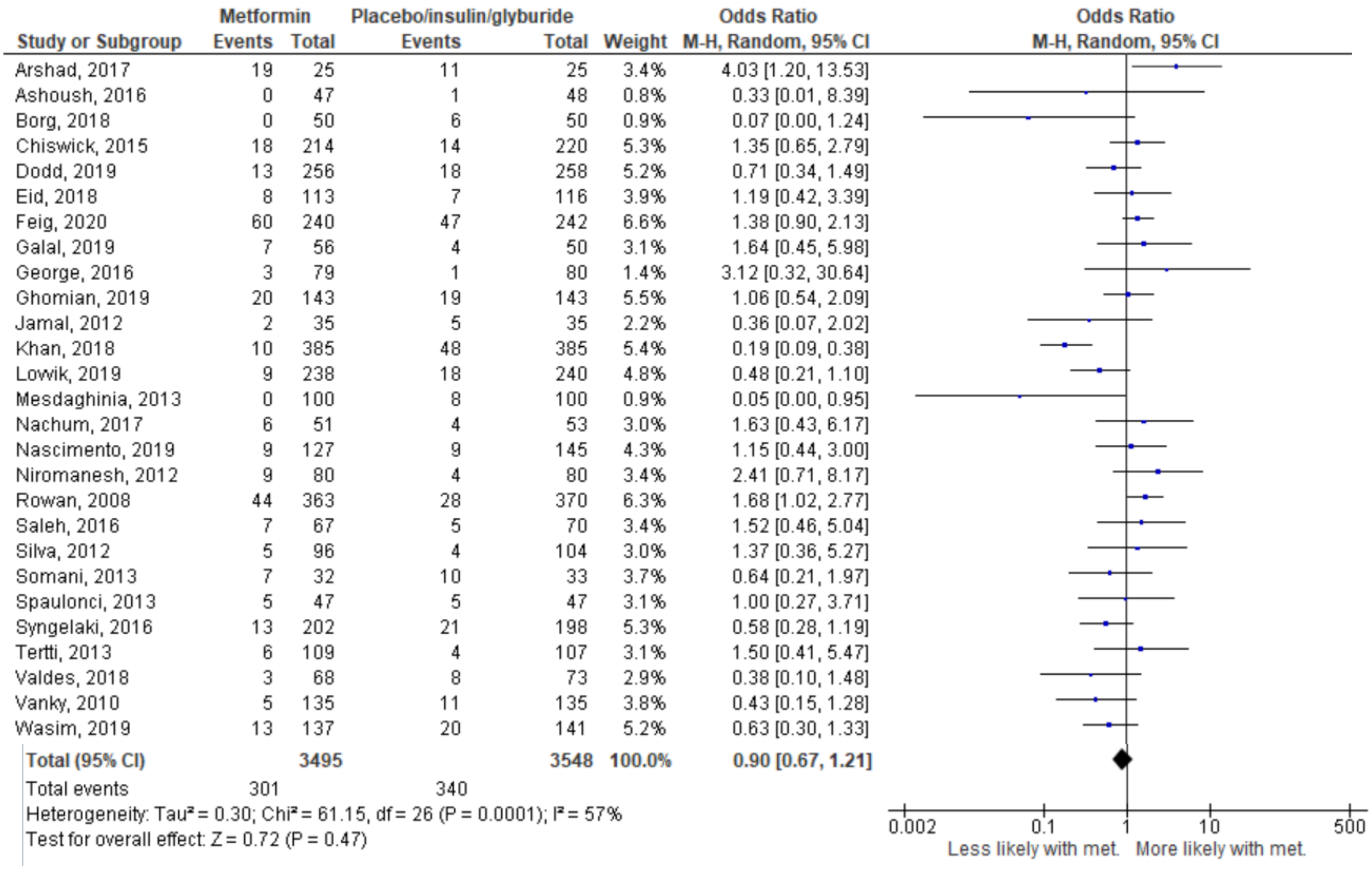

f) Gestational age at delivery

Study or Subgroup	Metformin Mean	Metformin SD	Metformin Total	Placebo/insulin/glyburide Mean	Placebo/insulin/glyburide SD	Placebo/insulin/glyburide Total	Weight	Mean Difference IV, Random, 95% CI
Ainnudin, 2015	37.4	1.6	43	37.8	1	75	4.4%	-0.40 [-0.93, 0.13]
Ainnudin, 2015b	36.19	1.68	16	37.06	1.22	100	2.1%	-0.87 [-1.73, -0.01]
Chiswick, 2015	39.4	2.27	214	39.5	1.67	220	6.7%	-0.10 [-0.48, 0.28]
Dodd, 2019	39.12	1.64	256	38.93	1.85	258	8.2%	0.19 [-0.11, 0.49]
Eid, 2018	37.2	1.9	113	37.9	1.4	116	5.7%	-0.70 [-1.13, -0.27]
Hassan, 2012	37.53	0.99	75	37.33	1.43	75	6.4%	0.20 [-0.19, 0.59]
Ibrahim, 2014	37.89	0.32	46	37.7	0.66	44	10.3%	0.19 [-0.03, 0.41]
Ijas, 2011	39.3	1.2	45	38.9	1.1	48	5.2%	0.40 [-0.07, 0.87]
Moore, 2007	37.9	2.5	32	38.1	1.4	31	1.6%	-0.20 [-1.20, 0.80]
Moore, 2010	38	2	75	38	1	74	4.7%	0.00 [-0.51, 0.51]
Nachum, 2017	37.6	1.2	51	38.1	1.5	53	4.5%	-0.50 [-1.02, 0.02]
Niromanesh, 2012	37.9	1	80	38	0.8	80	8.7%	-0.10 [-0.38, 0.18]
Silva, 2010	38.6	1.3	32	38.6	1.1	40	4.0%	0.00 [-0.56, 0.56]
Silva, 2012	38.25	1.41	104	38.41	1.17	96	7.1%	-0.16 [-0.52, 0.20]
Spaulonci, 2013	38.33	1.45	47	38.24	1.33	47	4.1%	0.09 [-0.47, 0.65]
Tertti, 2013	39.2	1.4	110	39.3	1.6	107	6.3%	-0.10 [-0.50, 0.30]
Wasim, 2019	37.5	1	137	37.6	1	141	9.8%	-0.10 [-0.34, 0.14]
**Total (95% CI)**			**1476**			**1605**	**100.0%**	**-0.07 [-0.21, 0.06]**

Heterogeneity: $Tau^2 = 0.03$; $Chi^2 = 30.08$, df = 16 (P = 0.02); $I^2 = 47\%$
Test for overall effect: Z = 1.05 (P = 0.29)

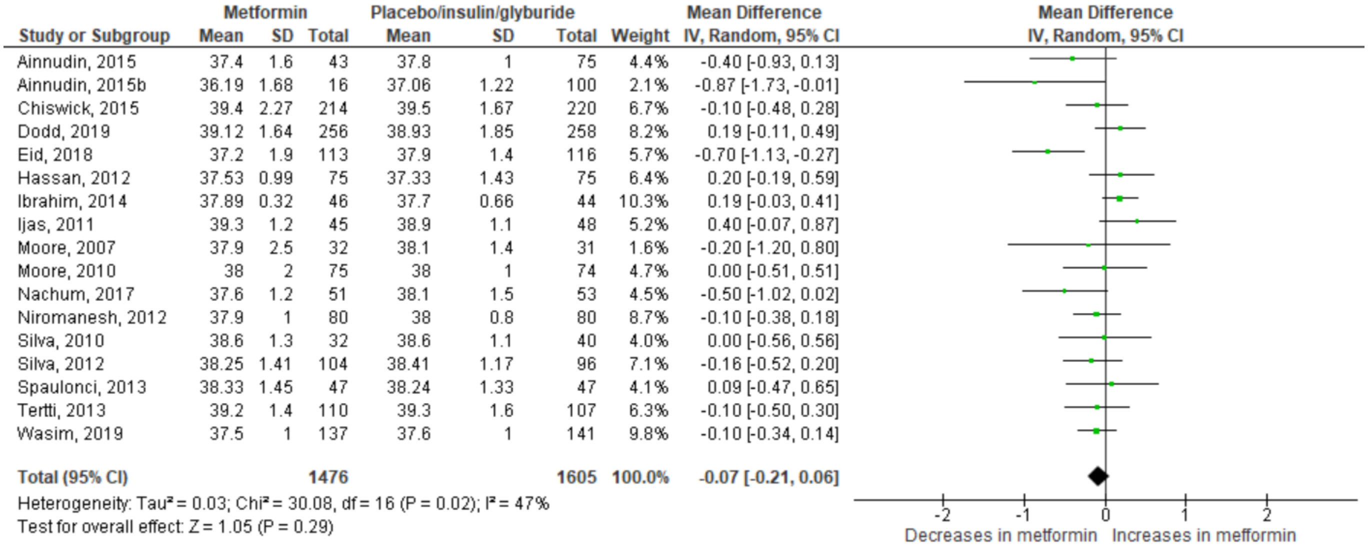

g) Cesarean-section: all reasons

Study or Subgroup	Metformin Events	Metformin Total	placebo/insulin/glyburide Events	placebo/insulin/glyburide Total	Weight	Odds Ratio M-H, Fixed, 95% CI
Ainnudin, 2015	18	43	38	75	2.0%	0.70 [0.33, 1.49]
Ainnudin, 2015b	3	16	18	100	0.5%	1.05 [0.27, 4.08]
Arshad, 2017	16	25	17	25	0.8%	0.84 [0.26, 2.70]
Ashoush, 2016	24	47	22	48	1.3%	1.23 [0.55, 2.76]
Borg, 2018	22	50	20	50	1.4%	1.18 [0.53, 2.61]
Chiswick, 2015	65	219	76	222	6.6%	0.81 [0.54, 1.21]
Dodd, 2019	87	256	111	258	9.1%	0.68 [0.48, 0.97]
Eid, 2018	42	113	49	116	3.8%	0.81 [0.48, 1.37]
Feig, 2020	125	234	148	236	8.5%	0.68 [0.47, 0.99]
Galal, 2019	30	52	44	54	2.3%	0.31 [0.13, 0.75]
George, 2016	31	79	28	80	2.1%	1.20 [0.63, 2.28]
Hassan, 2012	25	75	42	75	3.5%	0.39 [0.20, 0.76]
Ibrahim, 2014	30	43	27	39	1.1%	1.03 [0.40, 2.63]
Ijas, 2011	18	50	10	47	0.8%	2.08 [0.84, 5.15]
Khan, 2018	157	385	139	385	10.2%	1.22 [0.91, 1.63]
Lowik, 2019	45	238	45	240	4.5%	1.01 [0.64, 1.60]
Moore, 2007	7	32	10	31	1.0%	0.59 [0.19, 1.81]
Moore, 2010	11	75	2	74	0.2%	6.19 [1.32, 28.97]
Nachum, 2017	18	51	17	53	1.3%	1.16 [0.51, 2.61]
Niromanesh, 2012	34	80	37	80	2.6%	0.86 [0.46, 1.60]
Rowan, 2008	131	363	128	370	10.1%	1.07 [0.79, 1.45]
Saleh, 2016	27	67	30	70	2.2%	0.90 [0.46, 1.78]
Silva, 2010	22	32	28	40	1.0%	0.94 [0.34, 2.58]
Silva, 2012	68	104	66	96	3.0%	0.86 [0.48, 1.55]
Somani, 2013	24	32	23	33	0.7%	1.30 [0.44, 3.89]
Spaulonci, 2013	33	46	30	46	1.1%	1.35 [0.56, 3.27]
Syngelaki, 2016	80	202	82	195	6.3%	0.90 [0.61, 1.35]
Tertti, 2013	15	110	18	107	2.0%	0.78 [0.37, 1.64]
Valdes, 2018	27	68	35	73	2.5%	0.71 [0.37, 1.40]
Vanky, 2010	29	135	26	135	2.5%	1.15 [0.63, 2.08]
Wasim, 2019	76	137	93	141	5.1%	0.64 [0.40, 1.04]
**Total (95% CI)**		**3459**		**3594**	**100.0%**	**0.90 [0.82, 1.00]**
Total events	1340		1459			

Heterogeneity: Chi² = 39.17, df = 30 (P = 0.12); I² = 23%
Test for overall effect: Z = 2.03 (P = 0.04)

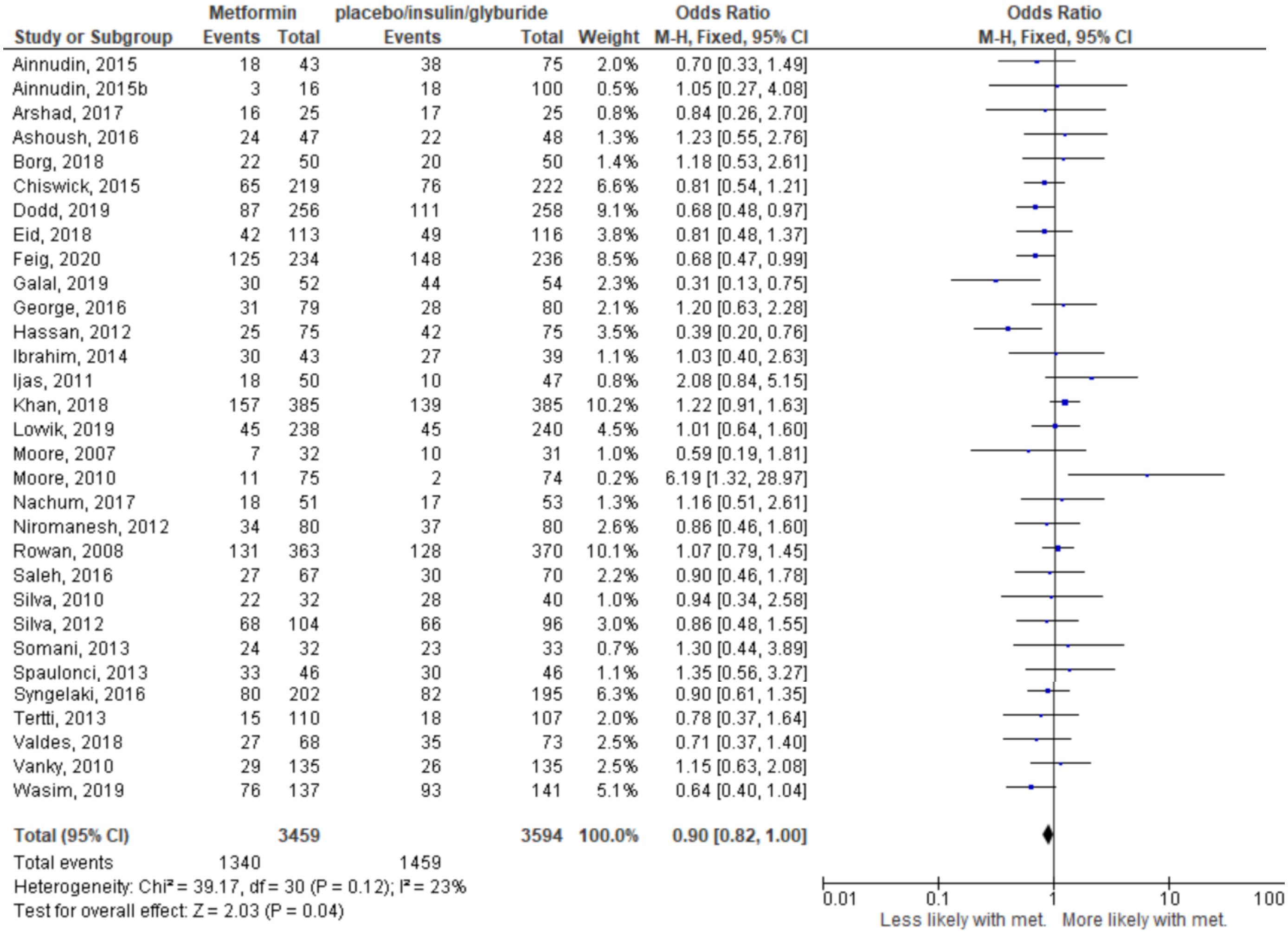

h) Emergency caesarean-section

Study or Subgroup	Metformin Events	Metformin Total	Placebo/insulin/glyburide Events	Placebo/insulin/glyburide Total	Weight	Odds Ratio M-H, Random, 95% CI
Dodd, 2019	43	256	57	258	20.8%	0.71 [0.46, 1.11]
Eid, 2018	29	113	31	116	17.2%	0.95 [0.53, 1.71]
Ijas, 2011	18	50	10	47	11.2%	2.08 [0.84, 5.15]
Lowik, 2019	30	238	35	248	18.7%	0.88 [0.52, 1.48]
Moore, 2010	11	75	2	74	5.2%	6.19 [1.32, 28.97]
Niromanesh, 2012	25	80	16	80	14.3%	1.82 [0.88, 3.75]
Saleh, 2016	14	67	16	70	12.7%	0.89 [0.40, 2.01]
**Total (95% CI)**		**879**		**893**	**100.0%**	**1.15 [0.78, 1.70]**
Total events	170		167			

Heterogeneity: Tau² = 0.14; Chi² = 13.15, df = 6 (P = 0.04); I² = 54%
Test for overall effect: Z = 0.72 (P = 0.47)

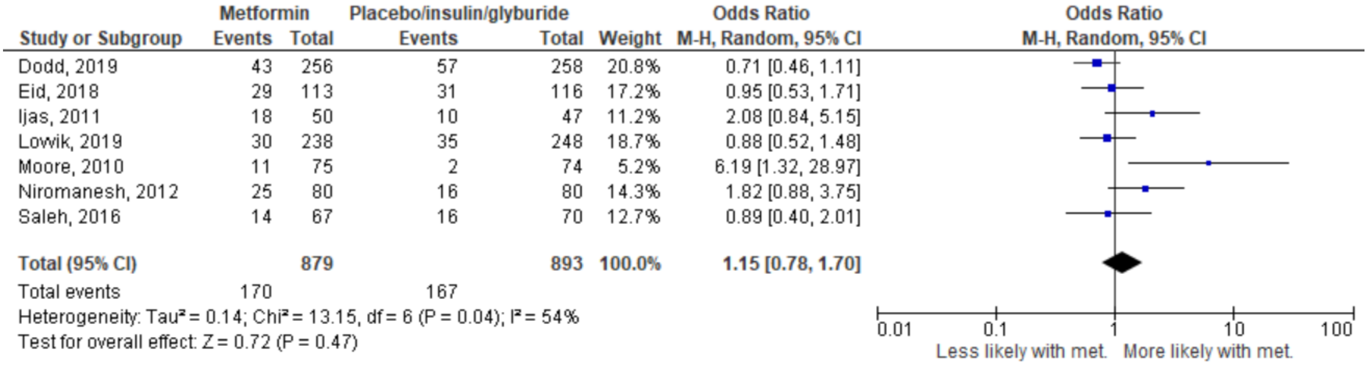

i) Elective caesarean-section

Study or Subgroup	Metformin Events	Metformin Total	Placebo/insulin/glyburide Events	Placebo/insulin/glyburide Total	Weight	Odds Ratio M-H, Fixed, 95% CI
Dodd, 2019	44	256	54	258	42.4%	0.78 [0.50, 1.22]
Eid, 2018	13	113	18	116	15.0%	0.71 [0.33, 1.52]
Lowik, 2019	15	238	10	248	8.7%	1.60 [0.70, 3.64]
Niromanesh, 2012	11	80	21	80	17.2%	0.45 [0.20, 1.00]
Saleh, 2016	27	67	30	70	16.7%	0.90 [0.46, 1.78]
**Total (95% CI)**		**754**		**772**	**100.0%**	**0.81 [0.61, 1.07]**
Total events	110		133			

Heterogeneity: Chi² = 4.94, df = 4 (P = 0.29); I² = 19%
Test for overall effect: Z = 1.48 (P = 0.14)

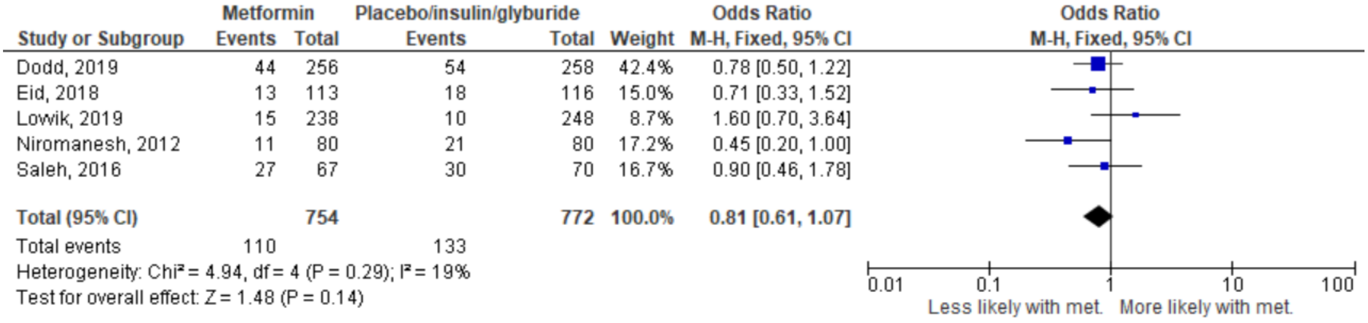

Supplementary Fig S8: New GDM development

Study or Subgroup	Metformin Events	Metformin Total	Placebo Events	Placebo Total	Weight	Odds Ratio M-H, Fixed, 95% CI
Chiswick, 2015	26	142	36	153	17.3%	0.73 [0.41, 1.28]
Dodd, 2019	72	256	62	258	27.1%	1.24 [0.83, 1.84]
Jamal, 2012	3	35	6	35	3.3%	0.45 [0.10, 1.98]
Lowik, 2019	60	238	57	240	25.9%	1.08 [0.71, 1.64]
Syngelaki, 2016	25	202	22	195	12.0%	1.11 [0.60, 2.04]
Valdes, 2018	18	48	16	63	5.3%	1.76 [0.78, 3.98]
Vanky, 2010	18	100	18	98	9.1%	0.98 [0.47, 2.01]
**Total (95% CI)**		**1021**		**1042**	**100.0%**	**1.07 [0.87, 1.33]**
Total events	222		217			

Heterogeneity: Chi² = 5.12, df = 6 (P = 0.53); I² = 0%
Test for overall effect: Z = 0.64 (P = 0.52)

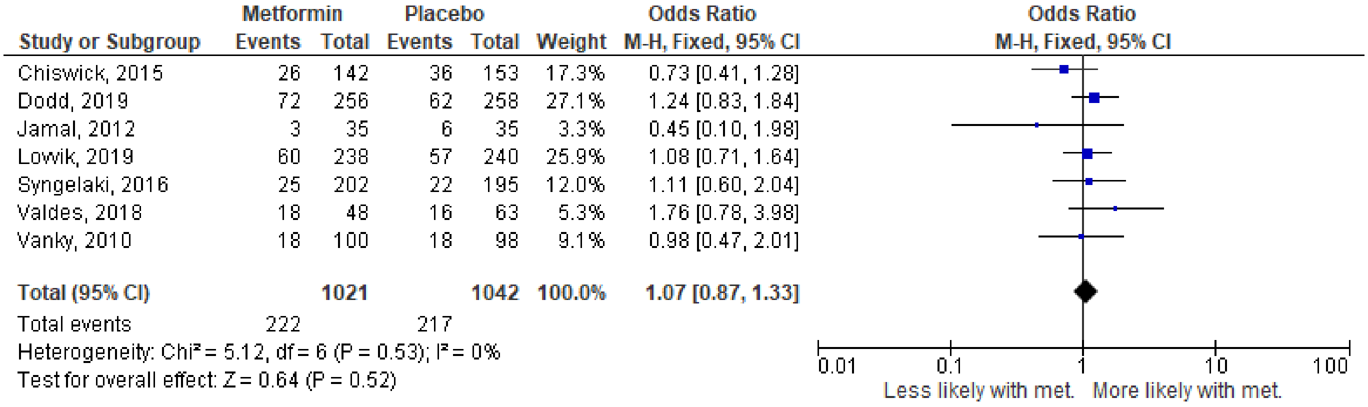

New GDM development
GDM=gestational diabetes mellitus; met=metformin.
Odds Ratio ± 95% CI. Fixed or random-effect model.

Supplementary Fig. S9: Glycaemic control: FBS

a) FBS (all studies)

Study or Subgroup	Metformin Mean	Metformin SD	Metformin Total	Insulin/glyburide Mean	Insulin/glyburide SD	Insulin/glyburide Total	Weight	Mean Difference IV, Random, 95% CI
Ainnudin, 2015	96.4	5.7	43	97.4	2.5	75	6.1%	-1.00 [-2.80, 0.80]
Ainnudin, 2015b	97.87	3.83	16	97.55	3.29	100	6.0%	0.32 [-1.66, 2.30]
Arshad, 2017	93.48	11.9	25	102.8	20.62	25	2.4%	-9.32 [-18.65, 0.01]
Ashoush, 2016	80.8	4.7	47	78.9	3.5	48	6.2%	1.90 [0.23, 3.57]
Borg, 2018	79	9.23	50	79.24	10.03	50	5.1%	-0.24 [-4.02, 3.54]
Eid, 2018	81.7	3.6	113	84.1	3.1	116	6.4%	-2.40 [-3.27, -1.53]
Feig 2020	95.3	13.9	75	97.7	13.7	65	4.6%	-2.40 [-6.98, 2.18]
Khan, 2018	82.28	5.51	385	76.88	7.75	385	6.4%	5.40 [4.45, 6.35]
Moore, 2007	92.6	10	31	96.8	12	32	4.1%	-4.20 [-9.65, 1.25]
Moore, 2010	94.3	15	75	90.9	13.3	74	4.6%	3.40 [-1.15, 7.95]
Nachum, 2017	91.3	8.8	51	88.7	10.2	53	5.1%	2.60 [-1.06, 6.26]
Niromanesh, 2012	88.3	7.7	80	88.7	6.3	80	6.0%	-0.40 [-2.58, 1.78]
Rowan, 2008	93.6	10.8	363	91.8	12.6	370	6.2%	1.80 [0.10, 3.50]
Saleh, 2016	93.25	13.7	67	94.33	11.1	70	4.8%	-1.08 [-5.27, 3.11]
Silva, 2010	78.2	8.9	32	87.7	12.1	40	4.4%	-9.50 [-14.35, -4.65]
Silva, 2012	88.53	11.71	104	90.52	11.78	96	5.4%	-1.99 [-5.25, 1.27]
Somani, 2013	85.41	5.96	32	82.27	5.57	33	5.6%	3.14 [0.33, 5.95]
Spaulonci, 2013	90.9	16.29	47	88.35	7.45	47	4.3%	2.55 [-2.57, 7.67]
Wasim, 2019	92.1	6	137	96.6	6.2	141	6.3%	-4.50 [-5.93, -3.07]
**Total (95% CI)**			**1773**			**1900**	**100.0%**	**-0.45 [-2.26, 1.36]**

Heterogeneity: Tau² = 13.05; Chi² = 233.68, df = 18 (P < 0.00001); I² = 92%
Test for overall effect: Z = 0.49 (P = 0.63)

Mean Difference IV, Random, 95% CI

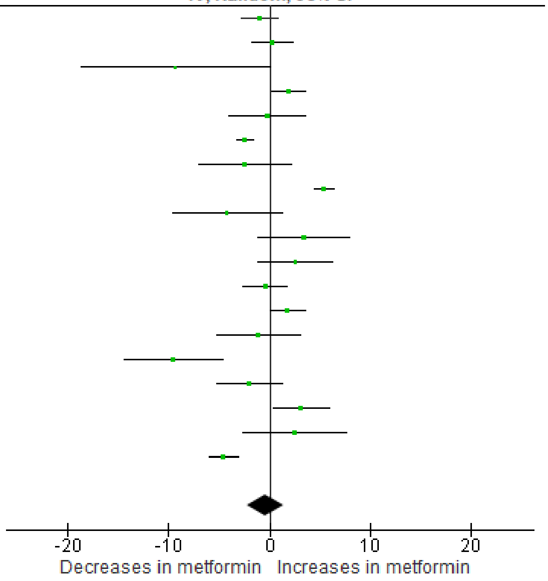

b) FBS: Metformin vs. insulin sub-group

Study or Subgroup	Metformin Mean	Metformin SD	Metformin Total	Insulin Mean	Insulin SD	Insulin Total	Weight	Mean Difference IV, Random, 95% CI
Ainnudin, 2015	96.4	5.7	43	97.4	2.5	75	7.6%	-1.00 [-2.80, 0.80]
Ainnudin, 2015b	97.87	3.83	16	97.55	3.29	100	7.5%	0.32 [-1.66, 2.30]
Arshad, 2017	93.48	11.9	25	102.8	20.62	25	2.9%	-9.32 [-18.65, 0.01]
Ashoush, 2016	80.8	4.7	47	78.9	3.5	48	7.7%	1.90 [0.23, 3.57]
Borg, 2018	79	9.23	50	79.24	10.03	50	6.3%	-0.24 [-4.02, 3.54]
Eid, 2018	81.7	3.6	113	84.1	3.1	116	8.0%	-2.40 [-3.27, -1.53]
Feig, 2020	95.3	13.9	75	97.7	13.7	65	5.7%	-2.40 [-6.98, 2.18]
Khan, 2018	82.28	5.51	385	76.88	7.75	385	8.0%	5.40 [4.45, 6.35]
Moore, 2007	92.6	10	31	96.8	12.2	32	5.0%	-4.20 [-9.70, 1.30]
Niromanesh, 2012	88.3	7.7	80	88.7	6.3	80	7.4%	-0.40 [-2.58, 1.78]
Rowan, 2008	93.6	10.8	363	91.8	12.6	370	7.7%	1.80 [0.10, 3.50]
Saleh, 2016	93.25	13.7	67	94.33	11.1	70	6.0%	-1.08 [-5.27, 3.11]
Somani, 2013	85.41	5.96	32	82.27	5.57	33	7.0%	3.14 [0.33, 5.95]
Spaulonci, 2013	90.9	16.29	47	88.35	7.45	47	5.3%	2.55 [-2.57, 7.67]
Wasim, 2019	92.1	6	137	96.6	6.2	141	7.8%	-4.50 [-5.93, -3.07]
**Total (95% CI)**			**1511**			**1637**	**100.0%**	**-0.26 [-2.26, 1.75]**

Heterogeneity: Tau² = 12.91; Chi² = 212.73, df = 14 (P < 0.00001); I² = 93%
Test for overall effect: Z = 0.25 (P = 0.80)

Mean Difference IV, Random, 95% CI

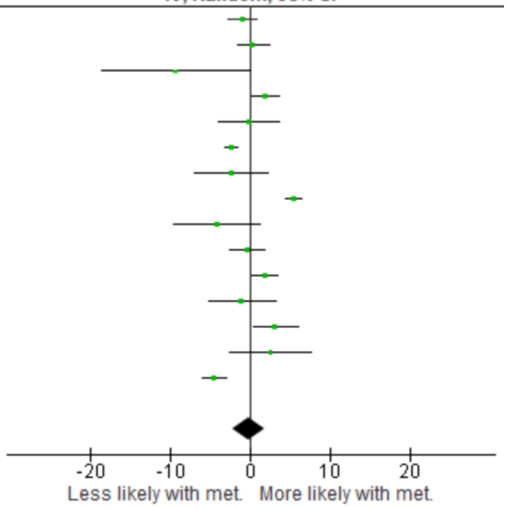

c) FBS: Metformin vs. glyburide sub-group

Study or Subgroup	Metformin Mean	Metformin SD	Metformin Total	Glyburide Mean	Glyburide SD	Glyburide Total	Weight	Mean Difference IV, Fixed, 95% CI
Moore, 2010	94.3	15	75	90.9	13.3	74	18.6%	3.40 [-1.15, 7.95]
Nachum, 2017	91.3	8.8	51	88.7	10.2	53	28.8%	2.60 [-1.06, 6.26]
Silva, 2010	78.2	8.9	32	88.7	12.1	40	16.3%	-10.50 [-15.35, -5.65]
Silva, 2012	88.53	11.71	104	90.52	11.78	96	36.3%	-1.99 [-5.25, 1.27]
**Total (95% CI)**			**262**			**263**	**100.0%**	**-1.06 [-3.02, 0.91]**

Heterogeneity: Chi² = 22.37, df = 3 (P < 0.0001); I² = 87%
Test for overall effect: Z = 1.06 (P = 0.29)

Mean Difference IV, Fixed, 95% CI

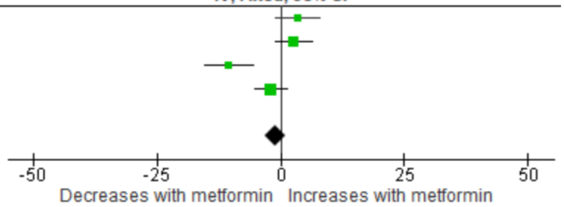

Supplementary Fig S10: Glycaemic: control: RBS

a) RBS

Study or Subgroup	Metformin Mean	Metformin SD	Metformin Total	Insulin/glyburide Mean	Insulin/glyburide SD	Insulin/glyburide Total	Weight	Mean Difference IV, Random, 95% CI
Ainnudin, 2015	129.6	8.5	43	128.1	6.4	75	6.4%	1.50 [-1.42, 4.42]
Ainnudin, 2015b	136.79	5.34	16	135.31	4.64	100	6.6%	1.48 [-1.29, 4.25]
Ashoush, 2016	112.2	6.8	47	111	5.2	48	7.0%	1.20 [-1.24, 3.64]
Borg, 2018	110.72	8.15	50	112.36	8.08	50	6.1%	-1.64 [-4.82, 1.54]
Eid, 2018	95.9	4.7	113	101.4	4.8	116	8.2%	-5.50 [-6.73, -4.27]
Feig 2020	113.2	18.2	68	114.4	13.8	57	3.8%	-1.20 [-6.82, 4.42]
Khan, 2018	111.24	7.02	385	112.34	5.02	385	8.4%	-1.10 [-1.96, -0.24]
Moore, 2007	104.6	5.1	32	104.4	19.4	33	3.0%	0.20 [-6.65, 7.05]
Moore, 2010	109.7	17.6	75	111.7	19.3	74	3.6%	-2.00 [-7.93, 3.93]
Nachum, 2017	112.6	12.3	51	115.3	13.8	53	4.3%	-2.70 [-7.72, 2.32]
Niromanesh, 2012	111.3	9.1	80	111.5	9	80	6.6%	-0.20 [-3.00, 2.60]
Rowan, 2008	111.6	10.6	363	115.2	16.2	370	7.5%	-3.60 [-5.58, -1.62]
Saleh, 2016	116.5	3.53	67	117.12	3.45	70	8.2%	-0.62 [-1.79, 0.55]
Silva, 2010	136	23.7	32	129.1	20.8	40	1.6%	6.90 [-3.54, 17.34]
Silva, 2012	126.44	16.91	104	126.48	20.51	96	4.1%	-0.04 [-5.27, 5.19]
Somani, 2013	121.38	11	32	113.08	11.71	33	3.9%	8.30 [2.78, 13.82]
Spaulonci, 2013	108.44	13.14	47	112.3	12.35	47	4.2%	-3.86 [-9.02, 1.30]
Wasim, 2019	120	13.6	137	128	10	141	6.6%	-8.00 [-10.81, -5.19]
**Total (95% CI)**			**1742**			**1868**	**100.0%**	**-1.18 [-2.64, 0.28]**

Heterogeneity: Tau² = 6.43; Chi² = 97.44, df = 17 (P < 0.00001); I² = 83%
Test for overall effect: Z = 1.58 (P = 0.11)

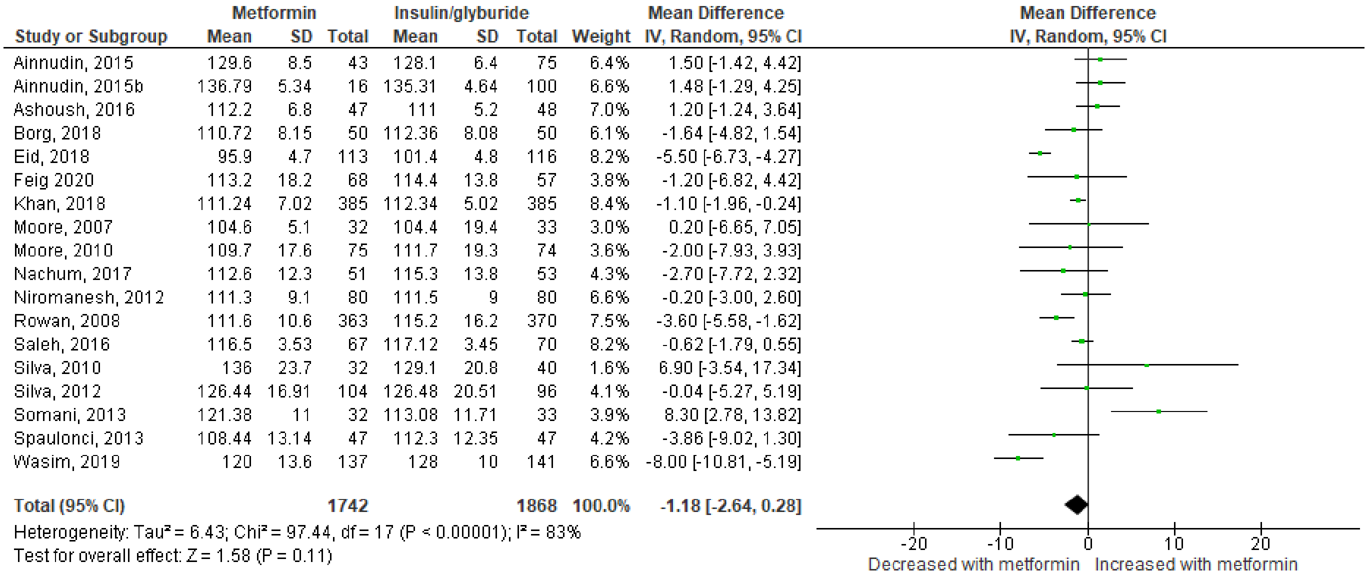

b) RBS (metformin vs. insulin sub-group)

Study or Subgroup	Metformin Mean	Metformin SD	Metformin Total	Insulin Mean	Insulin SD	Insulin Total	Weight	Mean Difference IV, Random, 95% CI
Ainnudin, 2015	129.6	8.5	43	128.1	6.4	75	8.4%	1.50 [-1.42, 4.42]
Ainnudin, 2015b	136.79	5.34	16	135.31	4.64	100	8.7%	1.48 [-1.29, 4.25]
Ashoush, 2016	112.2	6.8	47	111	5.2	48	9.3%	1.20 [-1.24, 3.64]
Borg, 2018	110.72	6.8	50	111	5.2	50	9.5%	-0.28 [-2.65, 2.09]
Eid, 2018	102.6	23.8	113	107.3	26.3	116	3.6%	-4.70 [-11.19, 1.79]
Khan, 2018	111.24	7.02	385	112.34	5.02	385	12.0%	-1.10 [-1.96, -0.24]
Niromanesh, 2012	111.3	9.1	80	111.5	9	80	8.6%	-0.20 [-3.00, 2.60]
Rowan, 2008	111.6	10.6	363	115.2	16.2	370	10.2%	-3.60 [-5.58, -1.62]
Saleh, 2016	116.5	3.53	67	117.12	3.45	70	11.6%	-0.62 [-1.79, 0.55]
Somani, 2013	121.38	11	32	113.08	11.71	33	4.5%	8.30 [2.78, 13.82]
Spaulonci, 2013	108.44	13.14	47	112.3	12.35	47	4.9%	-3.86 [-9.02, 1.30]
Wasim, 2019	120	13.6	137	128	10	141	8.6%	-8.00 [-10.81, -5.19]
**Total (95% CI)**			**1380**			**1515**	**100.0%**	**-0.92 [-2.39, 0.54]**

Heterogeneity: Tau² = 4.47; Chi² = 53.89, df = 11 (P < 0.00001); I² = 80%
Test for overall effect: Z = 1.23 (P = 0.22)

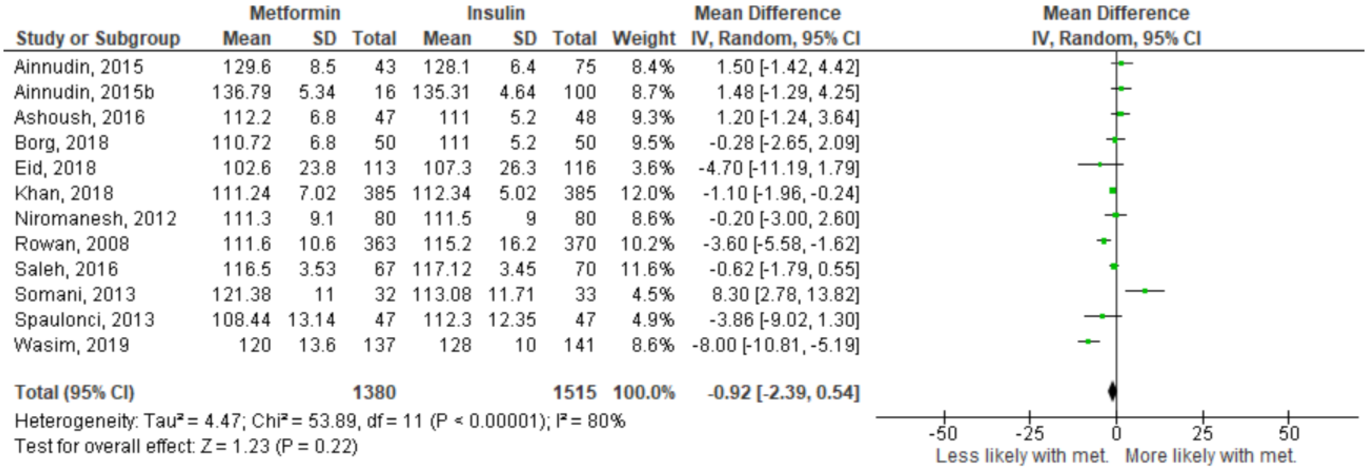

c) RBS (metformin vs. glyburide sub-group)

Study or Subgroup	Metformin Mean	Metformin SD	Metformin Total	Glyburide Mean	Glyburide SD	Glyburide Total	Weight	Mean Difference IV, Fixed, 95% CI
Moore, 2010	109.7	17.6	75	111.7	19.3	74	25.0%	-2.00 [-7.93, 3.93]
Nachum, 2017	112.6	12.3	51	115.3	13.8	53	34.9%	-2.70 [-7.72, 2.32]
Silva, 2010	136	23.7	32	129.1	20.8	40	8.1%	6.90 [-3.54, 17.34]
Silva, 2012	126.44	16.91	104	126.48	20.51	96	32.1%	-0.04 [-5.27, 5.19]
**Total (95% CI)**			**262**			**263**	**100.0%**	**-0.90 [-3.86, 2.07]**

Heterogeneity: Chi² = 2.87, df = 3 (P = 0.41); I² = 0%
Test for overall effect: Z = 0.59 (P = 0.55)

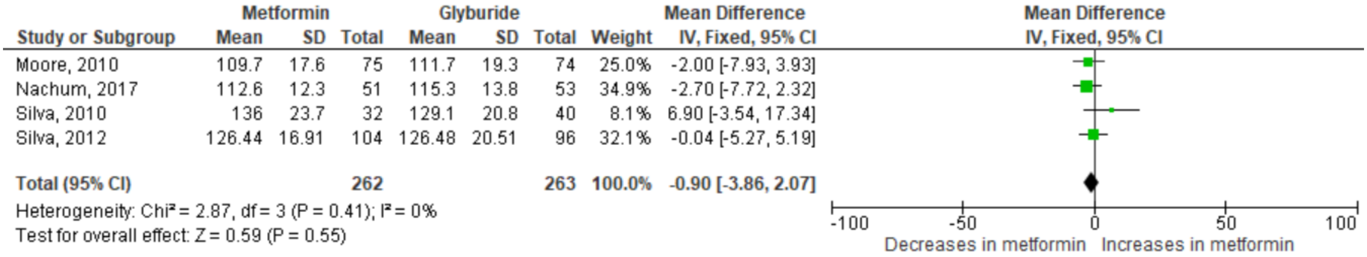

OR=Odds Ratio; 95% confidence intervals
RBS=random blood glucose; met-metformin

Supplementary Fig S11: Maternal hypoglycaemia

a) All studies

Study or Subgroup	Metformin Events	Metformin Total	Insulin/glyburide Events	Insulin/glyburide Total	Weight	Odds Ratio M-H, Fixed, 95% CI
Ashoush, 2016	3	48	6	47	13.4%	0.46 [0.11, 1.94]
Feig, 2020	7	232	6	238	13.5%	1.20 [0.40, 3.63]
Ibrahim, 2014	3	46	10	46	22.0%	0.25 [0.06, 0.98]
Moore, 2010	2	75	1	74	2.3%	2.00 [0.18, 22.54]
Somani, 2013	1	32	3	33	6.7%	0.32 [0.03, 3.28]
Wasim, 2019	6	137	19	141	42.1%	0.29 [0.11, 0.76]
**Total (95% CI)**		**570**		**579**	**100.0%**	**0.47 [0.28, 0.80]**
Total events	22		45			

Heterogeneity: Chi² = 6.00, df = 5 (P = 0.31); I² = 17%
Test for overall effect: Z = 2.81 (P = 0.005)

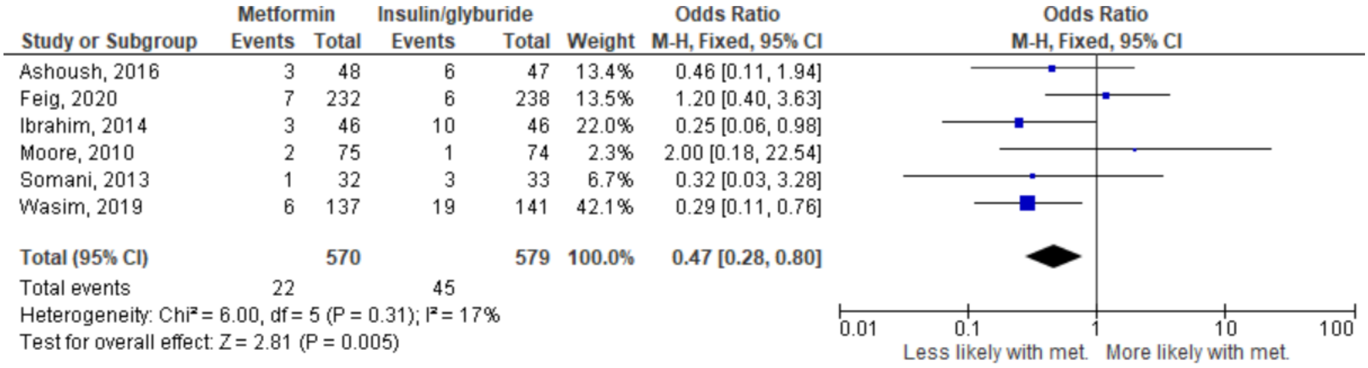

b) Metformin vs. insulin only

Study or Subgroup	Metformin Events	Metformin Total	Insulin Events	Insulin Total	Weight	Odds Ratio M-H, Fixed, 95% CI
Ashoush, 2016	3	48	6	47	15.9%	0.46 [0.11, 1.94]
Ibrahim, 2014	3	46	10	46	26.1%	0.25 [0.06, 0.98]
Somani, 2013	1	32	3	33	8.0%	0.32 [0.03, 3.28]
Wasim, 2019	6	137	19	141	50.0%	0.29 [0.11, 0.76]
**Total (95% CI)**		**263**		**267**	**100.0%**	**0.31 [0.16, 0.60]**
Total events	13		38			

Heterogeneity: Chi² = 0.38, df = 3 (P = 0.95); I² = 0%
Test for overall effect: Z = 3.49 (P = 0.0005)

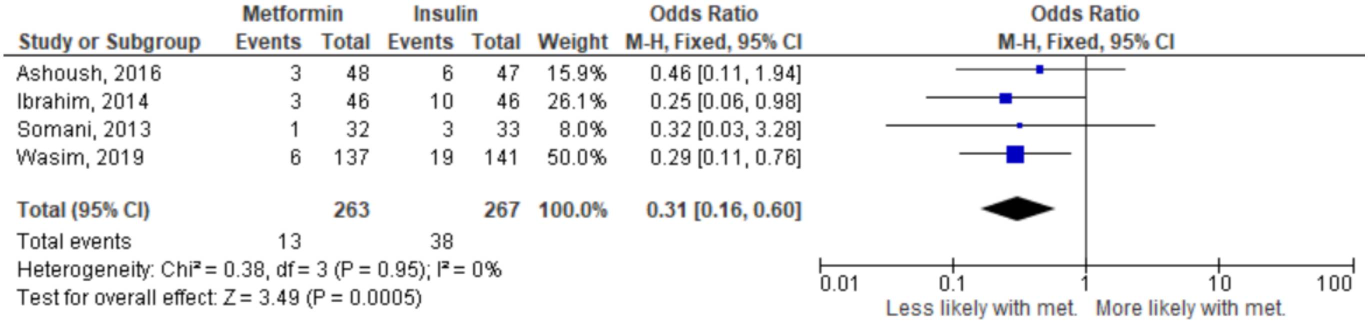

Supplementary Table S1: Results of heterogeneity analysis and calculation of prediction intervals

Outcome	Test of moderators	Residual heterogeneity	Cochran's Q	I^2	H^2	95% prediction interval
Gestational weight gain	1.99 p=0.57	67.47 p<0.001	90.75 p<0.0001	80%	4.99	-3.17-0.05
Pre-eclampsia	8.96† P=0.26	24.63 P=0.06	48.28 P=0.001	53%	2.13	0.24-1.99
PIH	4.92 P=0.55	5.41 P=0.80	9.82 p=0.83	0%	1.00	0.78-1.19
Preterm birth	6.02 p=0.42	47.11 p=0.003	60.31 p<0.0001	56%	2.31	0.31-2.64
Gestational age at delivery	4.37 p=0.36	18.43 p=0.10	30.06 p=0.02	48%	1.93	-0.48-0.32
Cesarean section	3.57 P=0.73	33.66 P=0.09	38.71 P=0.13	18%	1.23	0.66-1.18

Results of heterogeneity analysis and calculation of prediction intervals

The test of moderators was performed by specifying a random-effects meta-regression with categorical moderators for both treatment indication (GDM, obesity, PCOS, diabetes in pregnancy) and comparator group (placebo, glyburide, insulin). The reported value is the result of the omnibus test for the effect of these moderators (Q_M). Additional checks were performed to ensure that the individual levels of each moderator also returned non-significant impacts on the meta-regression results. † GDM was a significant moderator in the context of pre-eclampsia only (p=0.034). The residual heterogeneity is calculated from the same meta-regression model (Q_E). Cochran's Q, I^2, and H^2 values are obtained from the random-effects meta-analysis with all sub-groups combined, as are the 95% prediction intervals.

Supplementary Table S2: Inclusion/Exclusion table

	Title/Abstract	Screening	Full Text	Screening
	Inclusion criteria	Exclusion criteria	Inclusion criteria	Exclusion criteria
**Study design**	• Human studies.<br>• >50 cases.	• Animal studies.<br>• < 50 cases.<br>• < 10 cases for metformin group.<br>• Non-primary research articles (including reviews).<br>• Editorial comments, meeting abstracts (with insufficient data), book chapters, non-peer review articles.	• Human studies<br>• >50 cases<br>• Randomised controlled studies and prospective randomised controlled studies.	• Animal studies<br>• < 50 cases<br>• <10 cases for metformin group.<br>• Non-primary research articles (including reviews).<br>• Editorial comments, meeting abstracts (with insufficient data), book chapters, non-peer review articles.
**Group**	• Pregnant women with metformin intervention only.	• Pregnant women randomised to metformin not in combination with any other trial drug	• Women with any indication requiring metformin during pregnancy.<br>• Singleton pregnancies.	• Pregnant women randomised to metformin not in combination with any other trial drug
**Exposure**	• Metformin vs. other pharmacological intervention AND/OR diet AND/OR lifestyle.		• Metformin vs. other drug and/or diet/lifestyle for pregnant women.	
**Outcome**	• 'Baseline' maternal parameters recorded before study start and/or at follow-up<br>OR<br>• Pregnancy and delivery complications recorded (e.g. gestational hypertension, pre-eclampsia, preterm birth, side-effects, mode of delivery, glycaemic control, GDM incidence).		• Maternal parameters recorded before study start and/or after study/follow-up.<br>OR<br>• Pregnancy and delivery complications recorded (e.g. gestational hypertension, pre-eclampsia, preterm birth, side-effects, mode of delivery, glycaemic control, GDM incidence).	

Supplementary Table S3: Risk of Bias

Study or Subgroup	A	B	C	D	E	F	G
	Risk of Bias						
Ainnudin, 2015	−	?	−	?	+	+	+
Ainnudin, 2015b	−	?	−	?	+	?	−
Arshad, 2017	−	?	−	?	?	?	+
Ashoush, 2016	+	?	−	−	+	+	?
Borg, 2018	+	+	−	?	+	+	+
Chiswick, 2015	+	+	+	+	?	+	+
Dodd, 2019	+	+	+	+	+	+	+
Eid, 2018	?	?	−	?	−	+	+
Feig 2020	+	?	+	+	+	+	?
Galal, 2019	?	?	−	?	?	?	?
George, 2016	+	+	?	+	+	+	?
Ghomian, 2019	?	?	?	?	?	?	+
Hassan, 2012	−	?	−	?	+	+	+
Ibrahim, 2014	?	?	−	?	?	+	+
Ijas, 2011	+	+	−	?	+	+	+
Jamal, 2012	+	+	−	?	+	+	?
Khan, 2018	+	?	−	?	+	?	−
Lowik, 2019	+	+	+	+	?	+	−
Mesdaghinia, 2013	+	?	−	+	−	+	+
Moore, 2007	+	+	−	−	+	+	+
Moore, 2010	?	?	−	?	?	?	?
Nachum, 2017	+	+	?	?	+	+	+
Nascimento, 2020	+	?	?	?	−	+	?
Niromanesh, 2012	+	+	−	+	+	+	+
Rowan, 2008	+	?	−	?	+	+	+
Saleh, 2016	?	?	−	?	−	+	+
Silva, 2010	+	+	+	?	?	+	+
Silva, 2012	+	+	+	?	+	?	+
Somani, 2013	+	?	−	−	+	+	+
Spaulonci, 2013	+	?	−	?	+	+	+
Syngelaki, 2016	+	?	+	?	+	+	+
Tertti, 2013	+	+	−	?	+	+	+
Valdes, 2018	?	?	?	?	−	?	+
Vanky, 2010	+	+	?	?	+	+	+
Wasim, 2019	?	?	−	?	?	+	+

Risk of bias legend
(A) Random sequence generation (selection bias)
(B) Allocation concealment (selection bias)
(C) Blinding of participants and personnel (performance bias)
(D) Blinding of outcome assessment (detection bias)
(E) Incomplete outcome data (attrition bias)
(F) Selective reporting (reporting bias)
(G) Other bias

Risk of Bias analysis
Green circles=low risk of bias; yellow circles=unknown risk of bias; red circles=high risk of bias

Supplementary Table 4: Study characteristics

This table is found as an Excel Spreadsheet

Supplementary Table S5: Heterogeneity of GDM/PCOS/maternal obesity diagnosis

**Paper citation**	**GDM/*PCOS* diagnosis criteria**
27,53	ACOG
23,25,26,39,47,48	ADA
37	ADIPS
28,34,36,41	CC
40	FNC
24	IADPSG
42,45	NDDG
38,44,46,49,51,54	WHO
35	UNSPECIFIED
31,50	*Rotterdam*
33	*Rotterdam & NIH*
29,30,32	*Maternal obesity (≥ 30kg/m^2)*

Heterogeneity diagnosis for GDM/PCOS/maternal obesity diagnosis
ADA=American Diabetes Association; ADIPS=Australasian Diabetes in Pregnancy Society; CC=Carpenter-Coustan; FNC=Finnish National Criteria; GDM=gestational diabetes mellitus; IADPSG= International Association of Diabetes and Pregnancy Study Groups; NDDG= National Diabetes Diagnosis Group; NIH=National Institute of Health; PCOS=polycystic ovary syndrome; WHO=World Health Organisation

Supplementary Table S6 Fig: Eggers Test

**Outcome**	**Comparison**	**Effect**	**Eggers test**	**P value**
GWG	Metformin vs. all	***	0.223	0.824
GWG	Metformin vs. placebo	*	2.339	*0.029*
GWG	Metformin vs. insulin	***	0.037	0.971
Pre-eclampsia	Metformin vs. all	*	-0.716	0.474
Pre-eclampsia	Metformin vs. placebo	N/S	1.351	0.177
Pre-eclampsia	Metformin vs. insulin	0.08	-0.391	0.696
Gestational age at delivery	Metformin vs. insulin	N/S	-1.32	0.190
Preterm birth	Metformin vs. all	N/S	-1.637	0.102
C-section	Metformin vs. all	N/S	-0.115	0.909
GDM	Metformin vs. placebo (maternal obesity)	N/S	-0.996	0.190

Eggers Testing for publication bias
C-section=Cesarean-section; GDM=Gestational Diabetes Mellitus; GWG=gestational weight gain

Supplementary Table S7: Effect of metformin treatment upon side effects (vs. placebo: PCOS and maternal obesity)

Outcome		Unadjusted OR (95% CI)	P value	Studies	N	Het. I_2	Het. P value
**Nausea**	Placebo	1.44 (1.13-1.84)	.003	4	1441	0%	.51
**Vomiting**	Placebo	1.42 (1.10-1.84)	.008	4	1441	7%	.36
**Diarrhoea**	Placebo	2.73 (1.59-4.68)	.0003	4	1441	68%	.02
**Abdominal pain**	Placebo	1.00 (0.75-1.33)	.98	4	1242	0%	.45
**Bloating**	Placebo	1.32 (0.73-2.38)	.36	1	240	N/A	N/A
**Constipation**	Placebo	1.11 (0.76-1.63)	.59	2	797	15%	.28
**Headache**	Placebo	1.17 (0.82-1.69)	.39	2	797	69%	.07

Likelihood of side effects in PCOS and maternal obesity pregnancies treated with metformin OR= Odds Ratio ± 95% CI. Het=Heterogeneity

Supplementary Table S8: Gastrointestinal side effects in women with diabetes in pregnancy randomised to metformin.

First Author		Raw averages (%)	N
**Ainnudin, 2015**	GI side effects	7 (8)	93
	Stopped medication	6 (6)	93
**Ashoush, 2016**	GI side effects	14 (30)	47
	Stopped medication	0 (0)	47
**Ijas, 2011**	GI side effects	3 (6)	50
	Stopped medication	3 (6)	50
**Niromanesh, 2012**	GI side effects	6 (8)	80
	Stopped medication	3 (4)	80
**Rowan, 2008**	GI side effects	32 (9)	363
	Stopped medication	7 (2)	363
**Spaulonci, 2013**	GI side effects	21 (46)	46
	Stopped medication	1 (2)	46
**Tertti, 2013**	GI side effects	2 (2)	110
	Stopped medication	2 (2)	110
**Wasim, 2020**	GI side effects	4 (3)	137
	Stopped medication	4 (3)	137
**Weighted average**	GI side effects	12.5	929
**Incidence (%)**	Stopped medication	14.3	929

Supplementary S1 Text: PROSPERO document

NIHR | National Institute for Health Research

PROSPERO
International prospective register of systematic reviews

Is metformin use in pregnancy associated with an increased likelihood of maternal complications?

Jane Tarry-Adkins, Catherine Aiken, Susan Ozanne

Citation

Jane Tarry-Adkins, Catherine Aiken, Susan Ozanne. Is metformin use in pregnancy associated with an increased likelihood of maternal complications?. PROSPERO 2020 CRD42020167692 Available from: https://www.crd.york.ac.uk/prospero/display_record.php?ID=CRD42020167692

Review question

Is metformin use in pregnancy associated with an increased likelihood of maternal complications? Is there a higher risk of preterm birth, pre-eclampsia, or differences in maternal weight gain when treated with metformin compared to no treatment or other pharmacological therapies? Are there any sub-group differences in pregnancies treated for different indications or where treatment is commenced at different stages in pregnancy?

Searches

PubMed, Ovid Embase, MEDLINE, Web of Science, the Cochrane Library and clintrials.gov

Database search date ranges: * End date 1st Feb 2020 (search end date will alter and will reflect a date just before paper submission)

PubMed: Start date: June 1997 to *

Ovid Embase: Start date: 1974 to *

MEDLINE: Start date: 1946 to *

Web of Science: Start date: 1900 to *

The Cochrane Library: Start date: Database inception to *

Clin.trials.gov: Start date: Database inception to *

No restrictions for publication dates or language have been/will be made in these searches. No filters were/are used during these searches.

PubMed search strategy example:
("metformin"[MeSH Terms] OR "metformin"[All Fields])

AND

("metformin"[MeSH Terms] OR "metformin"[All Fields]) AND ("diabetes, gestational"[MeSH Terms] OR ("diabetes"[All Fields] AND "gestational"[All Fields]) OR "gestational diabetes"[All Fields] OR ("gestational"[All Fields] AND "diabetes"[All Fields] AND "mellitus"[All Fields]) OR "gestational diabetes mellitus"[All Fields])

OVID EMBASE search strategy example:

1. metformin.mp.

2. metformin.ti, ab.

3. exp *metformin/

4. 2 or 3

5. (gestation* adj3 diabet*).ti, ab.

6 exp *pregnancy diabetes mellitus/

7. 5 or 6

8. 4 and 7

Note: At the piloting of search selection process, addition of other indications such as "polycystic ovary syndrome" and /or "maternal obesity" in the search terms resulted in poor search outcomes and therefore was not used.

Inclusion criteria:

- All languages.

- Human studies, > 50 cases.

- Randomised controlled and prospective randomised controlled studies.

- Pregnant women with metformin intervention.

- Metformin vs, other pharmacological intervention AND/OR diet AND/OR lifestyle for pregnant women.

- Outcomes: 'Baseline' maternal parameters recorded before the study start and/or follow-up AND/OR pregnancy and delivery complications recorded (including gestational hypertension, pre-eclampsia and preterm birth).

Exclusion criteria:

- Non human studies, < 50 cases.

- Non primary research articles (including reviews).

- Editorial comments, meeting abstracts (with insufficient data), book chapters & non-peer review articles.

- Exclusion of participants based on fetal/birth weight.

Types of study to be included

Included: Randomised controlled and prospective randomised controlled studies.

Excluded: Studies which are not randomised such as retrospective studies.

Condition or domain being studied

Preterm birth, pre-eclampsia, and maternal weight gain after maternal metformin treatment for gestational diabetes (GDM), polycystic ovarian syndrome (PCOS), obesity, or other conditions.

Participants/population

All pregnancies treated with metformin.

Inclusion criteria:

- All languages

Supplementary S1 Text (page 2)

4. 2 or 3

5. (gestation* adj3 diabet*).ti, ab.

6 exp *pregnancy diabetes mellitus/

7. 5 or 6

8. 4 and 7

Note: At the piloting of search selection process, addition of other indications such as "polycystic ovary syndrome" and /or "maternal obesity" in the search terms resulted in poor search outcomes and therefore was not used.

Inclusion criteria:

- All languages.

- Human studies, > 50 cases.

- Randomised controlled and prospective randomised controlled studies.

- Pregnant women with metformin intervention.

- Metformin vs, other pharmacological intervention AND/OR diet AND/OR lifestyle for pregnant women.

- Outcomes: 'Baseline' maternal parameters recorded before the study start and/or follow-up AND/OR pregnancy and delivery complications recorded (including gestational hypertension, pre-eclampsia and preterm birth).

Exclusion criteria:

- Non human studies, < 50 cases.

- Non primary research articles (including reviews).

- Editorial comments, meeting abstracts (with insufficient data), book chapters & non-peer review articles.

- Exclusion of participants based on fetal/birth weight.

Types of study to be included

Included: Randomised controlled and prospective randomised controlled studies.

Excluded: Studies which are not randomised such as retrospective studies.

Condition or domain being studied

Preterm birth, pre-eclampsia, and maternal weight gain after maternal metformin treatment for gestational diabetes (GDM), polycystic ovarian syndrome (PCOS), obesity, or other conditions.

Participants/population

All pregnancies treated with metformin.

Inclusion criteria:

- All languages

- Human studies, > 50 cases.

- Randomised controlled and prospective randomised controlled studies.

- Pregnant women with metformin intervention.

- Metformin vs, other pharmacological intervention AND/OR diet AND/OR lifestyle for pregnant women.

- Outcomes: 'Baseline' maternal parameters recorded before the study start and/or follow-up AND/OR pregnancy and delivery complications recorded (including gestational hypertension, pre-eclampsia and preterm birth).

Exclusion criteria:

- Non human studies, < 50 cases.

- Non primary research articles (including reviews).

- Editorial comments, meeting abstracts (with insufficient data), book chapters & non-peer review articles.

- Exclusion of participants based on fetal/birth weight.

Intervention(s), exposure(s)

Metformin intervention during pregnancy.

Comparator(s)/control

Dependent upon the study, the reference group will be insulin-treated, diet-therapy, other pharmacological agents (such as glyburide), placebo, or un-treated women.

Context

Metformin is an oral glucose-lowering-agent, increasingly used in pregnancy, yet as it crosses the placenta; uncertainty exists regarding its use for indications during pregnancy. It is endorsed as an acceptable, economic alternative to insulin for gestational diabetes (GDM) treatment by national bodies and is increasingly used for other indications, including obesity and polycystic-ovarian-syndrome (PCOS), during pregnancy. GDM affects ~3%-25% of pregnancies worldwide, (1/3 of which will require drug therapy for glycaemic control). With obesity and maternal-age increasing in the maternity population, this rate is expected to continue to rise. It is difficult to fully estimate the numbers of women with PCOS as diagnosis is challenging, however global estimates show 3-10% of the female population are affected. New trials of metformin in pregnancy are planned in low-middle human-development-index countries. In these settings the high incidence of GDM (>25%) could result in ~10% of the pregnant population being prescribed metformin. Given the increasing scale of intrauterine metformin-exposure, studies investigating the potential effects on both mother and her unborn child are warranted. Preterm delivery is a commonplace pregnancy-complication with ~ 60, 000 babies/year in the UK born at < 37 gestational-weeks. Prematurity is associated with risk of still-birth, perinatal, neonatal, and infant-mortality, with survivors having increased risk of long-term disability. Pre-eclampsia and other hypertensive-disorders-of-pregnancy are common adverse outcomes leading to significant maternal morbidity. Gestational-weight-gain is an important influence on health during pregnancy, and for the mother's life-course health. This meta-analysis aims to elucidate the effect of metformin-exposure in pregnancy on common maternal adverse pregnancy-outcomes.

Main outcome(s)

Maternal outcomes: (Prenatal and perinatal)

• Preterm birth: (delivery < 37 weeks); (n values and %); (dichotomous data).

• Gestational age at delivery (weeks); (n values, mean, ± SD); (continuous data).

• Pre-eclampsia: (where threshold detailed: BP > 140/90mm/Hg with proteinurea >300mg/24hr); (n values and %), (dichotomous data).

• Pregnancy-induced hypertension: (where threshold detailed: BP >140/90 mm/Hg); (n values & %), (dichotomous data).

• Gestational weight gain (kg), (n value, mean ± SD); (continuous data).

• Other maternal outcomes.

* Measures of effect

Main outcomes will be assessed as continuous/dichotomous variables in the specified units, at all ages reported after or before delivery.

Additional outcome(s)

Maternal outcomes: (Prenatal and perinatal)

• Mode of delivery (n values and % - dichotomous data) or (n values, mean and ± SD - continuous data).

• Maternal glycaemic control:(n values and % - dichotomous data) or (n values, mean and ± SD - continuous data).

Postnatal outcomes:

• Later postnatal outcomes: (n values and % - dichotomous data) or (n values, mean and ± SD - continuous data).

* Measures of effect

Secondary outcomes will be assessed as continuous/dichotomous variables in the specified units, at all ages reported after or before delivery.

Data extraction (selection and coding)

PubMed, Ovid Embase, MEDLINE, Web of Science and The Cochrane Library will be searched systematically, after which the papers will be screened on Title and Abstract, by two reviewers independently. The full texts of these selected studies will be independently assessed using inclusion and exclusion criteria. Disagreement over the eligibility will be discussed with a third reviewer.

We intend to extract the following data: author, year of publication, country, sample size, exposure unit (mg), duration of exposure to metformin, diagnostic criteria for GDM/PCOS/obesity or other conditions, population randomisation criteria, reported outcomes including maternal baseline characteristics pregnancy (including duration of gestation), delivery and neonatal outcomes.

Risk of bias (quality) assessment

The quality of studies will be assessed using the modified Cochrane Collaboration tool to assess risk of bias for randomized controlled trials. Bias is assessed as a judgment (high, low, or unclear) for individual elements from seven domains: (selection (randomisation), selection (concealment), performance, detection, attrition, reporting, and other). This assessment will be performed by two reviewers independently. Disagreement between reviewers regarding the quality of a study will be discussed with a third reviewer.

Strategy for data synthesis

To synthesise and analyse quantitative data, a systematic review/meta-analysis will be conducted using R. Heterogeneity will be assessed with Galbraith plots, and the decision to use a fixed-effect or random-effects model will based on this analysis. Data will be graphically displayed using forest plots. Additionally, meta-regression will be performed to explore the effects of heterogeneity in terms of study-level covariates. Publication bias will be assessed using funnel plots, plotting the effects sizes against standard errors.

Analysis of subgroups or subsets

Supplementary S2 Text: Database search terms

PubMed:
Initial search date: 19.11.19. (Search date range: June 1997 to 19.11.19).
Basic search terms: Metformin AND Gestational diabetes mellitus
("metformin"[MeSH Terms] OR "metformin"[All Fields]) AND ("diabetes, gestational"[MeSH Terms] OR ("diabetes"[All Fields] AND "gestational"[All Fields]) OR "gestational diabetes"[All Fields] OR ("gestational"[All Fields] AND "diabetes"[All Fields] AND "mellitus"[All Fields]) OR "gestational diabetes mellitus"[All Fields])

Web of Science:
Initial search date: 19.11.19. (Search date range: 1900 to 19.11.19).
Basic search terms: Metformin AND Gestational diabetes mellitus

OVID EMBASE
Search date range: 1974 to 19.11.19.
metformin.mp.
metformin.ti,ab.
exp*metformin/
2 or 3
(gestation*adj3 diabet*).ti,ab.
exp*pregnancy diabetes mellitus/
5 or 6
4 and 7

OVID MEDLINE
Search date range: 1946 to 19.11.19.
metformin.mp.
metformin.ti,ab.
exp*metformin/
2 or 3
(gestation*adj3 diabet*).ti,ab.
4 and 5

The Cochrane Database
Search date range: Database inception to 19.11.19.
Basic search terms: Metformin AND Gestational diabetes mellitus

www.clinical trials.gov
Search date range: Database inception to 19.11.19.
Basic search terms: Metformin AND gestational diabetes
